# Molecular motor-functionalized porphyrin macrocycles

**DOI:** 10.1038/s41467-020-19123-y

**Published:** 2020-10-20

**Authors:** Pieter J. Gilissen, Paul B. White, José Augusto Berrocal, Nicolas Vanthuyne, Floris P. J. T. Rutjes, Ben L. Feringa, Johannes A. A. W. Elemans, Roeland J. M. Nolte

**Affiliations:** 1 Institute for Molecules and Materials, Radboud University, Heyendaalseweg 135, 6525 AJ Nijmegen, The Netherlands; 2 Stratingh Institute for Chemistry, University of Groningen, Nijenborgh 4, 9747 AG Groningen, The Netherlands; 3 Aix Marseille Univ, CNRS, Centrale Marseille, iSm2, Marseille, France

**Keywords:** Molecular capsules, Synthesis and processing, Nanocavities

## Abstract

Molecular motors and switches change conformation under the influence of an external stimulus, e.g. light. They can be incorporated into functional systems, allowing the construction of adaptive materials and switchable catalysts. Here, we present two molecular motor-functionalized porphyrin macrocycles for future photo-switchable catalysis. They display helical, planar and point chirality, and are diastereomers, which differ in the relative orientation of the motor and macrocyclic components. Fluorescence, UV-vis, and ^1^H NMR experiments reveal that the motor-functionalized macrocycles can bind and thread different variants of viologen guests, including a one-side blocked polymeric one of 30 repeat units. The latter feature indicates that the motor systems can find the open end of a polymer chain, thread on it, and move along the chain to eventually bind at the viologen trap, opening possibilities for catalytic writing on single polymer chains via chemical routes.

## Abstract (web summary)

Molecular motors and switches change conformation under the influence of an external stimulus and can be incorporated into functional systems, allowing the construction of adaptive materials and switchable catalysts. Here, the authors present two molecular motor-functionalized porphyrin macrocycles for future photo-switchable catalysis.

## Introduction

**Fig. 1:**
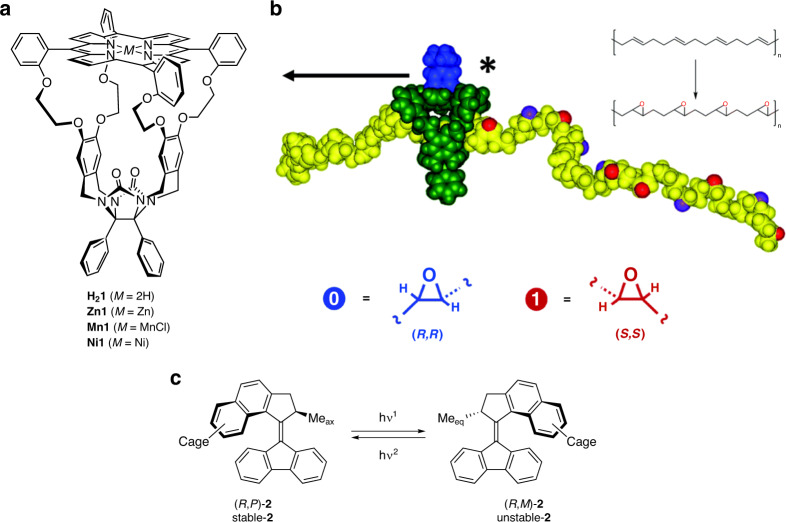
Structure of compounds. **a** Molecular structure of porphyrin cage compounds **H**_**2**_**1**, **Zn1**, **Mn1**, and **Ni1**. **b** Schematic representation of the process in which a chiral derivative of **Mn1** (green) writes chiral epoxides (blue and red balls) on a polymer comprising (*E*)-alkene functions (yellow) yielding a series of binary codes (reproduced with permission from the Royal Society of Chemistry)^17^. **c** Structure of the second-generation molecular motor **2**.

Since the first report of a synthetic motor converting external energy into unidirectional rotary motion in the late 1990s^1^, a variety of motor structures have been synthesized and applied as molecular machines, chiral switches, and catalysts^2–4^. Examples include rotary motors that stereoselectively bind chiral anion guests in a dynamic way^5^, motors that control the helicity of polyisocyanates^6^, polyphenylacetylenes^7^, and Cu(I) helicates^8^, and motors that act as switchable catalysts, e.g., in asymmetric Michael additions^9^, in Henry reactions^10^, in desymmetrization reactions^11^, and in asymmetric addition reactions^12^. Modified motors containing peptide chains have also been used to photodestabilize membranes^13^. Furthermore, motors have been incorporated in metal organic frameworks, in which they retained their rotary function^14^. Here we report the synthesis and properties of a second generation light-driven motor that is attached to a porphyrin macrocycle based on the glycoluril framework (compound **H**_**2**_**1**; Fig. 1a). The latter macrocycle has a cavity, which can selectively thread and bind viologen guest molecules including polymeric ones. The motor-functionalized porphyrin macrocycle displays various elements of chirality, i.e., point, helical, and planar chirality, which is a relatively rare combination of stereoelements in supramolecular chemistry^15^. This study is part of a more general project aimed at the development of a photo-switchable chiral catalyst that can thread onto a polymer chain, e.g., polybutadiene, and move along it while converting the alkene double bonds in epoxides (Fig. 1b)^16,17^. The function of the motor (Fig. 1c) is not to act as a rotary motor, but as a photo-switch that changes its helicity and thereby the chiral environment of the catalyst, i.e., in such a way that either (*R*,*R*)- or (*S*,*S*) epoxides are formed. Photo-switches have been attached before to macrocyclic systems, examples being crown ethers, calixarenes, cyclodextrins, pillararenes, and cucurbiturils^18^. They have been used to control the host–guest binding properties of these systems, e.g., the light-powered unidirectional threading of a small-molecule guest through a macrocyclic host^19^ and their catalytic properties^20^. The ultimate goal of our project is the construction of a photo-switchable machine that can write digital information, i.e., (*R*,*R*)-epoxide is digit 0 and (*S*,*S*)- epoxide is digit 1, on a polymer chain, which is of interest for data storage^21^.

## Results

### Synthesis and characterization

**Fig. 2:**
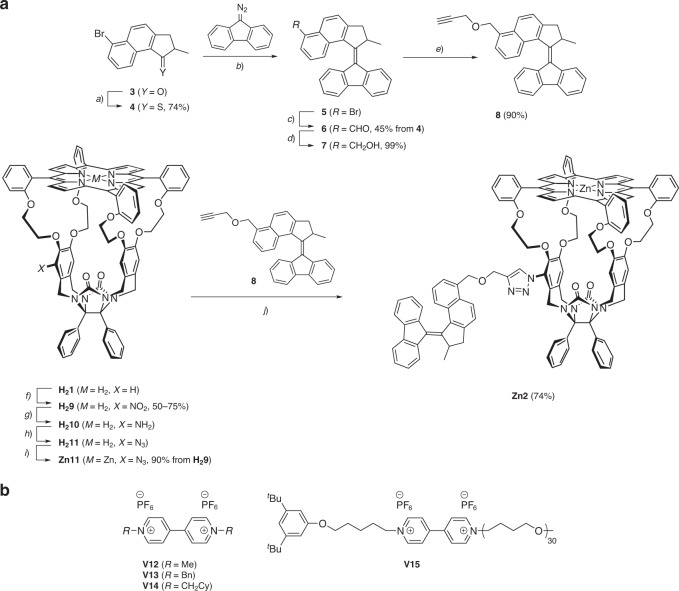
Synthesis and chemical structures of motor-porphyrin macrocycle conjugate and guests. **a** Synthesis of **Zn2**. Reagents and conditions: **a** Lawesson’s reagent, PhMe, reflux, 2 h; **b** PhMe, reflux, 16 h; **c** ^*n*^BuLi, THF, −78 °C, 1 h; then DMF, THF, −78 °C → 20 °C, 1 h; **d** NaBH_4_, DCM/MeOH, 20 °C, 15 min; **e** NaH, THF, 0 °C, 1 h; then propargyl bromide, THF, 0 °C → 20 °C, 21 h; **f** HNO_3_, CHCl_3_, −50 °C → −40 °C, 4 h; **g** SnCl_2_, HCl, dioxane, 60 °C, 5 h; **h** ^*t*^BuONO, TMSN_3_, CHCl_3_/MeCN, 20 °C, 1.5 h; **i** Zn(OAc)_2_·2H_2_O, CHCl_3_/MeOH, 20 °C, 20 h; **j** CuI, DIPEA, DMSO/H_2_O, 20 °C, 3 days. **b** Structures of viologen guest compounds (Cy = cyclohexyl).

For ease of synthesis, it was decided to connect the second-generation motor element to the porphyrin macrocycle via click chemistry. The route to the relevant conjugate (**Zn2**) is depicted in Fig. 2. The molecular motor part was prepared starting from bromoketone **3**^22,23^, which was treated with Lawesson’s reagent to give thioketone **4** in reasonable yield. A Barton–Kellogg reaction between **4** and 9-diazofluorene furnished the bromo-functionalized molecular motor **5**^24^, which turned out to be contaminated with inseparable bifluorenylidene. This mixture was treated with *n*-butyllithium and the resulting lithiated molecular motor was reacted with *N,N*-dimethylformamide (DMF), affording aldehyde **6** in 45% yield over two steps from thioketone **4**. At this stage, the bifluorenylidene side product could be separated and removed by column chromatography. Compound **6** was then reduced with sodium borohydride to give benzylic alcohol **7** in quantitative yield. Using sodium hydride as a base, the alcohol was alkylated to furnish propargyl ether **8** in excellent yield. Each of the compounds **5**–**8** displayed the typical ^1^H nuclear magnetic resonance (NMR) splitting pattern that is observed for second-generation molecular motors with a related molecular structure^22,24,25^.

**Fig. 3:**
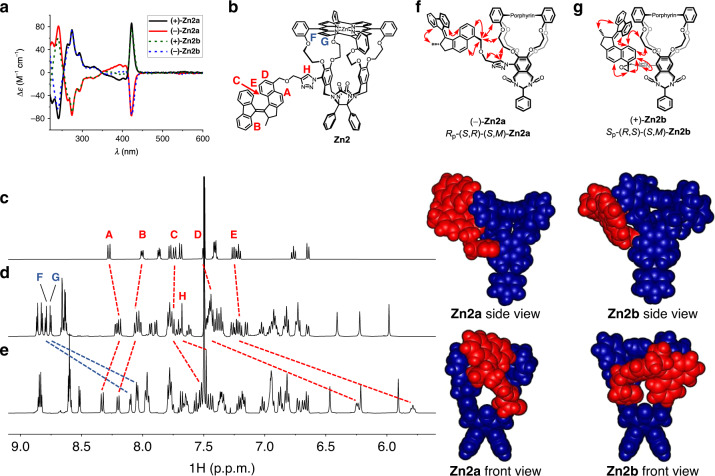
Characterization of motor compounds. **a** CD spectra of (+)-**Zn2a**, (−)-**Zn2a**, (+)-**Zn2b**, and (−)-**Zn2b** in CH_2_Cl_2_ (*c* = 3.9 × 10^−5^ M, 298 K). **b** Molecular structure of **Zn2**, with protons A–H indicated. **c**–**e** 500 MHz ^1^H NMR spectra of **8**, **Zn2a**, and **Zn2b**, respectively (*c* = 10^−3^ M in CDCl_3_/CD_3_CN, 1 : 1, v/v, 298 K), with the changes in chemical shift of motor protons A–E and H indicated in red and those of the macrocycle protons F–G in blue. **f**, **g** Key NOEs (red arrows) observed for the motor protons of **Zn2a** and **Zn2b**, and Spartan-calculated three-dimensional structures of (−)-*R*_p_-(*S*,*R*)-(*S*,*M*)-**Zn2a** and (+)-*S*_p_-(*R*,*S*)-(*S*,*M*)-**Zn2b**, based on the observed NOEs. Atoms of the molecular motor moiety and the macrocycle are indicated in red and blue, respectively.

To be able to connect **8** to a porphyrin cage compound via click chemistry, the latter needed to be equipped with an azide functionality. To this end, free base porphyrin cage compound **H**_**2**_**1**^26,27^ was regioselectively mononitrated at one of its xylylene sidewalls via our recently developed procedure^28^ to afford **H**_**2**_**9** (50–75%). This compound was subsequently reduced to monoamino cage **H**_**2**_**10** with stannous chloride and HCl^29^. The aniline derivative was readily transformed into azide **H**_**2**_**11** via the diazonium intermediate. Finally, a zinc(II) center was inserted into the porphyrin to protect it against copper insertion in the subsequent reaction step. Zinc azide cage **Zn11** was obtained from **H**_**2**_**9** in 90% yield over three steps. **Zn11** was then reacted with alkyne **8** in a copper(I)-mediated azide-alkyne cycloaddition to give triazole **Zn2** in 74% yield. Both cage compound **Zn11** and motor element **8** are chiral and are prepared as racemic mixtures. Hence, their interconnection resulted in a mixture of four compounds, i.e., two diastereomeric sets of enantiomers. The diastereomers (±)-**Zn2a** and (±)-**Zn2b** were separated using conventional 60H silica gel column chromatography and the resulting racemates were fully characterized by ^1^H NMR, ^13^C NMR, and two-dimensional (2D) NMR techniques, which clearly demonstrated the connectivity of the molecular motor and the porphyrin cage compound (Supplementary Figs. 3–8). Finally, the enantiomers could be separated on a Chiralpak ID (**Zn2a**) or Chiralpak IE (**Zn2b**) column. They were assigned (+) or (−) depending on whether the CD sign of the Soret band at 425 nm was positive or negative. The CD spectra of each of the four stereoisomers of **Zn2** clearly reveal the relative differences in stereochemistry of **Zn2a** and **Zn2b** (Fig. 3a). Whereas for (+)-**Zn2a** the characteristic Soret band of the porphyrin (*λ*_max_ = 425 nm) and the molecular motor absorption region (*λ* = 250–350 nm) have the same positive CD signatures, these signatures are opposite (Soret band positive and motor absorption region negative) in the case of (+)-**Zn2b**. Based on the CD spectra, we could assign the absolute configurations of the compounds in the following way. A zinc(II) cage with a single substituent on the cavity wall displaying a positive CD band at the Soret position has the absolute configuration *S*_p_-(*R*,*S*)^30^. The non-substituted variant of motor **2** with a negative CD band at ~300 nm has the absolute configuration (*S*,*M*)^31^. The descriptors *S*_p_-(*R*,*S*) and *R*_p_-(*S*,*R*) refer to the planar and two point chiralities of the cage compound and the descriptors (*R*,*P*) and (*S*,*M*) indicate the central and helical chirality of the motor substituent. This leads to the following assignments:

(+)-**Zn2a** (positive CD Soret band, positive motor CD band at 300 nm): *S*_p_-(*R*,*S*)-(*R*,*P*)

(−)-**Zn2a** (negative CD Soret band, negative motor CD band at 300 nm): *R*_p_-(*S*,*R*)-(*S*,*M*)

(+)-**Zn2b** (positive CD Soret band, negative motor CD band at 300 nm): *S*_p_-(*R*,*S*)-(*S*,*M*)

(−)-**Zn2b** (negative CD Soret band, positive motor CD band at 300 nm): *R*_p_-(*S*,*R*)-(*R*,*P*)

These assignments apply to the stable molecular motor-functionalized porphyrin macrocycles. For the irradiated unstable structures, the helicities revert from (*P*) to (*M*) and from (*M*) to (*P*), whereas the planar and point chiralities remain unchanged.

The remarkable ease by which the two different diastereomers **Zn2a** and **Zn2b** could be separated, based on their differences in polarity, indicates that their three-dimensional structures are significantly different. Figure 3c–e shows the aromatic region of the ^1^H NMR spectra of motor **8** (Fig. 3c) and of the conjugates **Zn2a** (Fig. 3d) and **Zn2b** (Fig. 3e), respectively. The shifts of the relevant proton signals A–E and H of the motor moieties are highlighted with dashed red lines and those of relevant macrocycle protons F and G with dashed blue lines. The signals of motor protons A–E in cage **Zn2a** do not shift much compared to the signals of motor **8**. In contrast, protons D and E of **Zn2b** are significantly shielded (>1 p.p.m.) compared to these protons in compounds **8** and **Zn2a**.

The shielding effect observed for proton E (~1.4 p.p.m.) was more pronounced in the more polar solvent mixture CDCl_3_/CD_3_CN (1 : 1, v/v) than in pure CDCl_3_ (~0.9 p.p.m.; Supplementary Fig. 5), suggesting that the effect is of solvophobic nature. Simultaneously, β-pyrrolic protons F and G, located above the entrance of the cavity at the face of the molecular motor are also significantly shielded in **Zn2b** when compared to compound **Zn2a**. Unique to **Zn2b** were 2D rotating frame Overhauser effect spectroscopy cross peaks, which revealed the close proximity of proton D and triazole proton H. In addition, protons C and E of the naphthalene part of the molecular motor showed NOE contacts with the protons of the ethylene glycol spacer connected to the sidewall of the macrocycle, opposite to the sidewall to which the motor is connected (see Fig. 3g). For compound **Zn2a**, on the contrary, NOE contacts were detected between the benzylic protons of the motor part and the ethylene glycol spacer and *meso*-phenyl ring attached to the same sidewall of the macrocycle to which the motor is connected (see Fig. 3f). Hence, it was concluded that **Zn2a** adopts a more open geometry in which the molecular motor is relatively free to move. In contrast, compound **Zn2b** adopts a more folded geometry, in which protons D and E point into the shielding zone of the cavity of the cage compound. All key NOEs observed between motor protons and macrocycle protons are indicated in Fig. 3f–g. To get more insight into the three-dimensional structures of **Zn2a** and **Zn2b**, molecular modeling studies (Spartan ‘14^TM^, PM3) were performed using the observed NOEs as starting points. The results are shown in Fig. 3f–g, which clearly indicate that in **Zn2b** the orientation of the motor part is such that the opening of the cage of the macrocycle is more blocked than in **Zn2a**. The naphthalene part of the motor in **Zn2b** is partially bound in the cavity of the macrocycle. The calculated parallel-displaced *π*–*π* stacking of the naphthalene unit and the porphyrin ring is in agreement with the observed shielding of motor protons D and E, and β-pyrrolic protons F and G. It may be concluded that the combination of the helicity of the overcrowded alkene in the motor part and the planar chirality of the macrocycle determine the interaction of the switch and macrocycle. Therefore, we expect that photochemical isomerization of the motor parts (inversion of the helicity) results in opposite behavior of **Zn2a** and **Zn2b**. Furthermore, we may expect that due to the different degrees of steric impediments by the motor parts in diastereomers **Zn2a** and **Zn2b**, the threading and binding properties of guest molecules in these macrocycles will be influenced (vide infra).

### Host–guest binding

**Fig. 4:**
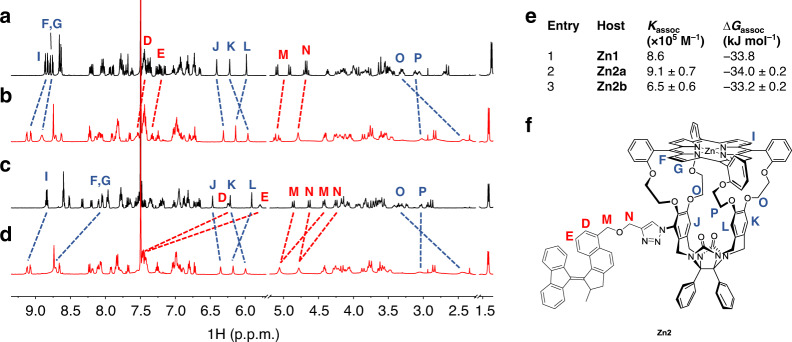
Characterization of host–guest complexes. **a**–**d** ^1^H NMR spectra of **a Zn2a**, **b Zn2a·V12**, **c Zn2b**, and **d Zn2b·V12** (500 MHz, *c* = 10^−3^ M in CDCl_3_/CD_3_CN, 1:1, v/v, 298 K). The shift changes of relevant cage protons are indicated in red and those of relevant motor protons in blue. **e** Association constants and Gibbs binding free energies of complexes between porphyrin macrocycles and methyl viologen bis(hexafluorophosphate) (**V12**) at 298 K in CHCl_3_/CH_3_CN, 1:1, v/v. **f** Molecular structure of **Zn2**, with protons D–G and I–P indicated.

Porphyrin macrocycles, such as **H**_**2**_**1** and **Zn1**, are known for their ability to strongly bind viologen guests (Fig. 2b)^27–30,32–34^. Due to the presence of the motor substituent at one of their side walls, the cavities of **Zn2a** and **Zn2b** are expected to be more sterically hindered than that of the parent porphyrin cage compound **Zn1**, as mentioned above. Nevertheless, both **Zn2a** and **Zn2b** were found to bind methyl viologen bis(hexafluorophosphate) (**V12**) with a similar strength as **Zn1** (Fig. 4e)^27^. ^1^H NMR studies revealed that upon binding of the guest, both **Zn2a** and **Zn2b** underwent significant structural changes (Fig. 4a–d). The signals of the ethylene glycol spacer protons O, located at the unsubstituted side of the cage compound, shifted upfield by 1 p.p.m., whereas the signal of the corresponding proton P, located at the substituted side of the cage compound, was unaffected. In addition, β-pyrrolic protons I located above the entrance of the unsubstituted side of the cavity became deshielded. For compound **Zn2b**, the β-pyrrolic protons F and G were also deshielded. The shielding effect observed for protons D and E in uncomplexed **Zn2b** was completely reversed, indicating the removal of the interactions between the cavity of the macrocycle and the attached motor moiety. The corresponding motor protons D and E of **Zn2a** were hardly affected by the binding of the viologen guest. Eventually, the NMR spectra of the 1:1 host–guest complexes **Zn2a·V12** and **Zn2b·V12** became nearly fully superimposable as a result of the disappearance of the through-space interactions between the macrocycle and the motor moiety in the latter complex. Although the proton signals of **V12** could not be observed due to rapid exchange on the NMR timescale, the spectral changes of the host were clearly indicative of the fact that the guest was bound in a non-symmetric mode. Apparently, for both **Zn2a** and **Zn2b**, the presence of a substituent on one side of the cage directs the viologen guest towards the other, unsubstituted side. Similar non-symmetrical binding geometries were observed for the 1:1 complexes of **Zn2a** and **Zn2b** with benzyl viologen bis(hexafluorophosphate) **V13** and cyclohexylmethyl viologen bis(hexafluorophosphate) **V14** (Supplementary Figs. 9–S10).

### Rotational behavior

**Fig. 5:**
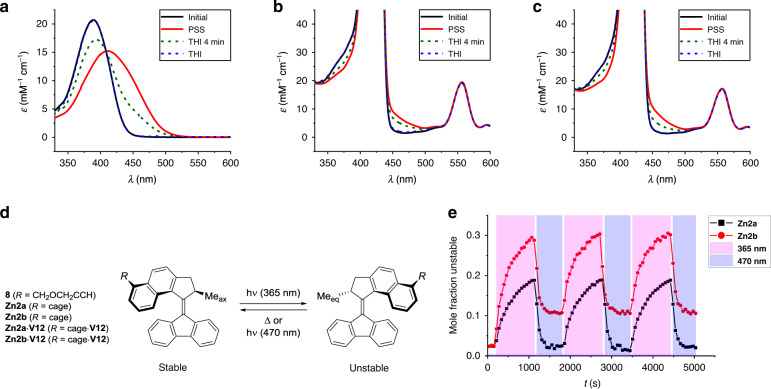
Spectroscopic features of motor conjugates and their complexes with viologen guests. **a–c** UV-vis absorption spectra of **a** motor **8**, **b Zn2a**, and **c Zn2b** before irradiation (blue), after irradiation (*λ* = 365 nm) to PSS (red), after partial thermal helix inversion (THI, green dashed line), and after full thermal helix inversion (blue dashed line) (*c* = 10^−5^ M in CHCl_3_/CH_3_CN, 1:1, v/v, 298 K). **d** Photochemical and thermal isomerization of motor compounds. **e** Photochemical switching of **Zn2a** and **Zn2b** by irradiating alternately with *λ* = 365 nm and *λ* = 470 nm. Mole fractions were determined by ^1^H NMR spectroscopy (*c* = 10^−3^ M in CDCl_3_/CD_3_CN, 1:1, v/v).

*UV-vis experiments*. As the binding of a viologen guest inside the cavity of cage compounds **Zn2a** and **Zn2b** clearly influences the three-dimensional geometry of the entire complex, it may also influence the rotary process of the motor substituent. To investigate this, we studied in a first series of experiments the photochemical and thermal isomerization steps of motors **8**, **Zn2a**, and **Zn2b** by ultraviolet–visible (UV-vis) absorption spectroscopy (Fig. 5). Upon irradiation (*λ* = 365 nm) to the photostationary state (PSS), the absorption maximum of **8** shifted from *λ* = 389 nm to *λ* = 411 nm (Fig. 5a). This spectral change is in agreement with spectral data for similar second-generation molecular motors^22^. Upon thermal relaxation, the original UV-vis spectrum was restored. Furthermore, an isosbestic point was found at *λ* = 408 nm, indicative of a unimolecular process. As a consequence of the very strong absorption of the Soret band of the porphyrin (*λ*_max_ = 425 nm), UV-vis absorption spectroscopy proved to be less effective for studying the photochemical behavior of **Zn2a** and **Zn2b**, as well as of their host–guest complexes with **V12**. Nevertheless, upon irradiation of solutions of **Zn2a** (Fig. 5b) or **Zn2b** (Fig. 5c), a decrease in absorption around *λ* = 380 nm was observed, alongside an increase in absorption around *λ* = 460 nm. This bathochromic shift matched with the aforementioned change observed for motor **8**. Again, after the thermal helix inversion, the original spectra were reobtained. Furthermore, for both **Zn2a** and **Zn2b**, isosbestic points were found at *λ* = 406 nm and at *λ* = 410 nm, respectively. Finally, the absorption of the main Q-band of the porphyrin in **Zn2a** and **Zn2b** (*λ*_max_ = 557 nm) did not change throughout the rotary cycle, indicating that the porphyrin moiety itself was stable under the irradiation conditions.

### Rotational behavior

*NMR experiments*. Subsequently, we studied the photochemical and thermal isomerization steps of the molecular motors by ^1^H NMR spectroscopy (Table 1). After extensive irradiation with UV-light (*λ* = 365 nm) to reach the PSS, distinctive changes were observed in the ^1^H NMR spectra of all investigated motor compounds, indicating that the stable motors were photoisomerized to the corresponding unstable intermediates. The characteristic signals of the motor methyl group (Me_ax_ at *δ* = 1.36–1.42 p.p.m.) shifted downfield (Me_eq_ at *δ* = 1.61–1.70 p.p.m.) as a result of a pseudo-axial to pseudo-equatorial change in orientation (Supplementary Figs. 11–13)^25^. The thermal helix inversion of the unstable intermediates back to the stable motor configurations was effectuated by removing the irradiation source. The resulting spectra perfectly reproduced the original thermal spectra and indicated the completion of the 180° rotary cycles. For each motor, the thermal relaxation process was measured at five different temperatures in order to determine the activation parameters for the thermal helix inversion (Supplementary Fig. 16–24). These parameters were extracted from the Eyring plots (Supplementary Table 3). We first determined the properties of free molecular motor **8** (Table 1, entry 1) and its values for the half-life at 20 °C (*t*_1/2_ = 6.5 min) and the PSS ratio (PSS_365_ = 79 : 21) were taken as reference values. Then, we investigated the influence of a porphyrin macrocycle (e.g., **Zn1**) on the characteristics of motor **8** (Table 1, entry 2). We found that a 1:1 mixture of **8** and **Zn1** displayed a similar *t*_1/2_ as free molecular motor **8**, but a substantially lower PSS ratio. This lower PSS ratio can be attributed to the fluorescent properties of **Zn1** (Supplementary Fig. 26). Upon irradiating **Zn1** with *λ* = 365 nm, three fluorescence emission maxima were observed, centered at 437, 607, and 658 nm. The band at 437 nm overlaps with the absorption band of unstable-**8** (Fig. 5a). Thus, a possible explanation for the lower PSS ratio of motor **8** in the presence of **Zn1** is that the photons (*λ* = 437 nm) emitted by **Zn1** excite unstable-**8** and thereby promote the reverse photochemical reaction of unstable-**8** back to stable-**8**. The addition of excess viologen **V12** (2.0 equiv.) to the 1:1 mixture of **8** and **Zn1** did not affect the half-life of unstable-**8** (Table 1, entry 3). However, it resulted in a higher PSS ratio. In line with our previously reported experiments^17^, the viologen guest was shown to quench the fluorescence of **Zn1**, thereby removing the possibility to back-excite unstable-**8**. As a control experiment, we also tested a 1:1 mixture of motor **8** and the non-luminescent and paramagnetic cage compound **Mn1**^16^ (Table 1, entry 4). It is evident that the function of the molecular motor is compatible with a manganese(III) porphyrin macrocycle, i.e., the half-life of unstable-**8** is not influenced by the manganese(III) porphyrin. This observation is important in the sense that a manganese(III) derivative of **Zn2** will be used as photo-switchable catalyst in which the motor moiety acts as the switching unit. In a last control experiment, we examined the properties of motor **8** in the presence of the non-luminescent and diamagnetic compound **Ni1**^26^ (Table 1, entry 5). Both the *t*_1/2_ values and the PSS ratio were now found to be in the same range as those observed for motor **8**, which supports the aforementioned back-excitation effect displayed by luminescent **Zn1**.

**Table 1:** Photochemical and thermal properties of motor compounds.

Entry	Motor	Additive	Guest	ΔG^‡^ (20 °C) (kJ mol^−1^)	*t*_1/2_ (20 °C) (min)	PSS_365_ (4 °C) unstable: stable
1	**8**	—	—	87.2 ± 0.8	6.5	79 : 21
2	**8**	**Zn1** (1 eq.)	—		6.5	39 : 61
3	**8**	**Zn1** (1 eq.)	**V12** (2 eq.)		6.3	66 : 34
4	**8**	**Mn1** (1 eq.)	—		6.5	58 : 42
5	**8**	**Ni1** (1 eq.)	—		6.5	67 : 33
6	**Zn2a**	—	—	88.6 ± 0.9	11.6	24 : 76
7	**Zn2a**	—	**V12** (2 eq.)	87.1 ± 1.2	6.3	60 : 40
8	**Zn2b**	—	–	86.7 ± 0.7	5.3	37 : 63
9	**Zn2b**	—	**V12** (2 eq.)	86.8 ± 0.6	5.7	64 : 36

Selected kinetic and thermodynamic parameters (at 293 K) for the thermal helix inversions of **8**, **Zn2a**, **Zn2b**, and their host–guest complexes with **V12**, and photostationary state ratios (PSSs, measured at 277 K) for all the motor compounds (*c* = 10^−3^ M in CDCl_3_/CD_3_CN, 1:1, v/v).

In a subsequent series of experiments, we studied the rotational properties of the conjugate systems **Zn2a** and **Zn2b**, as well as their host–guest complexes **Zn2a·V12** and **Zn2b·V12** (Table 1, entries 6–9). Compared to free motor **8**, the motor substituent of **Zn2a** displayed a doubled *t*_1/2_ of 12 min at 20 °C, which indicates stabilization of unstable-**Zn2a** (Table 1, entry 6). ^1^H NMR studies revealed that upon photoisomerization three-dimensional structural changes occurred for this compound, in the sense that unstable-**Zn2a** displayed similar shielding effects as those that were observed for protons D and E of stable-**Zn2b** (Supplementary Fig. 14). Although unstable-**Zn2a** and stable-**Zn2b** differ in the point chirality at the stereogenic center of the molecular motor, their relative planar and helical chiralities are identical (either *R*_p_-(*P*) or *S*_p_-(*M*)). Hence, we reason that the *π*–*π* stacking interactions between the naphthalene part of the motor and the porphyrin in unstable-**Zn2a** and stable-**Zn2b** are similar. This supramolecular interaction is considered to impede the movement of the naphthalene part of the motor with respect to the fluorene stator of the motor. As a result, the thermal helix inversion of unstable-**Zn2a** to stable-**Zn2a** is slower than the corresponding process of the other second-generation molecular motors lacking this additional interaction in the unstable state. Upon irradiation of **Zn2a** with *λ* = 365 nm, we observed a fluorescence emission maximum centered at *λ* = 438 nm (Supplementary Fig. 28). In line with what we found for motor **8** in the presence of **Zn1**, this fluorescence caused a low PSS ratio for **Zn2a**. The addition of guest **V12** to conjugate system **Zn2a** resulted in a higher rotational speed (Table 1, entry 7), which can be attributed to the absence of the stacking interactions between the porphyrin macrocycle and the molecular motor when a viologen guest is bound inside the cavity. Furthermore, **V12** again quenched the fluorescence of the zinc porphyrin and thereby increased the PSS ratio of **Zn2a**.

Finally, we investigated the conjugate system **Zn2b**, which displayed through-space interactions in the stable configuration (Table 1, entry 8). We expected the three-dimensional structure of unstable-**Zn2b** to be similar to stable-**Zn2a**, based on their identical relative planar and helical stereochemistries. Indeed, ^1^H NMR analysis indicated that the interaction between the naphthalene part of the motor and the macrocycle was absent in unstable-**Zn2b** (Supplementary Fig. 15). The half-life of unstable-**Zn2b** was found to be similar to that of free molecular motor **8**. In addition, the PSS ratio of **Zn2b** was similar to that of the 1:1 mixture of motor **8** and porphyrin cage compound **Zn1**, which again may be attributed to the fluorescent properties of the zinc porphyrin (Supplementary Fig. 29, *λ*_em_ = 439 nm). As unstable-**Zn2b** did not experience a stabilization effect, the addition of viologen guest **V12** was not expected to influence the half-life of the motor moiety of **Zn2b**. Our experiments showed this indeed to be the case (Table 1, entry 9). Nevertheless, **V12** again quenched the fluorescence of the zinc porphyrin and, as a result, a higher PSS ratio was obtained. Thus, the binding of a viologen guest can influence both the thermal and photochemical properties of the conjugate systems. In this context, it is of interest to mention that host–guest interactions in crown-ether functionalized second-generation light-driven molecular motors have been used to modulate the properties of the molecular motor moiety^35^.

### Light switchability

The bathochromic shift observed upon the photoisomerization of **Zn2a** and **Zn2b** might be exploited to deliberately photoexcite the unstable intermediates and regenerate the stable isomers. By irradiating a solution of a molecular motor at *λ* = 470 nm, the unstable isomer of a motor species might be able to absorb light at this wavelength, while both the porphyrin and the stable isomer of the motor should not be able to absorb to the same extent. In this manner, we would be able to construct an orthogonally photo-switchable system (Fig. 5e). To investigate this, ^1^H NMR irradiation experiments were carried out in which solutions containing either **Zn2a** or **Zn2b** (*c* = 10^−3^ M in CDCl_3_/CD_3_CN, 1:1, v/v) were cooled to −10 °C, to minimize the extent to which thermal helix inversion could take place in the time regime of the experiment (**Zn2a**: *t*_1/2,263K_ ~ 9 h; **Zn2b**: *t*_1/2,263K_ ~ 5 h). Further cooling might completely eliminate the thermal processes, but is not recommended, because it may lead to aggregation of the porphyrin cage compounds. This competitive aggregation of the zinc porphyrin cage compounds at low temperature made −10 °C the best compromise. Our experiments demonstrated that both cage compounds **Zn2a** and **Zn2b** were repeatedly photo-switchable, although unstable-**Zn2b** could not be entirely isomerized back to stable-**Zn2b**. When a solution containing exclusively stable-**Zn2b** was irradiated at *λ* = 470 nm, substantial conversion into unstable-**Zn2b** was observed and the PSS_470_ ratio was found to be similar to the ratio depicted in Fig. 5e (1:9, unstable:stable). The *λ* = 470 nm light source used for these experiments emits light in the *λ* = 430–520 nm range with a maximum emission intensity at *λ* = 470 nm. A possible explanation for the fact that stable-**Zn2b**, but not stable-**Zn2a**, could be isomerized to the unstable state with this light source is the subtle difference in the UV-vis absorption spectra between the two compounds (Supplementary Fig. 27). In the entire wavelength region between *λ* = 430 nm and *λ* = 460 nm, the molar absorptivity of stable-**Zn2b** was found to be higher than that of stable-**Zn2a**, which is presumably caused by the interactions of motor and macrocycle in **Zn2b**, as depicted in Fig. 3g.

### Threading

**Fig. 6:**
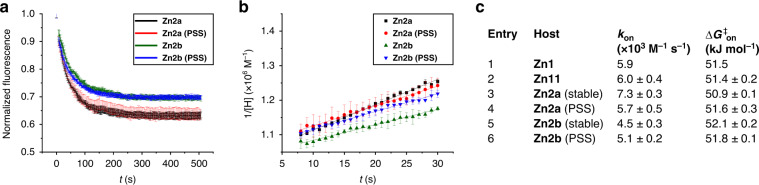
Threading experiments. **a** Normalized fluorescence intensity of the host measured as a function of time, after the addition of 1.0 equivalent of **V15** ([host]_0_ = [**V15**]_0_ = 10^−6^ M in CHCl_3_/CH_3_CN, 1:1, v/v). **b** Second-order kinetics plots for the complexation of hosts **Zn2a** and **Zn2b**, and their PSS mixtures with **V15** for the initial stages (~first 50%) of the threading process. **c** Kinetic and thermodynamic data for the threading of porphyrin macrocycles onto polymer guest **V15**. Data were acquired by fluorescence spectroscopy at 298 K (*c* = 10^−6^ M in CHCl_3_/CH_3_CN, 1:1, v/v).

Having established the light-driven rotary motion and light-switchable properties of **Zn2a** and **Zn2b**, we decided to investigate the effect of the (state of the) motor substituent on the threading of the cage compounds onto a polymeric guest. Small molecular weight viologens of the type **V12**–**V14** bind rapidly inside the cavity of porphyrin cage compounds, such as **H**_**2**_**1** and **Zn1**, making it impossible to follow the binding process over time using conventional NMR or optical techniques^32–34^. We have previously shown that the binding of one-side blocked viologens appended with polymer chains can take up to minutes at micromolar concentrations, because the porphyrin cage compound has to find the open end of the polymer and traverse the entire polymer chain before it can reach the viologen binding station^32–34^. This threading process can be studied with fluorescence quenching spectroscopy^32^. To investigate to what extent the presence of a molecular motor functionality close to the opening of the cage compound would influence the threading properties, we studied the threading of polymer **V15** through **Zn2a** and **Zn2b** (both in their stable and unstable configurations). To this end, we mixed equimolar amounts of host and guest (*c* = 10^−6^ M in CHCl_3_/CH_3_CN, 1:1, v/v), and monitored the quenching of the fluorescence of the host by the guest as a function of time (Fig. 6a)^32^. To avoid any in situ excitation of the molecular motor substituents, the zinc porphyrins were excited at one of their Q-bands (*λ* = 550 nm). In line with what we reported earlier for **Zn1**, the kinetics of the fluorescence quenching followed a second order process^32–34^. The plots of 1/[host] as a function of time displayed a linear time dependency for the initial stages of the threading process (Fig. 6b) and the threading rates *k*_on_ could be determined from the slope of the linear fits (Fig. 6c). From the obtained data, it is evident that both molecular motor-substituted hosts **Zn2a** and **Zn2b** can find the end of a polymer, thread on it, and move along the chain to eventually bind at the viologen trap. The mechanism of this process most likely is the same as the one we reported before for the unsubstituted porphyrin cage compounds **H**_**2**_**1** and **Zn1**^34^. The *k*_on_-values of **Zn2a** and **Zn2b** were found to be in the same order of magnitude as those of the non-substituted host **Zn1**^34^ (Fig. 6c, entry 1) and azide-substituted host **Zn11** (entry 2, Supplementary Fig. 30). The *k*_on_-value of stable-**Zn2a** (entry 3) was ~1.6-fold higher than that of stable-**Zn2b** (entry 5), which is in line with the observation that the former host compound has a more open entrance than the latter host compound, allowing easier access of the polymer chain. Then, we investigated the effect of first irradiating the solutions of the motor–cage conjugates, allowing them to reach the PSS and subsequently mixing them with the polymeric guest. The irradiation of stable-**Zn2a** (larger cavity opening) to unstable-**Zn2a** (smaller cavity opening) resulted in slower threading (compare entries 3 and 4). In contrast, the irradiation of stable-**Zn2b** (smaller cavity opening) to unstable-**Zn2b** (larger cavity opening) resulted in faster threading (compare entries 5 and 6). Although the PSS ratios for the isomerization of both diastereomers of **Zn2** are low and the observed effects on the threading rates in the current design are as yet relatively small, it is evident that the rate is dependent on the (switching) state of the motor component. Our explanation for these effects is that in a pre-equilibrium the motor component competes with the polymer chain end for binding in the cavity of the macrocycle (entron effect)^34^, resulting in a slower or faster threading. The extent to which this competitive binding takes place is dependent on the relative planar and helical chiralities of the macrocycle and the motor, respectively. As the former stereochemical element is fixed, the latter can be switched, allowing dynamic control of the threading process. We hope to improve this dynamic selectivity of the threading process by designing improved motor-porphyrin macrocycles, i.e., compounds that possess a rigid linker between the motor part and the porphyrin macrocycle.

Polymer **V15** can thread through macrocycles **Zn2a** and **Zn2b** in two ways, i.e., with the polymer entering the cavity from the unsubstituted site or from the site to which the motor is attached. This directionality issue has been studied in detail in the case of low-molecular-weight guests^36^, but not yet in the case of polymeric ones. For future experiments, however, we intend to use C_2_-symmetric porphyrin macrocycles that contain two diagonally placed motor elements, resolving the ambiguity that exist with the current system.

## Discussion

We have shown that porphyrin macrocyclic compounds, which are aimed to be used as photo-switchable catalytic machines for the writing of digital (chemical) information on synthetic polymer chains, can be provided with a motor element via a convenient synthesis protocol. The prepared compounds (**Zn2a** and **Zn2b**) are diastereomers and display the characteristic features of both the motor and the porphyrin macrocycle. These two diastereomers and the two corresponding enantiomers could be separated and were fully characterized. ^1^H NMR studies revealed that **Zn2a** and **Zn2b** adopt intrinsically different three-dimensional geometries, with **Zn2a** having a more open structure than **Zn2b**. This difference can be partly alleviated by the addition of viologen guest molecules. We have also shown that both **Zn2a** and **Zn2b** can function as molecular motors. The thermal isomerization step of **Zn2a** is slower than that of the other investigated motor, due to stabilization of the intermediate, i.e., unstable-**Zn2a**. The addition of viologen guests affects both the photochemical and thermal isomerization steps of **Zn2a** and **Zn2b**, resulting in PSS ratios that are enriched in the unstable isomer. Moreover, the addition of the viologen guest resulted in an enhanced rotational speed for **Zn2a**. Furthermore, our studies reveal that the motorized cage compounds can be switched repeatedly in an orthogonal fashion, rendering them useful for future light-switchable catalytic writing in the form of enantiomeric epoxide functions by manganese (III) modified porphyrin macrocycles. For the latter purpose, the PSS ratios still need to be improved further, i.e., a higher population of unstable isomers is required. Finally, we have demonstrated that the state of the molecular motor influences the steric encumbrance around the cavity entrance of the cage compound, resulting in different threading rates for the diastereomeric macrocycles **Zn2a** and **Zn2b**. So far, the difference in the *k*_on_ values between stable configurations of the two compounds is only a factor 1.6. We hope to improve this selectivity by synthesizing molecular motors that are more rigidly connected to the functionalized cage compounds, to compare them with the more flexible motor-porphyrin macrocycles described in this study. The best systems will eventually be chosen as switchable catalysts for the catalytic writing on polymer chains.

## Methods

Free base porphyrin macrocycle **H**_**2**_**1**, zinc(II) porphyrin macrocycle **Zn1**, manganese(III) porphyrin macrocycle **Mn1**, nickel(II) porphyrin macrocycle **Ni1**, 9-diazofluorene, ketone **3**, and mononitro-macrocycle **H**_**2**_**9** were synthesized according to literature procedures.

*(±)-6-Bromo-2-methyl-2,3-dihydro-1H-cyclopenta[a]naphthalene-1-thione (***4***).* A suspension of ketone **3** (0.37 g, 1.3 mmol, 1.0 equiv.) and Lawesson’s reagent (0.60 g, 1.5 mmol, 1.1 equiv.) in toluene (15 mL) was refluxed for 2.5 h under an argon atmosphere. Then, the solvent was removed in vacuo and the residue was purified by silica gel column chromatography (eluent: dichloromethane (DCM)/heptane, 2:3, v/v) to afford impure thioketone **4** (0.28 g, 72%) as a dark green oil. Due to instability of the compound, it was immediately used in the next step without further purification.

*(±)-9-(6-Bromo-2-methyl-2,3-dihydro-1H-cyclopenta[a]naphthalen-1-ylidene)-9H-fluorene (***5***).* A solution of thioketone **4** (0.27 g, 0.93 mmol, 1.0 equiv.) and 9-diazofluorene (0.39 g, 2.0 mmol, 2.2 equiv.) in toluene (50 mL) was refluxed for 19 h under an argon atmosphere. Then, the mixture was evaporated to dryness. The residue was purified by silica gel column chromatography (eluent: heptane) to afford a mixture of motor **5** and 9,9’-bifluorenylidene (0.45 g in total, yield not determined) as an orange solid. The mixture containing motor **5** was immediately used in the next step without further purification.

*(±)-1-(9H-Fluoren-9-ylidene)-2-methyl-2,3-dihydro-1H-cyclopenta[a]naphthalene-6-carbaldehyde (***6***).* ^*n*^Butyllithium (1.0 mL, 1.6 mmol, 1.7 equiv., 1.6 M in hexanes) was added at −78 °C to a solution of motor **5** (0.45 g of the mixture, 0.93 mmol in theory, 1.0 equiv.) in dry tetrahydrofuran (THF) (10 mL) under an argon atmosphere. The resulting mixture was stirred at the same temperature for 1 h. Then, dry DMF (0.83 mL, 11 mmol, 11 equiv.) was added. The mixture was slowly warmed to 20 °C and stirred for 2 h. Thereafter, the reaction was quenched with aqueous 2 M NH_4_Cl (50 mL) and the product was extracted with EtOAc (2 × 30 mL). The combined organic extracts were washed with water (2 × 50 mL) and brine (2 × 50 mL), then dried over sodium sulfate and the solvent was removed in vacuo. The residue was purified by silica gel column chromatography (eluent: DCM/heptane, 1 : 3, v/v) to afford aldehyde **6** (0.16 g, 45% from thioketone **4**) as a yellow solid.

*(±)-(1-(9H-Fluoren-9-ylidene)-2-methyl-2,3-dihydro-1H-cyclopenta[a]naphthalen-6-yl)methanol (***7***)*. Sodium borohydride (15.2 mg, 0.40 mmol, 1.0 equiv.) was added to a solution of aldehyde **6** (150 mg, 0.40 mmol, 1.0 equiv.) in DCM/MeOH (1:1, v/v, 10 mL). The resulting mixture was stirred at 20 °C for 15 min. Upon completion (indicated by TLC, eluent: DCM), aqueous 2 M NH_4_Cl (40 mL) was added and the product was extracted with DCM (3 × 20 mL). The combined organic extracts were dried over sodium sulfate and the solvent was removed in vacuo to afford alcohol **7** (150 mg, 99%) as a yellow solid.

(±)*-9-(2-Methyl-6-((prop-2-yn-1-yloxy)methyl)-2,3-dihydro-1H-cyclopenta[a]naphthalen-1-ylidene)-9H-fluorene (***8***)*. A solution of alcohol **7** (105 mg, 0.28 mmol, 1.0 equiv.) in dry THF (5 mL) was added at 0 °C to a suspension of sodium hydride (17 mg, 0.42 mmol, 1.5 equiv., 60% in mineral oil) in dry THF (2 mL) under an argon atmosphere. The mixture was stirred at 0 °C for 70 min and then propargyl bromide (0.31 mL, 2.8 mmol, 10 equiv., 80% in toluene) was added. The resulting mixture was stirred at 20 °C for 21 h. Upon completion (indicated by thin layer chromatography (TLC), eluent: DCM), the reaction was quenched with water (40 mL) and the product was extracted with DCM (3 × 30 mL). The combined organic extracts were dried over sodium sulfate and the solvent was removed in vacuo. The residue was purified by silica gel column chromatography (eluent: DCM/pentane, 1:1, v/v) to afford alkyne **8** (104 mg, 90%) as a yellow solid.

*(±)-Zinc(II) azide cage* **(Zn11)**. A Schlenk bomb was charged with tin(II) chloride (1.5 g, 8.0 mmol, 40 equiv.) and mononitro cage **H**_**2**_**9** (0.28 g, 0.20 mmol, 1.0 equiv.). HCl (4 M) in dioxane (7 mL) and aqueous 37% HCl (1 drop) were successively added, the bomb was closed, and the mixture was vigorously stirred at 60 °C for 5 h. Upon completion (indicated by TLC, eluent: chloroform/acetonitrile, 9:1, v/v), the reaction was quenched with aqueous 3 M NaOH (60 mL) and the product was extracted with chloroform (2 × 50 mL). The combined organic extracts were successively washed with aqueous 1 M NaOH (50 mL), then dried over sodium sulfate and the solvent was removed in vacuo to afford crude amine **H**_**2**_**10** as a purple solid. The solid material was dissolved in chloroform/acetonitrile (60 mL, 3:1, v/v). ^*t*^BuONO (0.16 mL, 1.4 mmol, 7.0 equiv.) and TMSN_3_ (0.16 mL, 1.2 mmol, 6.0 equiv.) were added, and the mixture was stirred at 20 °C for 1.5 h. Upon completion (indicated by TLC, eluent: chloroform/acetonitrile, 9:1, v/v), the mixture was evaporated to dryness. The residue was purified by Alumina III column chromatography (eluent CHCl_3_) to afford azide cage **H**_**2**_**11** as a purple solid. The latter solid was dissolved in chloroform/methanol (75 mL, 2:1, v/v), zinc acetate dihydrate (0.28 g, 1.5 mmol, 7.5 equiv.) was added, and the resulting mixture was stirred at 20 °C for 20 h. Upon completion (indicated by MALDI-TOF), the mixture was evaporated to dryness. The residue was purified by silica gel column chromatography (eluent: chloroform/methanol, 99:1, v/v). Subsequently, the purified material was dissolved in a minimal amount of DCM and precipitated by the addition of heptane. Most of the DCM was removed under reduced pressure and the resulting suspension was centrifuged. The supernatant was removed and the precipitate was washed with pentane and dried under high vacuum to afford zinc azide porphyrin cage compound **Zn11** (0.26 g, 90% over 3 steps from **H**_**2**_**9**) as a purple solid.

### Zinc(II) cage molecular motor hybrid (Zn2)

Sodium ascorbate (164 mg, 0.83 mmol, 10 equiv.), copper(I) iodide (236 mg, 1.24 mmol, 15 equiv.) and *N,N*-diisopropylethylamine (DIPEA) (0.14 mL, 0.83 mmol, 10 equiv.) were successively added to a solution of **Zn11** (120 mg, 83 μmol, 1.0 equiv.) and alkyne motor **8** (51 mg, 124 μmol, 1.5 equiv.) in a degassed mixture of dimethyl sulfoxide (50 mL) and water (3 mL). The resulting mixture was stirred at 20 °C for 3 days under an argon atmosphere. Meanwhile, after 2 days, additional alkyne motor **8** (9 mg, 20 μmol, 0.25 equiv.) and copper(I) iodide (40 mg, 0.2 mmol, 2.5 equiv.) were added. Upon completion (indicated by TLC, eluent: chloroform/acetonitrile, 9:1, v/v), the mixture was diluted with water (150 mL) and the product was extracted with chloroform (3 × 50 mL). The combined organic extracts were washed with water (3 × 100 mL) and brine (100 mL), then dried over sodium sulfate and the solvent was removed in vacuo. The residue was purified by column chromatography (60H silica gel, eluent: chloroform/acetonitrile, 10:1, v/v). Excess alkyne motor **8** (29.0 mg) was recovered as a yellow solid. The first eluted pair of enantiomers (**Zn2a**, 58.3 mg, 38%) was isolated as a purple solid after precipitation from DCM/heptane (1:1 v/v). The second eluted pair of enantiomers (**Zn2b**, 55.0 mg, 36%) was also obtained as a purple solid after precipitation from the same solvent mixture. Samples of **Zn2a** or **Zn2b** were dissolved in dichloromethane and separated into enantiomers by injection on a Chiralpak ID or Chiralpak IE column, using ethanol/dichloromethane (30:70, v/v) as the mobile phase. The flow rate was 1 mL/min and detection was performed with a UV detector at *λ* = 420 nm.

### Additional information

Detailed synthetic procedures, resolution, and characterization of the compounds, UV-vis, fluorescence and NMR measurements, host–guest binding studies, and copies of NMR spectra of all new compounds are included in the Supplementary Information, which is available in the online version of the paper.

## Supplementary information

Supplementary Information

## Data Availability

The authors declare that the data supporting the findings of this study are included in the paper and its Supplementary Information. Any further relevant data are available from the corresponding authors on request.
