# Supplementary material for: Molecular motor-functionalized porphyrin macrocycles
Source: Nat Commun. 2020 Oct 20;11:5291. doi: 10.1038/s41467-020-19123-y (PMC7576194; doi:10.1038/s41467-020-19123-y)
Supplement: Supplementary file 1 — Supplementary Information [file 41467_2020_19123_MOESM1_ESM.pdf]

Supplementary Information

Molecular motor-functionalized porphyrin macrocycles

Pieter J. Gilissen, et al.

Supplementary Methods

General information

Free base porphyrin macrocycle **$H_2$1**,[1] zinc(II) porphyrin macrocycle **Zn1**,[2] manganese(III) porphyrin macrocycle **Mn1**,[3] nickel(II) porphyrin macrocycle **Ni1**,[1] 9-diazofluorene,[4] ketone **3**,[5,6] and mononitro-macrocycle **$H_2$9**[7] were synthesized according to literature procedures. Dichloromethane was distilled from calcium hydride under a nitrogen atmosphere. Tetrahydrofuran was distilled from potassium under a nitrogen atmosphere. Toluene was distilled from sodium under a nitrogen atmosphere. Chloroform was distilled from phosphorus pentoxide under a nitrogen atmosphere, and subsequently filtered through anhydrous potassium carbonate and purged with argon before use. Acetonitrile was distilled from calcium chloride under an argon atmosphere and purged with argon before use. Deuterated chloroform and acetonitrile were dried over molecular sieves (4Å) and purged with argon before use. Other solvents and reagents were obtained from commercial suppliers and used without further purification. Reactions were followed by using MALDI-TOF or by using thin-layer chromatography (TLC) on silica gel-coated plates (Merck 60 F254). Detection was performed with UV light at 254 nm and/or by charring at 150 °C after dipping in an aqueous solution of potassium permanganate. Column chromatography was performed manually using Acros silica gel, 0.035−0.070 mm, 60A, Merck silica gel, 60H, and Acros aluminium oxide, 0.050–0.200 mm, 60A. Melting points were taken on a polarization microscope with a programmable hot-stage. NMR spectra were recorded at 298 K (unless stated otherwise) on a Bruker Avance III 500 spectrometer (500 MHz) equipped with a Prodigy BB cryoprobe. ^{1}H NMR chemical shifts (δ) are given in parts per million (ppm) and were referenced to tetramethylsilane (0.00 ppm). Coupling constants are reported as *J* values in Hertz (Hz). Data for ^{1}H NMR spectra are reported as follows: chemical shift (multiplicity, coupling constant, integration, assignment if applicable). Multiplicities are abbreviated as s (singlet), d (doublet), t (triplet), q (quartet), p (quintet), m (multiplet), b (broad). Mass spectra were recorded on a JEOL AccuTOF CS JMS-T100CS mass spectrometer, on a JEOL TMS-100GCv, and on a Bruker Microflex LRF MALDI-TOF system in reflective or linear mode employing dithranol as a matrix. Chiral HPLC separations were conducted on a Chiralpak ID or Chiralpak IE column. UV-vis spectra were recorded at 298 K on a JASCO V-630 UV-vis spectrophotometer (1 cm quartz cell). Fluorescence spectra were recorded at 293 K on a JASCO FP-8300ST spectrofluorometer (1 cm quartz cell). ECD spectra were recorded at 298 K on a JASCO J-815 CD spectrophotometer (1 mm quartz cell). Irradiation experiments were carried out using Thorlabs Fiber-Coupled LEDs (M365FP1 and M470F3). Molecular models were compiled using the Spartan '14™ chemistry software (equilibrium geometry, PM3, semi-empirical method, gas phase), thereby taking into account distance constraints that were derived from the observed NOE contacts in the 2D ROESY experiments of the compounds.

Experimental Details

(±)-6-Bromo-2-methyl-2,3-dihydro-1*H*-cyclopenta[*a*]naphthalene-1-thione (4)

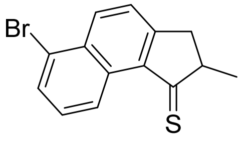

(±)-**4**

A suspension of ketone **3** (0.37 g, 1.3 mmol, 1.0 equiv) and Lawesson's reagent (0.60 g, 1.5 mmol, 1.1 equiv) in toluene (15 mL) was refluxed for 2.5 hours under an argon atmosphere. Then, the solvent was removed in vacuo and the residue was purified by silica gel column chromatography (eluent: DCM/heptane, 2:3, v/v) to afford impure thioketone **4** (0.28 g, 72%) as a dark green oil. Due to instability of the compound, it was immediately used in the next step without further purification. ^{1}H NMR (500 MHz, $CDCl_3$) δ 10.14 (d, *J* = 8.6 Hz, 1H), 8.56 (d, *J* = 8.7 Hz, 1H), 7.85 (dd, *J* = 7.5, 1.1 Hz, 1H), 7.64 (d, *J* = 8.7 Hz, 1H), 7.54 (dd, *J* = 8.6, 7.5 Hz, 1H), 3.54 (dd, *J* = 18.1, 6.6 Hz, 1H), 3.18 (pd, *J* = 7.1, 2.3 Hz, 1H), 2.94 (dd, *J* = 18.0, 2.5 Hz, 1H), 1.51 (d, *J* = 7.2 Hz, 3H).

(±)-9-(6-Bromo-2-methyl-2,3-dihydro-1*H*-cyclopenta[*a*]naphthalen-1-ylidene)-9*H*-fluorene (5)[8]

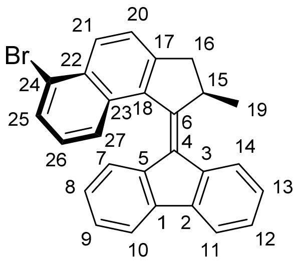

(±)-**5**

A solution of thioketone **4** (0.27 g, 0.93 mmol, 1.0 equiv) and 9-diazofluorene (0.39 g, 2.0 mmol, 2.2 equiv) in toluene (50 mL) was refluxed for 19 hours under an argon atmosphere. Then, the mixture was evaporated to dryness. The residue was purified by silica gel column chromatography (eluent: heptane) to afford a mixture of motor **5** and 9,9'-bifluorenylidene (0.45 g in total, yield not determined) as an orange solid. The mixture containing motor **5** was immediately used in the next step without further purification. ^{1}H NMR (500 MHz, $CDCl_3$) δ 8.37 (d, *J* = 8.5 Hz, 1H, 21-C*H*), 7.99–7.95 (m, 1H, 14-C*H*), 7.86–7.82 (m, 1H, 11-C*H*), 7.79 (d, *J* = 8.5 Hz, 1H, 27-C*H*), 7.78–7.72 (m, 2H, 10-C*H* + 25-C*H*), 7.68 (d, *J* = 8.2 Hz, 1H, 20-C*H*), 7.43–7.37 (m, 2H, 12-C*H* + 13-C*H*), 7.22 (td, *J* = 7.4, 1.0 Hz, 1H, 9-C*H*), 7.14 (dd, *J* = 8.5, 7.4 Hz, 1H, 26-C*H*), 6.81 (td, *J* = 7.7, 1.2 Hz, 1H, 8-C*H*), 6.66 (d, *J* = 8.0 Hz, 1H, 7-C*H*), 4.37 (p, *J* = 6.4 Hz, 1H, 15-C*H*), 3.60 (dd, *J* = 15.4, 5.8 Hz, 1H, 16-CH_a), 2.78 (d, *J* = 15.2 Hz, 1H, 16-CH_b), 1.38 (d, *J* = 6.8 Hz, 3H, 19-CH_3); ^{13}C NMR (126 MHz, $CDCl_3$) δ 150.37, 148.20, 141.14, 140.32, 139.86, 139.80, 137.15, 137.07, 131.27, 131.24, 130.00, 129.71, 127.56, 127.33, 127.18, 127.03, 126.21, 125.73, 125.49, 124.29, 123.60, 119.87, 119.20, 45.81, 41.96, 19.31.

(±)-1-(9*H*-Fluoren-9-ylidene)-2-methyl-2,3-dihydro-1*H*-cyclopenta[*a*]naphthalene-6-carbaldehyde (6)

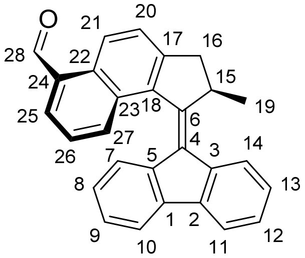

(±)-**6**

^{n}Butyllithium (1.0 mL, 1.6 mmol, 1.7 equiv, 1.6M in hexanes) was added at −78 °C to a solution of motor **5** (0.45 g of the mixture, 0.93 mmol in theory, 1.0 equiv) in dry THF (10 mL) under an argon atmosphere. The resulting mixture was stirred at the same temperature for 1 hour. Then, dry DMF (0.83 mL, 11 mmol, 11 equiv) was added. The mixture was slowly warmed to 20 °C and stirred for 2 hours. Thereafter, the reaction was quenched with aqueous 2M NH_4Cl (50 mL) and the product was extracted with EtOAc (2 × 30 mL). The combined organic extracts were washed with water (2 × 50 mL) and brine (2 × 50 mL); then dried over sodium sulfate and the solvent was removed in vacuo. The residue was purified by silica gel column chromatography (eluent: DCM/heptane, 1:3, v/v) to afford aldehyde **6** (0.16 g, 45% from thioketone **4**) as a yellow solid. m.p. 227–229 (dec.); ^{1}H NMR (500 MHz, $CDCl_3$) δ 10.44 (s, 1H, 28-C*H*), 9.38 (d, *J* = 8.6 Hz, 1H, 21-C*H*), 8.15 (d, *J* = 8.5 Hz, 1H, 27-C*H*), 8.01–7.97 (m, 1H, 14-C*H*), 7.97 (dd, *J* = 6.8, 1.1 Hz, 1H, 25-C*H*), 7.87–7.82 (m, 1H, 11-C*H*), 7.79 (d, *J* = 8.6 Hz, 1H, 20-C*H*), 7.75 (dt, *J* = 7.6, 1.0 Hz, 1H, 10-C*H*), 7.46 (dd, *J* = 8.5, 7.0 Hz, 1H, 26-C*H*), 7.44–7.38 (m, 2H, 12-C*H* + 13-C*H*), 7.22 (td, *J* = 7.4, 1.0 Hz, 1H, 9-C*H*), 6.77 (ddd, 8.3, 7.3, 1.2 Hz, 1H, 8-C*H*), 6.67 (d, *J* = 7.9 Hz, 1H, 7-C*H*), 4.36 (p, *J* = 6.7 Hz, 1H, 15-C*H*), 3.61 (dd, *J* = 15.1, 5.7 Hz, 1H, 16-CH_a), 2.80 (d, *J* = 15.2 Hz, 1H, 16-CH_b), 1.39 (d, *J* = 6.7 Hz, 3H, 19-CH_3); ^{13}C NMR (126 MHz, $CDCl_3$) δ 193.71 (28-*C*), 150.49 (6-*C*), 147.91 (17-*C*), 140.15 (2-*C*), 139.81 (1-*C*), 139.59 (3-*C*), 136.91 (5-*C* + 18-*C*), 136.37 (25-*C*), 134.42 (27-*C*), 131.98 (24-*C*), 131.24 (4-*C*), 130.22 (23-*C*), 129.61 (22-*C*), 127.29 (9-*C* + 21-*C*), 127.25 (12-*C*), 127.11 (13-*C*), 127.09 (20-*C*), 126.07 (8-*C*), 125.49 (26-*C*), 125.19 (7-*C*), 124.17 (14-*C*), 119.75 (11-*C*), 119.20 (10-*C*), 45.42 (15-*C*), 41.94 (16-*C*), 19.12 (19-*C*); HRMS (EI) calcd. for $[C_{28}H_{20}O]^+$ 372.15141, found 372.15174.

(±)-(1-(9*H*-Fluoren-9-ylidene)-2-methyl-2,3-dihydro-1*H*-cyclopenta[*a*]naphthalen-6-yl)methanol (7)

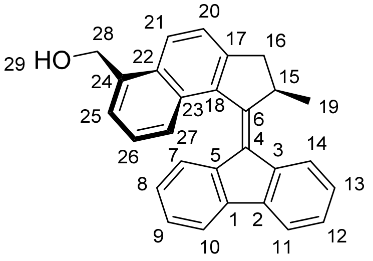

(±)-**7**

Sodium borohydride (15.2 mg, 0.40 mmol, 1.0 equiv) was added to a solution of aldehyde **6** (150 mg, 0.40 mmol, 1.0 equiv) in DCM/MeOH (1:1, v/v, 10 mL). The resulting mixture was stirred at 20 °C for 15 minutes. Upon completion (indicated by TLC, eluent: DCM), aqueous 2M NH_4Cl (40 mL) was added and the product was extracted with DCM (3 × 20 mL). The combined organic extracts were dried over sodium sulfate and the solvent was removed in vacuo to afford alcohol **7** (150 mg, 99%) as a yellow solid. m.p. 116–118; ^{1}H NMR (500 MHz, $CDCl_3$) δ 8.26 (d, *J* = 8.5 Hz, 1H, 21-C*H*), 8.01–7.97 (m, 1H, 14-C*H*), 7.87–7.82 (m, 1H, 11-C*H*), 7.80 (d, *J* = 8.5 Hz, 1H, 27-C*H*), 7.75 (dt, *J* = 7.6, 1.0 Hz, 1H, 10-C*H*), 7.66 (d, *J* = 8.4 Hz, 1H, 20-C*H*), 7.50 (d, *J* = 6.9 Hz, 1H, 25-C*H*), 7.43–7.37 (m, 2H, 12-C*H* + 13-C*H*), 7.28 (dd, *J* = 8.5, 6.9 Hz, 1H, 26-C*H*), 7.21 (td, *J* = 7.4, 1.0 Hz, 1H, 9-C*H*), 6.79 (ddd, 8.3, 7.2, 1.2 Hz, 1H, 8-C*H*), 6.71 (d, *J* = 7.9 Hz, 1H, 7-C*H*), 5.26 (dd, *J* = 12.6, 5.6 Hz, 1H, 28-CH_a), 5.22 (dd, *J* = 12.5, 5.7 Hz, 1H, 28-CH_b), 4.36 (p, *J* = 6.7 Hz, 1H, 15-C*H*), 3.59 (dd, *J* = 15.0, 5.7 Hz, 1H, 16-CH_a), 2.80 (d, *J* = 15.0 Hz, 1H, 16-CH_b), 1.83 (t, *J* = 5.8 Hz, 1H, 29-O*H*), 1.39 (d, *J* = 6.8 Hz, 3H, 19-CH_3); ^{13}C NMR (126 MHz, $CDCl_3$) δ 151.11 (6-*C*), 147.07 (17-*C*), 140.08 (2-*C*), 139.75 (3-*C*), 139.57 (1-*C*), 137.07 (18-*C*), 137.05 (5-*C*), 136.82 (24-*C*), 130.69 (4-*C*), 130.43 (22-*C*), 130.31 (23-*C*), 128.07 (27-*C*), 126.99 (9-*C*), 126.95 (12-*C* + 13-*C*), 126.32 (21-*C*), 126.08 (26-*C*), 125.95 (8-*C*), 125.63 (7-*C*), 125.09 (25-*C*), 124.36 (20-*C*), 124.10 (14-*C*), 119.66 (11-*C*), 118.95 (10-*C*), 64.20 (28-*C*), 45.51 (15-*C*), 41.88 (16-*C*), 19.23 (19-*C*); HRMS (EI) calcd. for $[C_{28}H_{22}O]^+$ 374.16706, found 374.16681.

(±)-9-(2-Methyl-6-((prop-2-yn-1-yloxy)methyl)-2,3-dihydro-1*H*-cyclopenta[*a*]naphthalen-1-ylidene)-9*H*-fluorene (8)

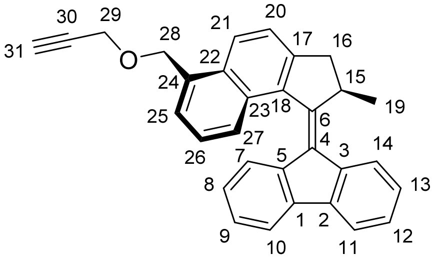

(±)-**8**

A solution of alcohol **7** (105 mg, 0.28 mmol, 1.0 equiv) in dry THF (5 mL) was added at 0 °C to a suspension of sodium hydride (17 mg, 0.42 mmol, 1.5 equiv, 60% in mineral oil) in dry THF (2 mL) under an argon atmosphere. The mixture was stirred at 0 °C for 70 minutes and then propargyl bromide (0.31 mL, 2.8 mmol, 10 equiv, 80% in toluene) was added. The resulting mixture was stirred at 20 °C for 21 hours. Upon completion (indicated by TLC, eluent: DCM), the reaction was quenched with water (40 mL) and the product was extracted with DCM (3 × 30 mL). The combined organic extracts were dried over sodium sulfate and the solvent was removed in vacuo. The residue was purified by silica gel column chromatography (eluent: DCM/pentane, 1:1, v/v) to afford alkyne **8** (104 mg, 90%) as a yellow solid. m.p. 75–77 °C; ^{1}H NMR (500 MHz, $CDCl_3$) δ 8.27 (d, *J* = 8.5 Hz, 1H, 21-C*H*), 8.00–7.96 (m, 1H, 14-C*H*), 7.87–7.82 (m, 1H, 11-C*H*), 7.82 (d, *J* = 8.4 Hz, 1H, 27-C*H*), 7.75 (dt, *J* = 7.5, 0.9 Hz, 1H, 10-C*H*), 7.65 (d, *J* = 8.4 Hz, 1H, 20-C*H*), 7.49 (dd, *J* = 6.9, 1.2 Hz, 1H, 25-C*H*), 7.42–7.36 (m, 2H, 12-C*H* + 13-C*H*), 7.26 (dd, *J* = 8.5, 6.9 Hz, 1H, 26-C*H*), 7.21 (td, *J* = 7.4, 1.1 Hz, 1H, 9-C*H*), 6.79 (ddd, 8.2, 7.2, 1.2 Hz, 1H, 8-C*H*), 6.71 (d, *J* = 7.9 Hz, 1H, 7-C*H*), 5.14 (d, *J* = 11.4 Hz, 1H, 28-CH_a), 5.11 (d, *J* = 11.3 Hz, 1H, 28-CH_b), 4.35 (p, *J* = 6.5 Hz, 1H, 15-C*H*), 4.32 (d, *J* = 2.4 Hz, 2H, 29-CH_2), 3.58 (dd, *J* = 15.0, 5.7 Hz, 1H, 16-CH_a), 2.76 (d, *J* = 15.1 Hz, 1H, 16-CH_b), 2.56 (t, *J* = 2.4 Hz, 1H, 31-C*H*), 1.39 (d, *J* = 6.8 Hz, 3H, 19-CH_3); ^{13}C NMR (126 MHz, $CDCl_3$) δ 151.18 (6-*C*), 147.10 (17-*C*), 140.10 (2-*C*), 139.78 (3-*C*), 139.56 (1-*C*), 137.09 (5-*C*), 136.98 (18-*C*), 133.33 (24-*C*), 131.05 (22-*C*), 130.66 (4-*C*), 130.32 (23-*C*), 128.49 (27-*C*), 126.97 (9-*C*), 126.94 (12-*C* + 13-*C*), 126.88 (25-*C*), 126.73 (21-*C*), 126.01 (8-*C*), 125.89 (26-*C*), 125.69 (7-*C*), 124.41 (20-*C*), 124.11 (14-*C*), 119.66 (11-

C), 118.94 (10-C), 79.69 (30-C), 74.94 (31-C), 70.60 (28-C), 57.42 (29-C), 45.55 (15-C), 41.92 (16-C), 19.26 (19-C); HRMS (EI) calcd. for $[C_{31}H_{24}O]^+$ 412.18271, found 412.18228.

(±)-Zinc(II) azide cage (Zn11)

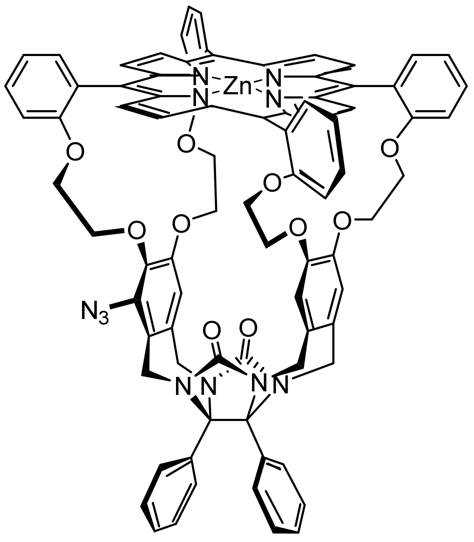

(±)-**Zn11**

A Schlenk bomb was charged with tin(II) chloride (1.5 g, 8.0 mmol, 40 equiv) and mononitro cage **$H_2$9** (0.28 g, 0.20 mmol, 1.0 equiv). 4M HCl in dioxane (7 mL) and aqueous 37% HCl (1 drop) were successively added, the bomb was closed, and the mixture was vigorously stirred at 60 °C for 5 hours. Upon completion (indicated by TLC, eluent: chloroform/acetonitrile, 9:1, v/v), the reaction was quenched with aqueous 3M NaOH (60 mL) and the product was extracted with chloroform (2 × 50 mL). The combined organic extracts were successively washed with aqueous 1M NaOH (50 mL); then dried over sodium sulfate and the solvent was removed in vacuo to afford crude amine **$H_2$10** as a purple solid. The solid material was dissolved in chloroform/acetonitrile (60 mL, 3:1, v/v). ^{t}BuONO (0.16 mL, 1.4 mmol, 7.0 equiv) and $TMSN_3$ (0.16 mL, 1.2 mmol, 6.0 equiv) were added and the mixture was stirred at 20 °C for 1.5 hours. Upon completion (indicated by TLC, eluent: chloroform/acetonitrile, 9:1, v/v), the mixture was evaporated to dryness. The residue was purified by Alumina III column chromatography (eluent $CHCl_3$) to afford azide cage **$H_2$11** as a purple solid. The latter solid was dissolved in chloroform/methanol (75 mL, 2:1, v/v), zinc acetate dihydrate (0.28 g, 1.5 mmol, 7.5 equiv) was added, and the resulting mixture was stirred at 20 °C for 20 hours. Upon completion (indicated by MALDI-TOF), the mixture was evaporated to dryness. The residue was purified by silica gel column chromatography (eluent: chloroform/methanol, 99:1, v/v). Subsequently, the purified material was dissolved in a minimal amount of DCM and precipitated by the addition of heptane. Most of the DCM was removed under reduced pressure and the resulting suspension was centrifuged. The supernatant was removed and the precipitate was washed with pentane and dried under high vacuum to afford zinc azide porphyrin cage compound **Zn11** (0.26 g, 90% over 3 steps from **$H_2$9**) as a purple solid. m.p. > 300 °C (dec.); ^{1}H NMR (500 MHz, $CDCl_3$) δ 8.93 (d, J = 4.6 Hz, 1H), 8.90 (d, J = 4.6 Hz, 1H), 8.86 (d, J = 4.6 Hz, 1H), 8.85 (d, J = 4.6 Hz, 1H), 8.78 (d, J = 4.6 Hz, 1H), 8.77 (d, J = 4.6 Hz, 1H), 8.76 (d, J = 4.6 Hz, 1H), 8.73 (d, J = 4.6 Hz, 1H), 8.36 (dd, J = 7.3, 1.6 Hz, 1H), 8.17 (dd, J = 7.3, 1.7 Hz, 1H), 8.05 (dd, J = 7.3, 1.7 Hz, 1H), 8.02 (dd, J = 7.3, 1.7 Hz, 1H), 7.80–7.71 (m, 4H), 7.48 (td, J = 7.7, 1.2 Hz, 1H), 7.41 (td, J = 7.5, 1.1 Hz, 1H), 7.39–7.29 (m, 6H), 7.04–6.99 (m, 2H), 6.97–6.92 (m, 2H), 6.92–6.86 (m, 2H), 6.86–6.79 (m, 2H), 6.72 (d, J = 8.2 Hz, 1H), 6.69 (d, J = 8.2 Hz, 1H), 6.24 (s, 1H), 6.07 (s, 1H), 5.91 (s, 1H), 4.90 (d, J = 16.3 Hz, 1H), 4.26–4.16 (m, 5H), 4.16–4.00 (m, 5H), 3.96 (ddd, J = 10.7, 4.1, 2.3 Hz, 1H), 3.72 (d, J = 15.7 Hz, 1H), 3.72–3.67 (m, 1H), 3.69 (d, J = 15.9 Hz, 1H), 3.65 (d, J = 15.6 Hz, 1H), 3.59 (ddd, J = 10.7, 4.1, 2.3 Hz, 1H), 3.47 (dt, J = 10.0, 4.9 Hz, 1H), 3.41 (ddd, J = 10.9, 6.8, 4.5 Hz, 1H), 3.36 (d, J = 16.3 Hz, 1H), 3.32–3.21 (m, 2H), 3.21–3.15 (m, 1H), 2.69 (td, J = 8.8, 6.0 Hz, 1H); ^{13}C NMR (126 MHz, $CDCl_3$) δ 159.05, 158.92, 158.85, 158.78, 156.73, 156.51, 150.49, 150.18, 150.16, 150.12, 149.84, 149.68, 149.63, 147.02, 145.66, 140.43, 135.77, 135.64, 135.50, 134.47, 133.37, 133.29, 132.77, 132.71, 132.57, 132.48, 132.77, 131.48, 131.40, 131.30, 131.20, 131.11, 131.02, 130.90, 130.81, 130.67, 130.61, 129.60, 129.42, 129.40, 129.35, 128.94, 128.56, 128.41, 128.32, 128.24, 127.71, 127.58, 122.32, 120.21, 119.96, 119.87, 119.75, 116.74, 116.15, 116.06, 116.03, 115.90, 113.75, 112.65, 112.38, 112.17, 112.08, 109.89, 84.73, 84.41, 70.79, 67.91, 67.50, 67.21, 67.00, 66.89, 66.82, 65.34, 44.37, 44.06, 38.25; HRMS (ESI) calcd. for $[C_{84}H_{61}N_{11}O_{10}Zn + Cs]^+$ 1580.29488, found 1580.30136.

Zinc(II) cage molecular motor hybrid (Zn2)

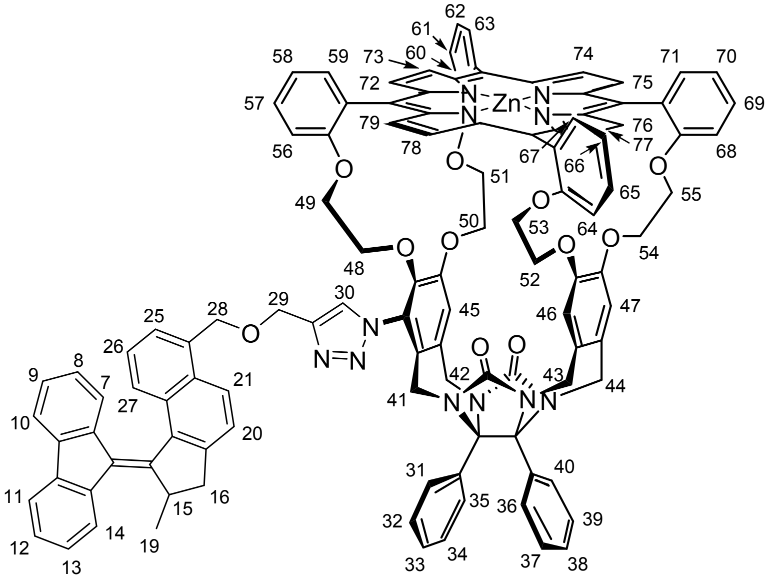

Zn2

Sodium ascorbate (164 mg, 0.83 mmol, 10 equiv), copper(I) iodide (236 mg, 1.24 mmol, 15 equiv) and DIPEA (0.14 mL, 0.83 mmol, 10 equiv) were successively added to a solution of **Zn11** (120 mg, 83 µmol, 1.0 equiv) and alkyne motor **8** (51 mg, 124 µmol, 1.5 equiv) in a degassed mixture of DMSO (50 mL) and water (3 mL). The resulting mixture was stirred at 20 °C for 3 days under an argon atmosphere. Meanwhile, after 2 days, additional alkyne motor **8** (9 mg, 20 µmol, 0.25 equiv) and copper(I) iodide (40 mg, 0.2 mmol, 2.5 equiv) were added. Upon completion (indicated by TLC, eluent: chloroform/acetonitrile, 9:1, v/v), the mixture was diluted with water (150 mL) and the product was extracted with chloroform (3 × 50 mL). The combined organic extracts were washed with water (3 × 100 mL) and brine (100 mL); then dried over sodium sulfate and the solvent was removed in vacuo. The residue was purified by column chromatography (60H silica gel, eluent: chloroform/acetonitrile, 10:1, v/v). Excess alkyne motor **8** (29.0 mg) was recovered as a yellow solid. The first eluted pair of enantiomers (**Zn2a**, 58.3 mg, 38%) was isolated as a purple solid after precipitation from DCM/heptane (1:1 v/v). The second eluted pair of enantiomers (**Zn2b**, 55.0 mg, 36%) was also obtained as a purple solid after precipitation from the same solvent mixture. (±)-**Zn2a** (first eluted fraction): m.p. > 300 °C (dec.); ^{1}H NMR (500 MHz, 1.0 mM in $CDCl_3$, peak width and chemical shift of ^{1}H NMR signals were highly concentration dependent) δ 8.91 (d, J = 4.6 Hz, 1H, 75-C*H*), 8.88 (d, J = 4.6 Hz, 1H, 74-C*H*), 8.78 (s, 2H, 76-C*H* + 77-C*H*), 8.75 (d, J = 4.6 Hz, 1H, 79-C*H*), 8.73 (d, J = 4.6 Hz, 1H, 78-C*H*), 8.70 (s, 2H, 72-C*H* + 73-C*H*), 8.24 (d, J = 8.6 Hz, 1H, 21-C*H*), 8.24 (dd, J = 7.2, 1.8 Hz, 1H, 59-C*H*), 8.14 (dd, J = 7.3, 1.6 Hz, 1H, 71-C*H*), 8.05 (dd, J = 7.3, 1.7 Hz, 1H, 67-C*H*), 8.06–8.03 (m, 1H, 14-C*H*), 8.03 (dd, J = 7.4, 1.5 Hz, 1H, 63-C*H*), 7.91–7.87 (m, 1H, 11-C*H*), 7.85 (d, J = 8.4 Hz, 1H, 27-C*H*), 7.80 (dt, J = 7.7, 1.1 Hz, 1H, 10-C*H*), 7.79–7.74 (m, 2H, 61-C*H* + 69-C*H*), 7.70 (td, J = 8.3, 1.7 Hz, 1H, 65-C*H*), 7.57 (d, J = 8.6 Hz, 1H, 20-C*H*), 7.55 (s, 1H, 30-C*H*), 7.49 (td, J = 8.0, 1.8 Hz, 1H, 57-C*H*), 7.46–7.41 (m, 2H, 12-C*H* + 13-C*H*), 7.42–7.32 (m, 7H, 25-C*H* + 58-C*H* + 60-C*H* + 62-C*H* + 66-C*H* + 68-C*H* + 70-C*H*), 7.28 (d, J = 8.2 Hz, 1H, 64-C*H*), 7.27–7.23 (m, 1H, 9-C*H*), 7.22 (dd, J = 8.4, 7.0 Hz, 1H, 26-C*H*), 7.00 (td, J = 7.5, 1.1 Hz, 1H, 34-C*H*), 7.00 (d, J = 8.2 Hz, 1H, 56-C*H*), 6.96–6.82 (m, 7H, 7-C*H* + 8-C*H* + 32-C*H* + 33-C*H* + 37-C*H* + 38-C*H* + 39-C*H*), 6.79 (d, J = 8.2 Hz, 1H, 35-C*H*), 6.75–6.71 (m, 1H, 36-C*H*), 6.72–6.67 (m, 1H, 40-C*H*), 6.62 (d, J = 8.1 Hz, 1H, 31-C*H*), 6.31 (s, 1H, 45-C*H*), 6.23 (s, 1H, 47-C*H*), 6.08 (s, 1H, 46-C*H*), 4.96 (d, J = 11.9 Hz, 1H, 28-CH_a), 4.92 (d, J = 11.8 Hz, 1H, 28-CH_b), 4.79 (d, J = 12.6 Hz, 1H, 29-CH_a), 4.75 (d, J = 12.6 Hz, 1H, 29-CH_b), 4.40 (p, J = 6.6 Hz, 1H, 15-C*H*), 4.25 (d, J = 15.9 Hz, 1H, 42-CH_a), 4.22 (ddd, J = 11.1, 6.9, 4.3 Hz, 1H, 55-CH_a), 4.19 (ddd, J = 10.6, 8.6, 2.7 Hz, 1H, 53-CH_a), 4.16–4.10 (m, 1H, 51-CH_a), 4.08 (d, J = 16.1 Hz, 2H, 43-CH_a + 44-CH_a), 4.08–4.02 (m, 2H, 53-CH_b + 55-CH_b), 3.89–3.81 (m, 2H, 49-CH_a + 51-CH_b), 3.80–3.72 (m, 1H, 50-CH_a), 3.76 (d, J = 16.4 Hz, 1H, 41-CH_a), 3.76 (d, J = 15.9 Hz, 1H, 42-CH_b), 3.67 (d, J = 15.8 Hz, 1H, 44-CH_b), 3.66 (d, J = 15.8 Hz, 1H, 43-CH_b), 3.59 (dd, J = 15.3, 6.1 Hz, 1H, 16-CH_a), 3.62–3.55 (m, 1H, 52-CH_a), 3.55 (d, J = 16.7 Hz, 1H, 41-CH_b), 3.55–3.48 (m, 2H, 49-CH_b +

54-CH_a), 3.37 (ddd, J = 10.8, 7.1, 4.1 Hz, 1H, 54-CH_b), 3.33–3.26 (m, 2H, 50-CH_b + 52-CH_b), 3.23 (ddd, J = 10.1, 6.1, 4.2 Hz, 1H, 48-CH_a), 2.96 (dt, J = 9.7, 7.2 Hz, 1H, 48-CH_b), 2.76 (d, J = 14.9 Hz, 1H, 16-CH_b), 1.44 (d, J = 6.7 Hz, 3H, 19-CH_3); ^{13}C NMR (126 MHz, 10 mM in $CDCl_3$) δ 159.18, 159.14, 158.99, 156.68, 156.49, 151.66, 150.77, 150.42, 150.36, 150.25, 150.23, 149.98, 149.86, 149.80, 149.73, 147.23, 147.04, 146.28, 143.48, 142.52, 140.31, 140.02, 139.68, 137.31, 136.99, 135.76, 135.60, 134.79, 134.39, 133.15, 133.12, 132.79, 132.77, 132.67, 132.32, 132.18, 131.58, 131.42, 131.37, 131.27, 130.86, 130.84, 130.69, 130.46, 130.43, 129.86, 129.76, 129.57, 129.33, 128.88, 128.83, 128.77, 128.71, 128.54, 128.24, 128.18, 127.84, 127.68, 127.45, 127.32, 127.09, 127.08, 126.93, 126.87, 126.33, 126.30, 125.99, 124.51, 124.28, 120.24, 120.14, 120.09, 120.06, 119.84, 119.11, 116.46, 116.37, 116.15, 116.12, 116.08, 115.28, 115.21, 112.89, 112.55, 112.38, 112.10, 84.85, 84.41, 77.41, 77.16, 76.91, 71.50, 70.64, 67.96, 67.76, 67.60, 67.54, 67.36, 67.22, 67.17, 63.04, 45.82, 44.39, 44.22, 44.14, 42.09, 38.65, 19.52; UV-vis ($CHCl_3$/CH_3CN, 1:1, v/v) λ/nm (log(ε/M^{-1}·cm^{-1})) 425 (5.68), 556 (4.40), 595 (4.01); Fluorescence ($CHCl_3$/CH_3CN, 1:1, v/v, λ_{ex} = 550 nm) λ_{em}/nm 607, 656; Fluorescence ($CHCl_3$/CH_3CN, 1:1, v/v, λ_{ex} = 365 nm) λ_{em}/nm 438, 607, 656.

(±)-**Zn2b** (second eluted fraction): m.p. > 300 °C (dec.); ^{1}H NMR (500 MHz, 1.0 mM in $CDCl_3$, peak width and chemical shift of ^{1}H NMR signals were highly concentration dependent) δ 8.90 (d, J = 4.6 Hz, 1H, 75-CH), 8.88 (d, J = 4.6 Hz, 1H, 74-CH), 8.74 (d, J = 4.6 Hz, 1H, 76-CH), 8.70 (s, 2H, 72-CH + 73-CH), 8.69 (d, J = 4.6 Hz, 1H, 77-CH), 8.34 (d, J = 8.4 Hz, 1H, 21-CH), 8.30 (d, J = 4.6 Hz, 1H, 79-CH), 8.23 (d, J = 4.6 Hz, 1H, 78-CH), 8.16 (d, J = 7.9 Hz, 1H, 14-CH), 8.14 (dd, J = 7.3, 1.6 Hz, 1H, 59-CH), 8.06 (dd, J = 7.4, 1.7 Hz, 1H, 71-CH), 8.05 (dd, J = 7.2, 1.7 Hz, 1H, 63-CH), 7.94 (d, J = 7.5 Hz, 1H, 11-CH), 7.88 (dd, J = 7.3, 1.7 Hz, 1H, 67-CH), 7.79–7.72 (m, 3H, 10-CH + 61-CH + 69-CH), 7.65 (d, J = 8.4 Hz, 1H, 20-CH), 7.61 (d, J = 8.5 Hz, 1H, 27-CH), 7.58 (td, J = 7.8, 1.2 Hz, 1H, 13-CH), 7.57–7.52 (m, 1H, 57-CH), 7.52 (t, J = 7.4 Hz, 1H, 12-CH), 7.44 (td, J = 8.0, 1.7 Hz, 1H, 65-CH), 7.44 (s, 1H, 30-CH), 7.42–7.34 (m, 4H, 60-CH + 62-CH + 68-CH + 70-CH), 7.31 (td, J = 7.5, 1.1 Hz, 1H, 58-CH), 7.22 (t, J = 7.4 Hz, 1H, 66-CH), 7.17 (td, J = 7.4, 1.0 Hz, 1H, 9-CH), 7.06 (d, J = 8.3 Hz, 1H, 56-CH), 7.01–6.96 (m, 1H, 34-CH), 6.96–6.91 (m, 4H, 33-CH + 37-CH + 38-CH + 39-CH), 6.89–6.84 (m, 2H, 8-CH + 32-CH), 6.81 (d, J = 7.8 Hz, 1H, 35-CH), 6.78 (d, J = 8.6 Hz, 1H, 64-CH), 6.78–6.73 (m, 3H, 7-CH + 36-CH + 40-CH), 6.67 (d, J = 8.1 Hz, 1H, 31-CH), 6.56 (d, J = 6.8 Hz, 1H, 25-CH), 6.34 (s, 1H, 45-CH), 6.33–6.28 (m, 1H, 26-CH), 6.20 (s, 1H, 47-CH), 5.99 (s, 1H, 46-CH), 4.90 (d, J = 12.4 Hz, 1H, 28-CH_a), 4.72 (d, J = 12.9 Hz, 1H, 29-CH_a), 4.54 (d, J = 12.5 Hz, 1H, 28-CH_b), 4.43 (p, J = 6.5 Hz, 1H, 15-CH), 4.42 (d, J = 12.9 Hz, 1H, 29-CH_b), 4.27 (d, J = 15.9 Hz, 1H, 42-CH_a), 4.24 (ddd, J = 10.9, 7.5, 3.6 Hz, 1H, 55-CH_a), 4.17 (d, J = 15.9 Hz, 1H, 43-CH_a), 4.13 (d, J = 16.0 Hz, 1H, 44-CH_a), 4.12–4.03 (m, 2H, 51-CH_a + 55-CH_b), 3.99 (ddd, J = 10.6, 8.7, 2.1 Hz, 1H, 53-CH_a), 3.88 (dt, J = 8.2, 3.8 Hz, 1H, 49-CH_a), 3.84–3.58 (m, 9H, 41-CH_a + 42-CH_b + 43-CH_b + 44-CH_b + 49-CH_b + 50-CH_a + 51-CH_b + 53-CH_b + 54-CH_a), 3.64 (dd, J = 14.5, 5.6 Hz, 1H, 16-CH_a), 3.55 (d, J = 16.4 Hz, 1H, 41-CH_b), 3.39 (ddd, J = 11.1, 7.7, 3.5 Hz, 1H, 54-CH_b), 3.37–3.32 (m, 1H, 48-CH_a), 3.33–3.27 (m, 2H, 48-CH_b + 50-CH_b), 3.25–3.20 (m, 1H, 52-CH_a), 3.15–3.09 (m, 1H, 52-CH_b), 2.85 (d, J = 14.8 Hz, 1H, 16-CH_b), 1.46 (d, J = 6.7 Hz, 3H, 19-CH_3); ^{13}C NMR (126 MHz, 10 mM in $CDCl_3$) δ 159.16, 159.09, 158.73, 158.33, 156.60, 156.54, 152.26, 150.53, 150.30, 150.06, 149.95, 149.66, 149.56, 149.49, 146.93, 146.75, 146.01, 142.51, 142.41, 140.03, 139.87, 139.57, 137.29, 136.77, 135.76, 135.69, 135.62, 134.94, 133.26, 133.25, 132.88, 132.59, 132.03, 131.93, 131.81, 131.46, 131.32, 131.10, 130.97, 130.66, 130.61, 130.55, 130.41, 130.13, 129.95, 129.49, 129.45, 129.38, 129.35, 129.32, 129.01, 128.74, 128.64, 128.60, 128.57, 128.46, 128.30, 128.08, 127.83, 127.71, 127.43, 127.39, 127.14, 126.86, 126.62, 126.37, 126.09, 125.99, 124.51, 124.10, 120.21, 119.90, 119.72, 119.47, 118.91, 116.30, 116.01, 115.90, 115.74, 115.39, 115.09, 114.47, 113.14, 112.17, 112.10, 111.26, 84.77, 84.35, 71.47, 68.96, 67.86, 67.78, 67.54, 67.16, 66.97, 66.95, 66.41, 60.78, 45.55, 44.38, 44.17, 44.10, 42.00, 38.47, 19.37; UV-vis ($CHCl_3$/CH_3CN, 1:1, v/v) λ/nm (log(ε/M^{-1}·cm^{-1})) 426 (5.63), 557 (4.40), 596 (4.06); Fluorescence ($CHCl_3$/CH_3CN, 1:1, v/v, λ_{ex} = 550 nm) λ_{em}/nm 607, 656; Fluorescence ($CHCl_3$/CH_3CN, 1:1, v/v, λ_{ex} = 365 nm) λ_{em}/nm 439, 607, 656; HRMS (ESI) calcd. for $[C_{115}H_{85}N_{11}O_{11}Zn + Na]^+$ 1882.56192, found 1882.56760.

Chiral HPLC chromatograms

Samples of **Zn2a** (Supplementary Figure 1) or **Zn2b** (Supplementary Figure 2) were dissolved in dichloromethane, injected on a Chiralpak ID or Chiralpak IE column, using ethanol/dichloromethane (30:70, v/v) as the mobile phase. The flow rate was 1 mL/min and detection was performed with a UV detector at λ = 420 nm.

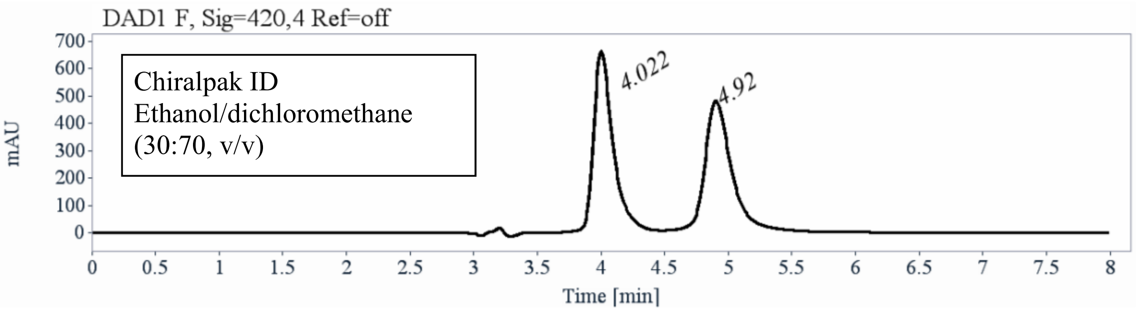

Supplementary Figure 1. HPLC Chromatograms. HPLC chromatogram of (±)-**Zn2a** with UV detection at λ = 420 nm. The indicated retention times correspond to the two enantiomers of **Zn2a**.

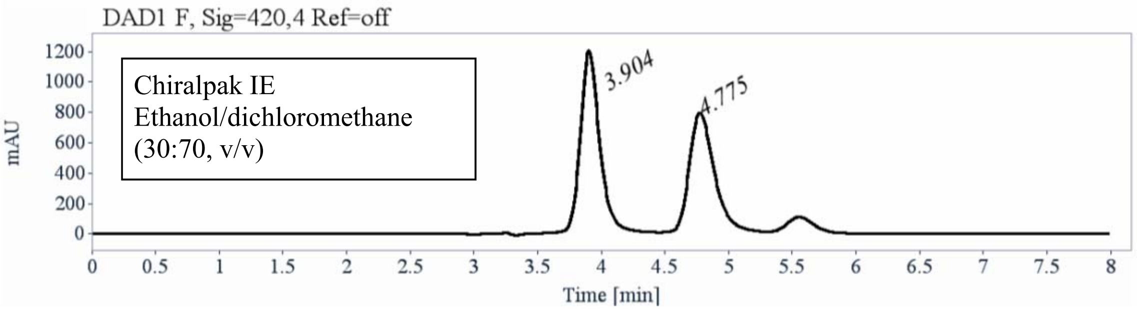

Supplementary Figure 1. HPLC Chromatograms. HPLC chromatogram of (±)-**Zn2b** with UV detection at λ = 420 nm. The indicated retention times correspond to the two enantiomers of **Zn2b**.

Relevant ROESY correlations for Zn2a and Zn2b

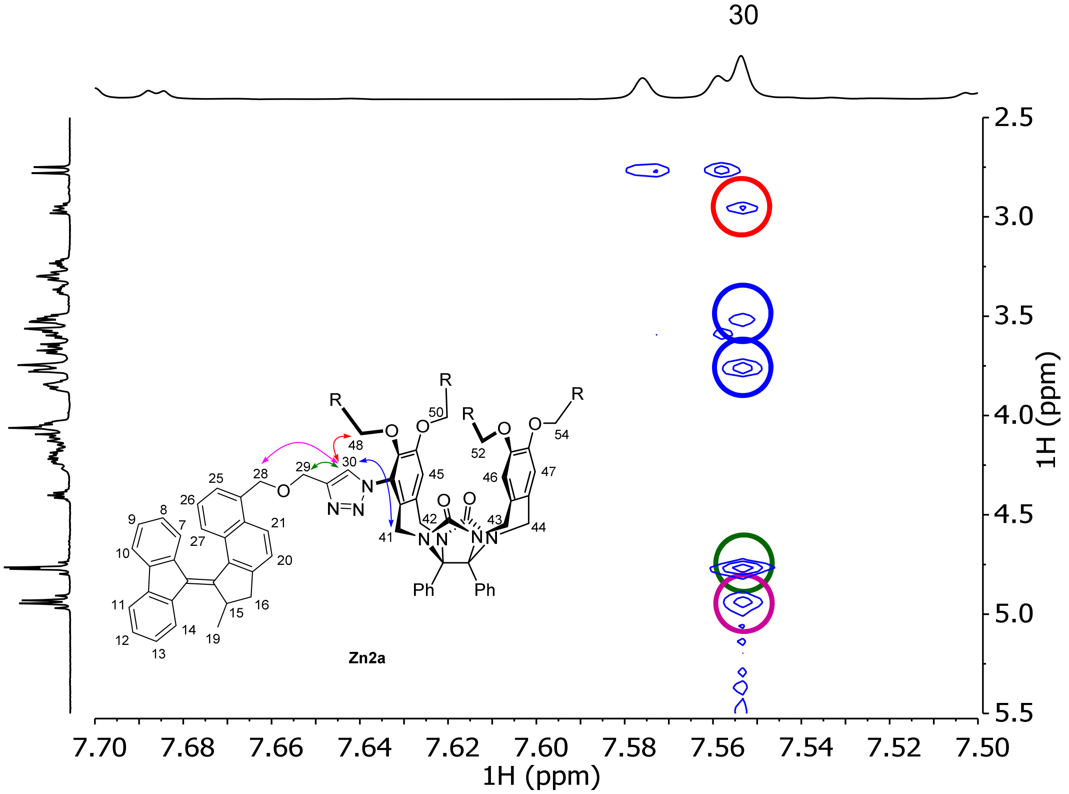

Supplementary Figure 2. ROESY correlations. Relevant ROESY correlations of triazole proton 30 of compound **Zn2a**, showing the connectivity of the motor and cage moieties (500 MHz, 1.0 mM in $CDCl_3$).

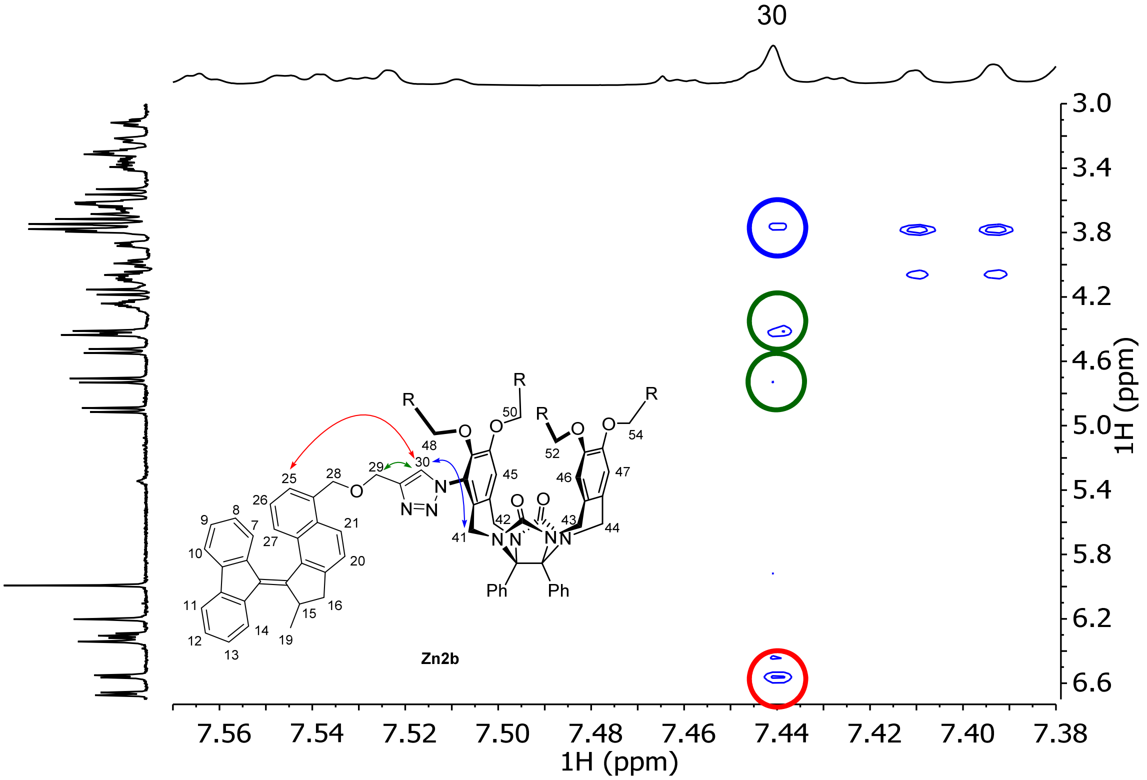

Supplementary Figure 3. ROESY correlations. Relevant ROESY correlations of triazole proton 30 of compound **Zn2b**, showing the connectivity of the motor and cage moieties (green and blue), as well as the proximity of the upper half of the molecular motor and the cavity of the cage compound (red) (500 MHz, 1.0 mM in $CDCl_3$).

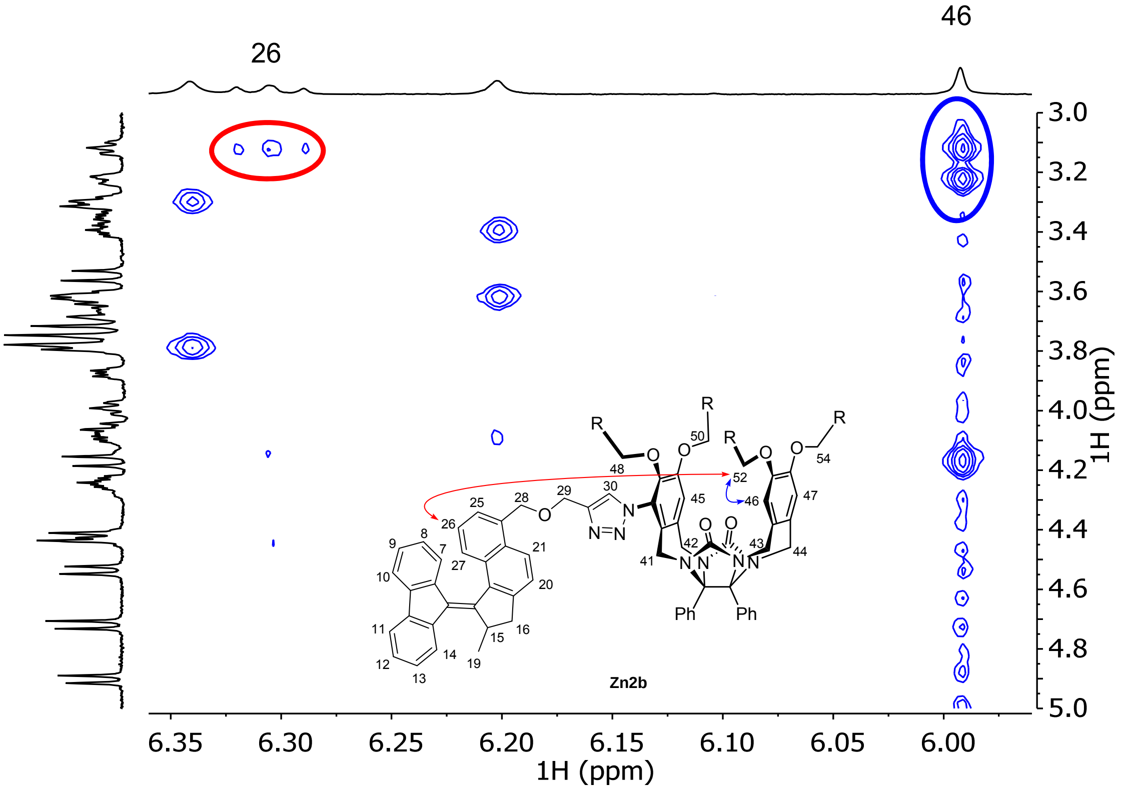

Supplementary Figure 4. ROESY correlations. Relevant ROESY correlations of compound **Zn2b**, showing the proximity of the upper half of the molecular motor and the cavity of the cage compound (500 MHz, 1.0 mM in $CDCl_3$).

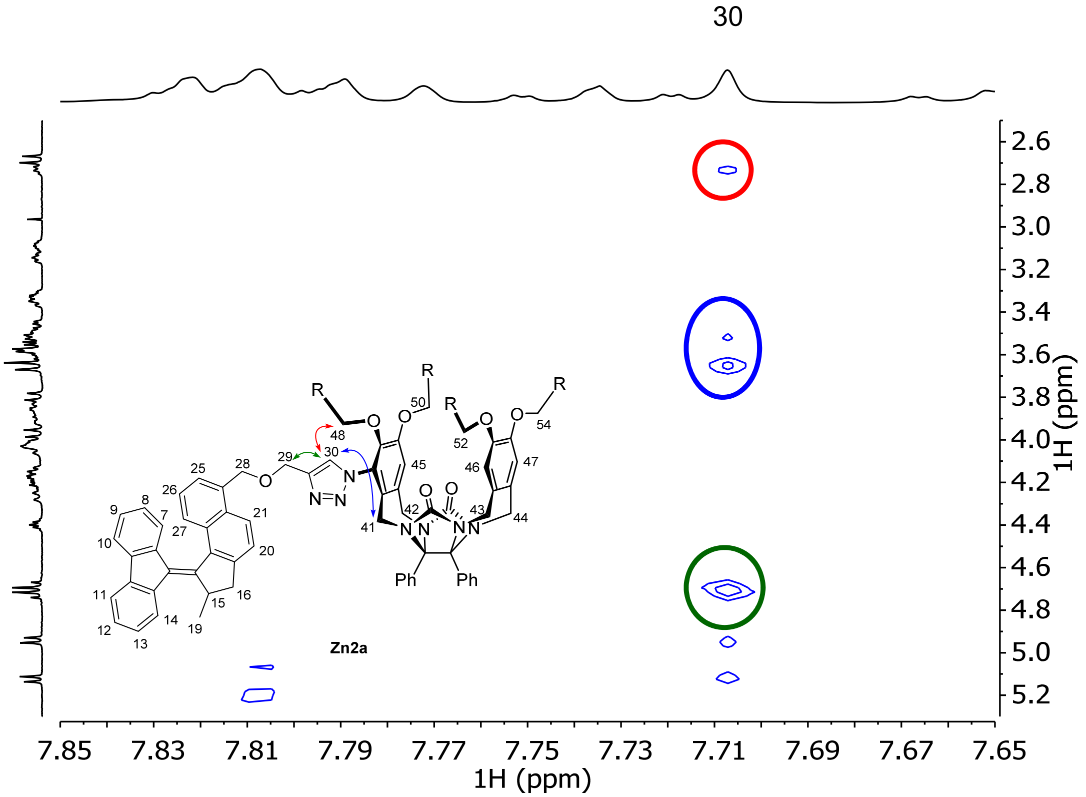

Supplementary Figure 5. ROESY correlations. Relevant ROESY correlations of triazole proton 30 of compound **Zn2a**, showing the connectivity of the motor and cage moieties (500 MHz, 1.0 mM in $CDCl_3$/CD_3CN, 1:1, v/v).

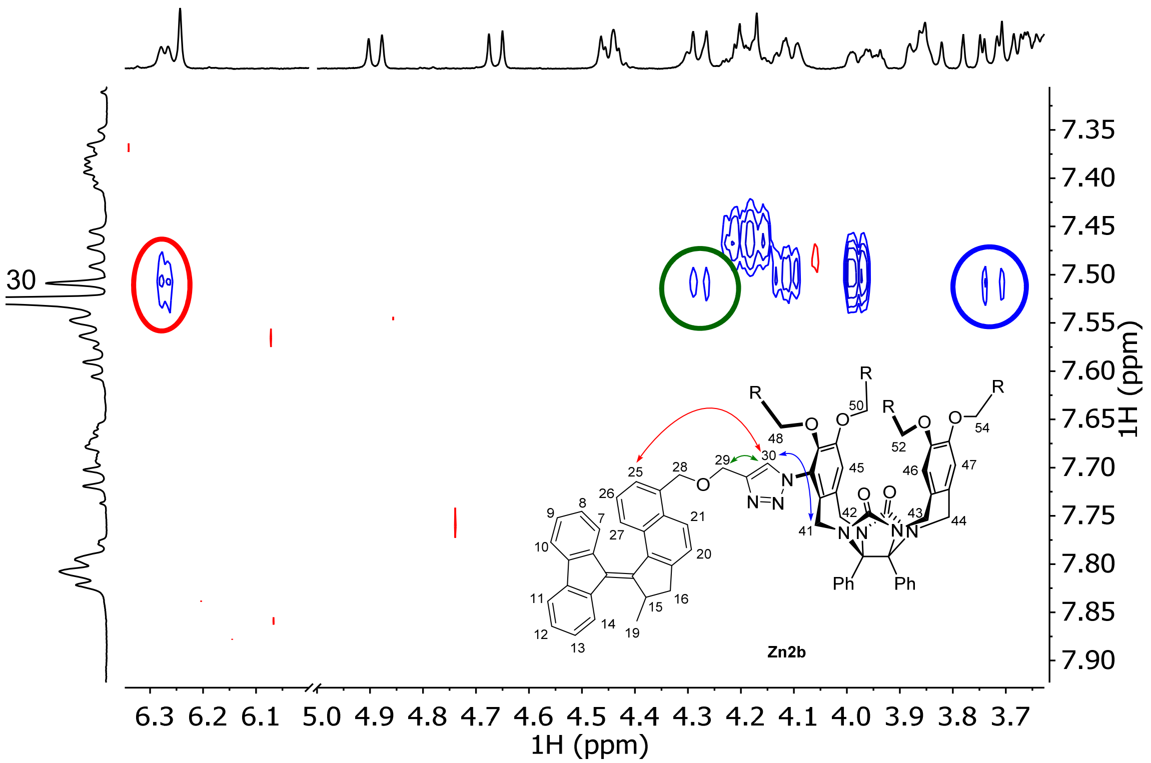

Supplementary Figure 6. ROESY correlations. Relevant ROESY correlations of triazole proton 30 of compound **Zn2b**, showing the connectivity of the motor and cage moieties (green and blue), as well as the proximity of the upper half of the molecular motor and the cavity of the cage compound (red) (500 MHz, 1.0 mM in $CDCl_3/CD_3CN$, 1:1, v/v).

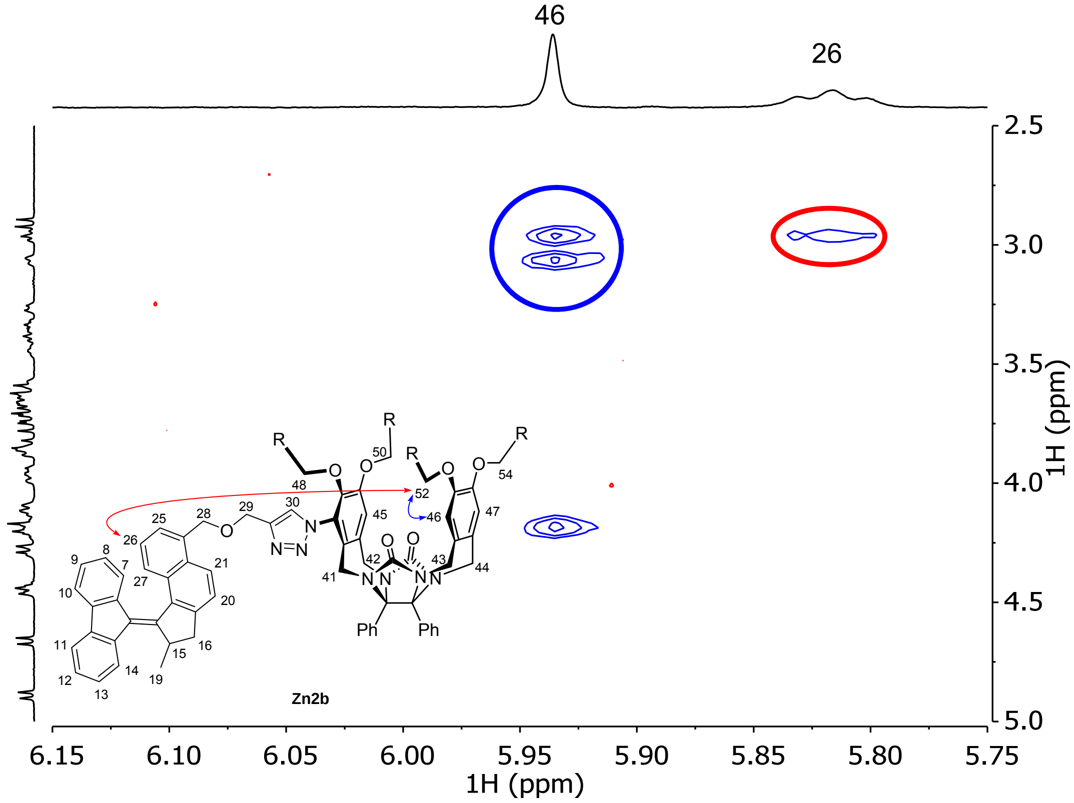

Supplementary Figure 7. ROESY correlations. Relevant ROESY correlations of compound **Zn2b**, showing the proximity of the upper half of the molecular motor and the cavity of the cage compound (500 MHz, 1.0 mM in $CDCl_3/CD_3CN$, 1:1, v/v).

Titrations of Zn2a and Zn2b with methyl viologen (V12)

Binding constants were determined using the supramolecular.org web application.[9,10]

Supplementary Table 1. Association constants of **Zn2a** with **V12** ($[host]_0 = 10^{-6}$ in $CHCl_3/CH_3CN$, 1:1, v/v, 298K).

Host	Guest	K_{assoc} ($\times 10^5$ M^{-1})	Error (%)	URL
(±)-**Zn2a**	**V12**	8.3	3.4	http://app.supramolecular.org/bindfit/view/7d9f908e-dd6e-4e1f-86c7-77dd4f47a793
(±)-**Zn2a**	**V12**	9.9	3.0	http://app.supramolecular.org/bindfit/view/c9ef4734-f06d-4758-925b-7dd86dca1016
(±)-**Zn2a**	**V12**	9.0	4.1	http://app.supramolecular.org/bindfit/view/af6f0a09-dc8a-4e6a-b6a7-127cbe5810ff
**AVERAGE**		9.1 ± 0.7		

Supplementary Table 2. Association constants of **Zn2b** with **V12** ($[host]_0 = 10^{-6}$ in $CHCl_3/CH_3CN$, 1:1, v/v, 298K).

Host	Guest	K_{assoc} ($\times 10^5$ M^{-1})	Error (%)	URL
(±)-**Zn2b**	**V12**	5.8	7.0	http://app.supramolecular.org/bindfit/view/d00d5294-0e5a-4baa-8eea-4873dc71b93f
(±)-**Zn2b**	**V12**	6.3	7.3	http://app.supramolecular.org/bindfit/view/0148c916-a331-450c-962d-6239fb5d7625
(±)-**Zn2b**	**V12**	7.3	8.0	http://app.supramolecular.org/bindfit/view/01733967-bcc1-4934-85e9-fd951799cfc2
**AVERAGE**		6.5 ± 0.6		

Host–Guest complexes of Zn2a and Zn2b with benzyl viologen (V13)

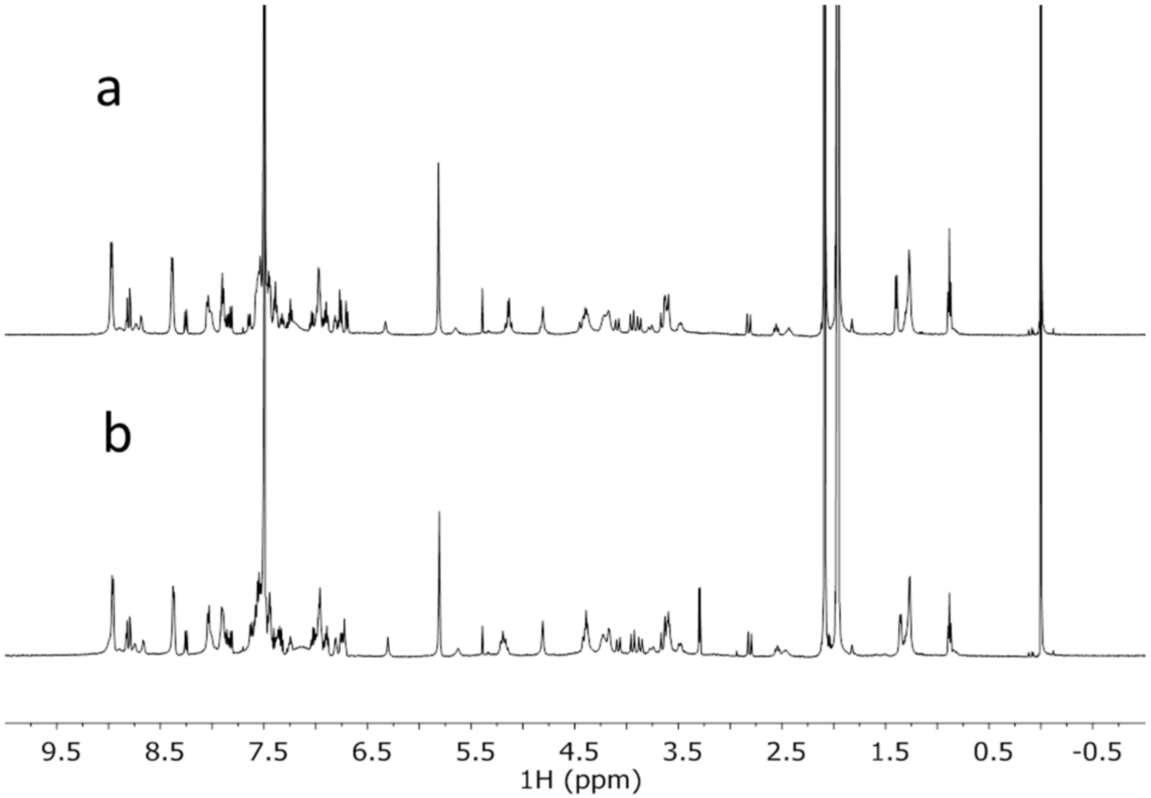

Supplementary Figure 9. NMR spectra of host-guest complexes. a 500 MHz ^{1}H NMR spectrum of **Zn2a·V13** (3 equivalents of **V13** with respect to **Zn2a**). **b** 500 MHz ^{1}H NMR spectrum of **Zn2b·V13** (3 equivalents of **V13** with respect to **Zn2b**) (c = 10^{-3} in $CDCl_3/CD_3CN$, 1:1, v/v, 298K).

Host–Guest complexes of Zn2a and Zn2b with cyclohexylmethyl viologen (V14)

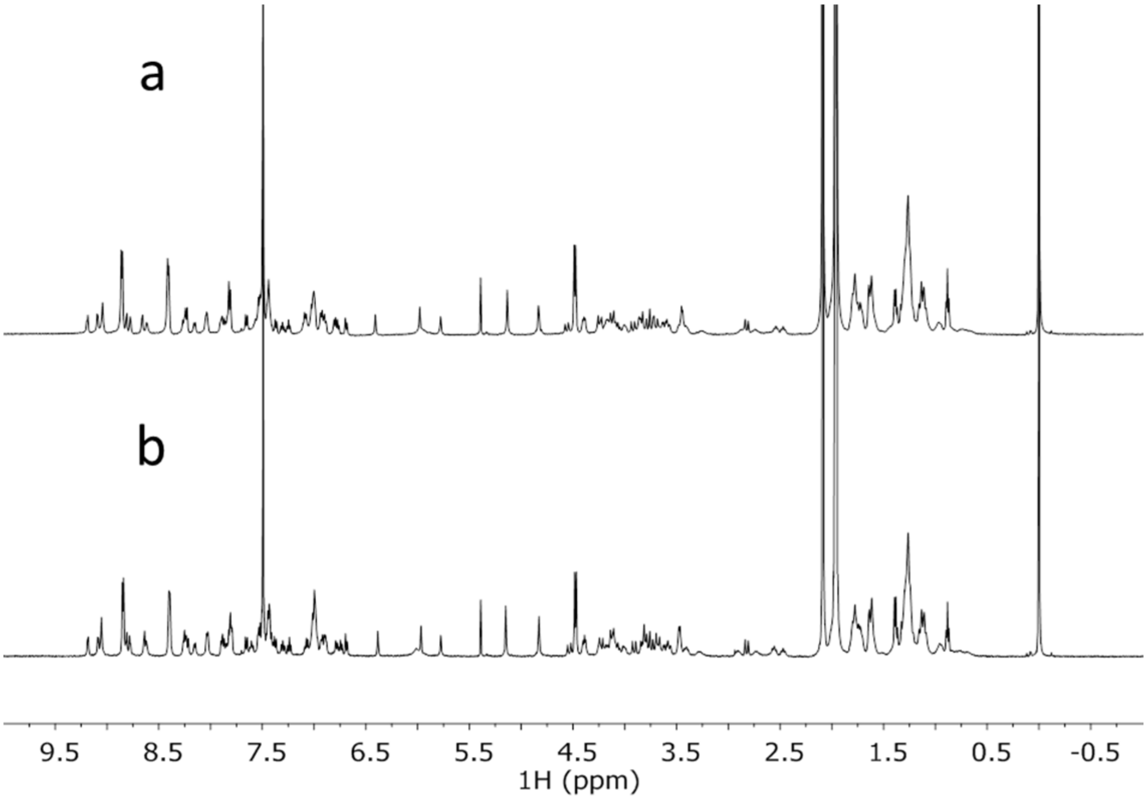

Supplementary Figure 10. NMR spectra of host-guest complexes. a 500 MHz ^{1}H NMR spectrum of **Zn2a·V14** (3 equivalents of **V14** with respect to **Zn2a**). **b** 500 MHz ^{1}H NMR spectrum of **Zn2b·V14** (3 equivalents of **V14** with respect to **Zn2b**) (c = 10^{-3} in $CDCl_3/CD_3CN$, 1:1, v/v, 298K).

Determination of PSS ratios

Samples of the motor + additives ($c_{motor} = 10^{-3}$ in $CDCl_3/CD_3CN$, 1:1, v/v, 277K) were irradiated inside the NMR spectrometer with λ_{max} = 365 nm light until the photostationary state was reached. The PSS ratios were obtained by integration of the signals corresponding to Me_{ax} (stable) and Me_{eq} (unstable).

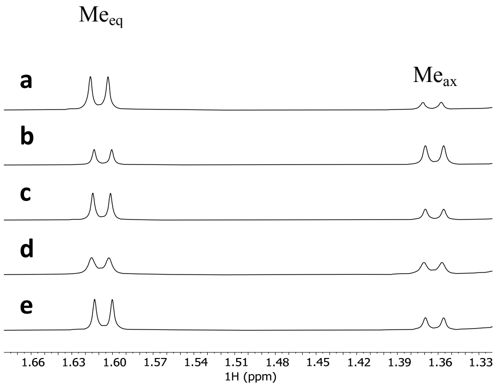

Supplementary Figure 11. Determination of PSS ratios. 500 MHz ^{1}H NMR spectra of PSS mixtures of (**a**) motor **8**, (**b**) motor **8** + **Zn1** (1 equivalent), (**c**) motor **8** + **Zn1** (1 equivalent) + **V12** (2 equivalents), (**d**) motor **8** + **Mn1** (1 equivalent) and (**e**) motor **8** + **Ni1** (1 equivalent); ($c = 10^{-3}$ in $CDCl_3/CD_3CN$, 1:1, v/v, 277K).

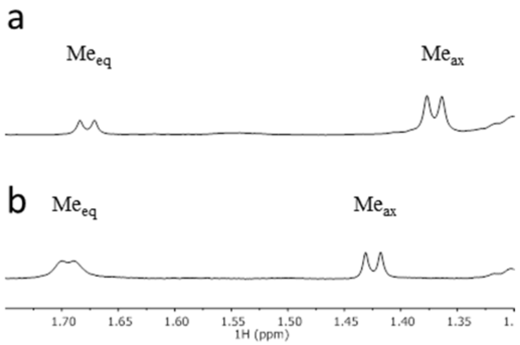

Supplementary Figure 12. Determination of PSS ratios. a 500 MHz ^{1}H NMR spectrum of PSS mixture of **Zn2a**. **b** 500 MHz ^{1}H NMR spectrum of PSS mixture of **Zn2a** + **V12** (2 equivalents); ($c = 10^{-3}$ in $CDCl_3/CD_3CN$, 1:1, v/v, 277K).

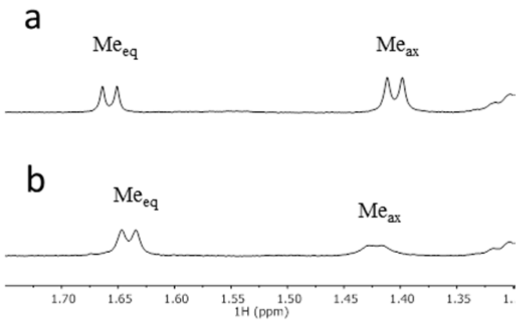

Supplementary Figure 13. Determination of PSS ratios. a 500 MHz ^{1}H NMR spectrum of PSS mixture of **Zn2b**. **b** 500 MHz ^{1}H NMR spectrum of PSS mixture of **Zn2b** + **V12** (2 equivalents); ($c = 10^{-3}$ in $CDCl_3/CD_3CN$, 1:1, v/v, 277K).

Structural changes of Zn2a and Zn2b upon irradiation

Upon irradiation of stable-**Zn2a**, structural changes were observed (Supplementary Figure 14). Due to the low PSS ratio of **Zn2a**, we could not fully characterize unstable-**Zn2a**. Nevertheless, we could observe a large shielding effect for protons 25 (doublet) and 26 (doublet of doublet) of unstable-**Zn2a** (circa 0.8 ppm). This indicates that the upper half of the molecular motor of unstable-**Zn2a** is folded towards the cavity of the cage compound, as was also observed for stable-**Zn2b**, i.e. the cavity entrance is “closed” by the molecular motor moiety. However, the shielding effect was not as big as that observed for protons 25 and 26 of compound stable-**Zn2b** (1.2–1.4 ppm), so the interaction was expected to be weaker.

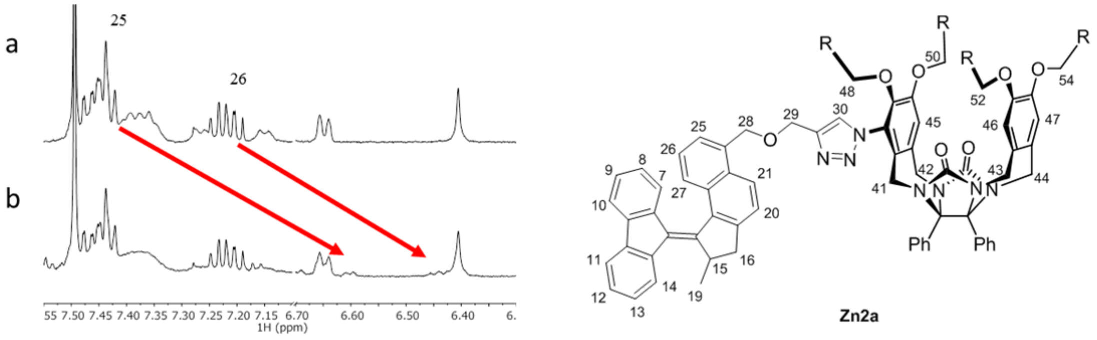

Supplementary Figure 8. Structural changes upon irradiation. a 500 MHz ^{1}H NMR spectrum of stable-**Zn2a**. **b** 500 MHz ^{1}H NMR spectuma of the PSS mixture of stable-**Zn2a** and unstable-**Zn2a** (c = 10^{-3} in $CDCl_3/CD_3CN$, 1:1, v/v, 297K).

In contrast to the shielding effect observed upon irradiating **Zn2a**, we observed a deshielding effect (circa 1.2 ppm) for the corresponding proton 26 (apparent triplet) upon irradiating **Zn2b** (Supplementary Figure 15). This indicates that the upper half of the molecular motor of unstable-**Zn2b** is no longer folded towards the cavity of the cage compound. Hence, we conclude that unstable-**Zn2b** has a geometry with an “open” cavity entrance.

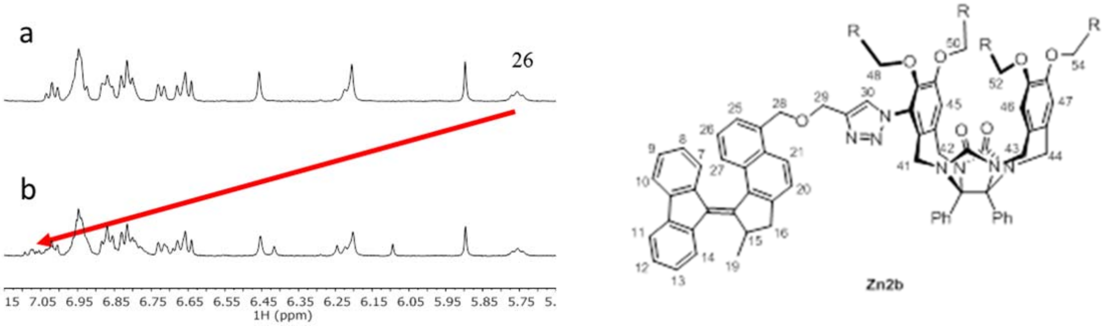

Supplementary Figure 9. Structural changes upon irradiation. a 500 MHz ^{1}H NMR spectrum of stable-**Zn2b**. **b** 500 MHz ^{1}H NMR spectrum of the PSS mixture of stable-**Zn2b** and unstable-**Zn2b** (c = 10^{-3} in $CDCl_3/CD_3CN$, 1:1, v/v, 297K).

Thermal helix inversion (THI) kinetics

Samples of the motor + additives ($c_{motor} = 10^{-3}$ in $CDCl_3/CD_3CN$, 1:1, v/v) were irradiated inside the NMR spectrometer with λ_{max} = 365 nm light until the photostationary state was reached. Then, the light was switched off and the mole fraction of the unstable intermediate was followed as a function of time. Methanol was used to calibrate the temperatures. The kinetic and thermodynamic parameters are summarized in Supplementary Table 3. Detailed kinetic data are provided in Supplementary Tables 4-6

Supplementary Table 3. Kinetic and thermodynamic parameters and Eyring plots for the thermal helix inversion of **8**, **Zn2a**, **Zn2b**, and their host–guest complexes with **V12**.

Motor	*k* (23.7 °C) ($\times 10^{-3}$ s^{-1})	*k* (18.7 °C) ($\times 10^{-3}$ s^{-1})	*k* (13.8 °C) ($\times 10^{-3}$ s^{-1})	*k* (8.8 °C) ($\times 10^{-3}$ s^{-1})	*k* (4.0 °C) $\times 10^{-3}$ (s^{-1})	$\Delta H^{\ddagger}$ (kJ·mol^{-1})	$\Delta S^{\ddagger}$ (J·mol^{-1}·K^{-1})	$\Delta G^{\ddagger}$ (20 °C) (kJ·mol^{-1})
**8**	2.27	1.48	0.87	0.47	0.25	79.6 ± 0.4	−25.9 ± 1.4	87.2 ± 0.8
**Zn2a**	1.47	0.86	0.49	0.26	0.14	79.6 ± 0.4	−30.5 ± 1.6	88.6 ± 0.9
**Zn2a·V12**	2.71	1.69	0.87	0.49	0.26	79.9 ± 0.6	−24.7 ± 2.1	87.1 ± 1.2
**Zn2b**	3.39	1.85	1.02	0.54	0.29	83.0 ± 0.3	−12.5 ± 1.2	86.7 ± 0.7
**Zn2b·V12**	3.07	1.78	0.94	0.50	0.26	83.9 ± 0.3	−10.1 ± 1.0	86.8 ± 0.6

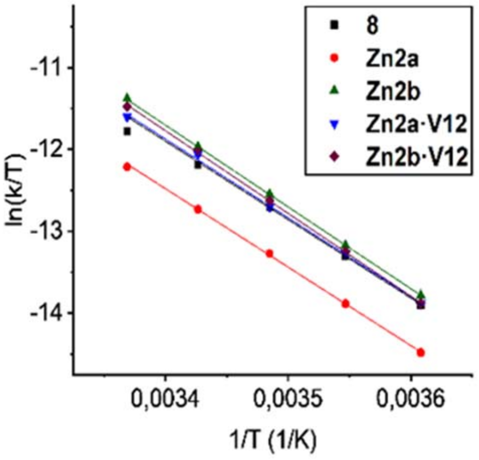

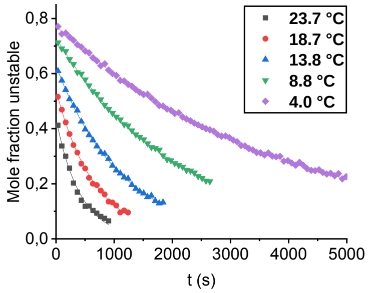

Supplementary Figure 10. Thermal helix inversions. First order kinetics plots for the thermal helix inversion of unstable-**8** to stable-**8** at five different temperatures. Mole fractions were determined by integration of the ^{1}H NMR signals corresponding to Me_{ax} (stable) and Me_{eq} (unstable).

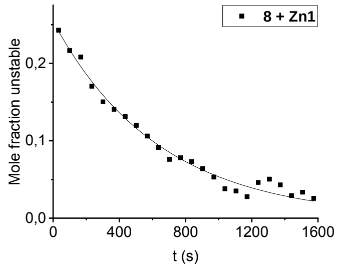

Supplementary Figure 17. Thermal helix inversions. First order kinetics plot for the thermal helix inversion of unstable-**8** to stable-**8** at 20 °C in the presence of a stoichiometric amount of **Zn1**. Mole fractions were determined by integration of the ^{1}H NMR signals corresponding to Me_{ax} (stable) and Me_{eq} (unstable).

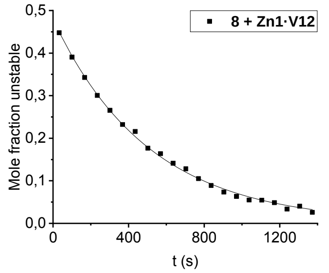

Supplementary Figure 18. Thermal helix inversions. First order kinetics plot for the thermal helix inversion of unstable-**8** to stable-**8** at 20 °C in the presence of a stoichiometric amount of host **Zn1** and an excess of guest **V12** (2 equivalents). Mole fractions were determined by integration of the ^{1}H NMR signals corresponding to Me_{ax} (stable) and Me_{eq} (unstable).

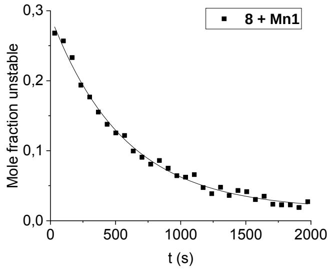

Supplementary Figure 19. Thermal helix inversions. First order kinetics plot for the thermal helix inversion of unstable-**8** to stable-**8** at 20 °C in the presence of a stoichiometric amount of **Mn1**. Mole fractions were determined by integration of the ^{1}H NMR signals corresponding to Me_{ax} (stable) and Me_{eq} (unstable).

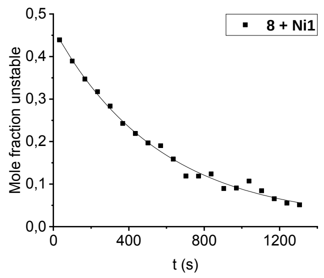

Supplementary Figure 20. Thermal helix inversions. First order kinetics plot for the thermal helix inversion of unstable-**8** to stable-**8** at 20 °C in the presence of a stoichiometric amount of **Ni1**. Mole fractions were determined by integration of the ^{1}H NMR signals corresponding to Me_{ax} (stable) and Me_{eq} (unstable).

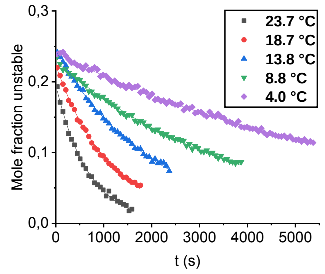

Supplementary Figure 21. Thermal helix inversions. First order kinetics plots for the thermal helix inversion of unstable-**Zn2a** to stable-**Zn2a** at five different temperatures. Mole fractions were determined by integration of the ^{1}H NMR signals corresponding to Me_{ax} (stable) and Me_{eq} (unstable).

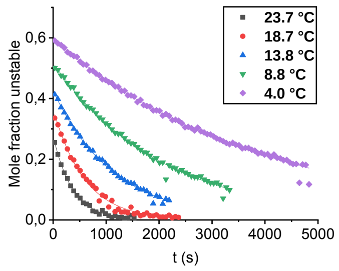

Supplementary Figure 22. Thermal helix inversions. First order kinetics plots for the thermal helix inversion of unstable-**Zn2a·V12** to stable-**Zn2a·V12** at five different temperatures. Guest **V12** was present in excess (2 equivalents). Mole fractions were determined by integration of the ^{1}H NMR signals corresponding to Me_{ax} (stable) and Me_{eq} (unstable).

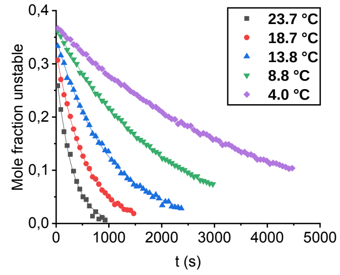

Supplementary Figure 23. Thermal helix inversions. First order kinetics plots for the thermal helix inversion of unstable-**Zn2b** to stable-**Zn2b** at five different temperatures. Mole fractions were determined by integration of the ^{1}H NMR signals corresponding to Me_{ax} (stable) and Me_{eq} (unstable).

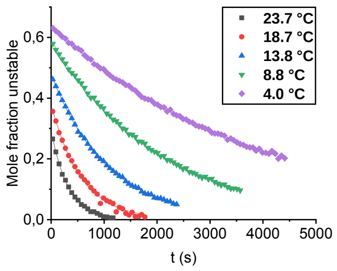

Supplementary Figure 24. Thermal helix inversions. First order kinetics plots for the thermal helix inversion of unstable-**Zn2b·V12** to stable-**Zn2b·V12** at five different temperatures. Guest **V12** was present in excess (2 equivalents). Mole fractions were determined by integration of the ^{1}H NMR signals corresponding to Me_{ax} (stable) and Me_{eq} (unstable).

Supplementary Table 4. Thermal helix inversions. Thermal helix inversion kinetics of motor **8** at different temperatures in the absence of additives and at 20 °C in the presence of additives. Mole fractions of unstable-**8** as a function of time were determined by integration of the ^{1}H NMR signals corresponding to Me_{ax} (stable) and Me_{eq} (unstable).

**Motor**	**8**					**8 + Zn1**	**8 + Zn1 + V12**	**8 + Mn1**	**8 + Ni1**
**Time (s)**	**23.7 °C**	**18.7 °C**	**13.8 °C**	**8.8 °C**	**4.0 °C**	**20 °C**	**20 °C**	**20 °C**	**20 °C**
33	0,41235	0,51589	0,60953	0,71328	0,77105	0,2428	0,44774	0,26798	0,4392
100	0,33723	0,46909	0,57533	0,69097	0,74434	0,21636	0,39054	0,25685	0,38931
167	0,29998	0,42298	0,54144	0,68103	0,74729	0,20823	0,34276	0,23305	0,34704
234	0,25671	0,3807	0,50752	0,65229	0,73303	0,17043	0,30057	0,1938	0,31723
301	0,20224	0,34075	0,48388	0,63398	0,72122	0,15029	0,26564	0,17666	0,28337
368	0,1694	0,31336	0,4663	0,60489	0,70572	0,14071	0,23219	0,15517	0,24263
435	0,13969	0,27304	0,42617	0,59908	0,69672	0,13115	0,21586	0,13775	0,21905
502	0,11778	0,25545	0,39796	0,57869	0,68216	0,12006	0,17674	0,12536	0,19702
569	0,11903	0,22124	0,37712	0,55703	0,6755	0,10609	0,16368	0,12173	0,19042
636	0,10286	0,19985	0,35856	0,53564	0,65741	0,0915	0,14142	0,09948	0,15897
703	0,09321	0,19478	0,33611	0,52591	0,64605	0,07597	0,12817	0,09065	0,11897
770	0,08132	0,17502	0,31442	0,50649	0,62883	0,07799	0,10514	0,08074	0,11886
837	0,07432	0,16109	0,30914	0,48595	0,63565	0,07299	0,08901	0,08601	0,12403
904	0,06497	0,13523	0,29073	0,47102	0,61297	0,06389	0,07365	0,07488	0,08965
971		0,13184	0,26584	0,45683	0,6002	0,05313	0,06352	0,0642	0,09072
1038		0,12101	0,24926	0,44307	0,59316	0,03805	0,05497	0,06212	0,10713
1105		0,09526	0,23782	0,43319	0,57544	0,03504	0,0546	0,06587	0,08443
1172		0,10286	0,22356	0,41567	0,57341	0,0278	0,04871	0,04722	0,06571
1239		0,09553	0,21866	0,41093	0,56105	0,04618	0,03377	0,0386	0,05554
1306			0,19602	0,39338	0,55399	0,05034	0,04054	0,0477	0,05134
1373			0,18244	0,37895	0,5423	0,04297	0,02602	0,03612	
1440			0,17208	0,3699	0,53231	0,02921		0,04322	
1507			0,16559	0,36123	0,52405	0,03353		0,04147	
1574			0,15197	0,34636	0,52501	0,02564		0,03024	
1641			0,15679	0,33247	0,51123			0,03494	
1708			0,13726	0,32878	0,49802			0,02342	
1775			0,12893	0,3234	0,48371			0,02264	
1842			0,13201	0,30325	0,48234			0,02271	
1909				0,28826	0,4714			0,01896	
1976				0,28453	0,46723			0,02715	
2043				0,27428	0,45559				
2110				0,26731	0,45813				
2177				0,26128	0,44241				
2244				0,24921	0,43488				
2311				0,24679	0,42898				
2378				0,2401	0,42004				
2445				0,22706	0,41347				
2512				0,22044	0,40677				
2579				0,21076	0,40008				
2646				0,20886	0,39608				
2713					0,39097				
2780					0,37834				
2847					0,3727				
2914					0,37368				
2981					0,36775				
3048					0,35844				

3115					0,35272				
3182					0,33895				
3249					0,33754				
3316					0,32789				
3383					0,32278				
3450					0,31355				
3517					0,3123				
3584					0,30432				
3651					0,31182				
3718					0,30058				
3785					0,29817				
3852					0,29966				
3919					0,28189				
3986					0,28413				
4053					0,27536				
4120					0,26736				
4187					0,27787				
4254					0,26678				
4321					0,25422				
4388					0,24843				
4455					0,24734				
4522					0,25059				
4589					0,24117				
4656					0,235				
4723					0,23796				
4790					0,22892				
4857					0,23662				
4924					0,21763				
4991					0,22573				

Supplementary Table 5. Thermal helix inversions. Thermal helix inversion kinetics of motor **Zn2a** at different temperatures in the absence and presence of **V12**. Mole fractions of unstable-**Zn2a** as a function of time were determined by integration of the ^{1}H NMR signals corresponding to Me_{ax} (stable) and Me_{eq} (unstable).

**Motor**	**Zn2a**					**Zn2a + V12**				
**Time (s)**	**23.7 °C**	**18.7 °C**	**13.8 °C**	**8.8 °C**	**4.0 °C**	**23.7 °C**	**18.7 °C**	**13.8 °C**	**8.8 °C**	**4.0 °C**
30	0,19298	0,22024	0,24167	0,23588	0,23887	0,25517	0,33562	0,41333	0,50111	0,59089
90	0,1711	0,20888	0,23525	0,22532	0,24032	0,21605	0,31361	0,39795	0,49428	0,58151
150	0,15796	0,19667	0,22805	0,21689	0,24199	0,1821	0,28237	0,37085	0,47584	0,57623
210	0,14231	0,18849	0,22295	0,21796	0,23675	0,15555	0,25994	0,35389	0,47014	0,5675
270	0,12681	0,1736	0,21146	0,21256	0,23175	0,13685	0,23381	0,33676	0,45143	0,55291
330	0,11872	0,16771	0,2072	0,20912	0,22669	0,11243	0,20572	0,31544	0,4377	0,54612
390	0,11078	0,16078	0,20163	0,20973	0,22673	0,09584	0,19776	0,29993	0,43721	0,53997
450	0,09873	0,14602	0,19446	0,20386	0,22319	0,08111	0,17626	0,28718	0,41629	0,53707
510	0,09101	0,14328	0,18958	0,20304	0,22201	0,07211	0,1626	0,27468	0,40082	0,52099
570	0,08255	0,13571	0,18872	0,19828	0,21798	0,05648	0,14588	0,25273	0,38817	0,51234
630	0,07781	0,13172	0,1765	0,19475	0,22297	0,05261	0,13355	0,2432	0,37459	0,51087
690	0,06717	0,12275	0,17316	0,18638	0,2165	0,04751	0,12415	0,23122	0,36941	0,49259
750	0,06574	0,11356	0,16719	0,18627	0,21725	0,02952	0,1131	0,22788	0,36396	0,49152
810	0,05713	0,10663	0,15969	0,18079	0,22056	0,02692	0,10361	0,20508	0,35214	0,482
870	0,05452	0,10386	0,15558	0,18075	0,21741	0,01843	0,06267	0,20051	0,33847	0,4765
930	0,05118	0,09656	0,15602	0,17796	0,20755	0,03143	0,08184	0,19796	0,33148	0,46326
990	0,04718	0,09539	0,1481	0,17904	0,20953	0,02096	0,07706	0,18657	0,3183	0,46131
1050	0,03858	0,08976	0,14474	0,17407	0,20854	0,01427	0,04256	0,17504	0,31308	0,45405
1110	0,04563	0,08462	0,14093	0,17118	0,2041	0,00899	0,06405	0,16181	0,29849	0,45099
1170	0,03537	0,08155	0,13374	0,16816	0,20013	0,01338	0,02746	0,15418	0,28704	0,4396
1230	0,03729	0,07808	0,13118	0,16625	0,20142	0,00873	0,03511	0,14358	0,28438	0,43872
1290	0,03146	0,07457	0,12922	0,16229	0,19732	0,00877	0,02581	0,13764	0,28374	0,42478
1350	0,02924	0,06956	0,12552	0,15637	0,19495	0,00575	0,02663	0,13032	0,26987	0,42546
1410	0,02698	0,06513	0,12122	0,15571	0,19509	0,00658	0,02398	0,12349	0,25913	0,41075
1470	0,02547	0,06501	0,11904	0,15139	0,19125	0,01525	0,04211	0,12138	0,25309	0,41016
1530	0,0175	0,06091	0,11673	0,15352	0,19546	0,00663	0,01975	0,11914	0,25077	0,40653
1590	0,02013	0,05791	0,11207	0,15284	0,19136		0,02354	0,10523	0,24038	0,38863
1650		0,05796	0,10612	0,14521	0,19281		0,01386	0,10036	0,23134	0,39529
1710		0,05352	0,10502	0,14275	0,19374		0,01763	0,09995	0,2235	0,37858
1770		0,05371	0,10511	0,14443	0,18469		0,02321	0,09498	0,22148	0,37543
1830			0,09757	0,14262	0,18714		0,01396	0,08424	0,21178	0,3733
1890			0,09443	0,13756	0,18512		0,01305	0,05411	0,19899	0,36043
1950			0,09384	0,13805	0,17867		0,0169	0,07951	0,2038	0,36127
2010			0,08709	0,13242	0,18298		0,01004	0,07568	0,18856	0,35939
2070			0,09119	0,13064	0,17404		0,01016	0,05325	0,18957	0,34348

2130			0,08481	0,13259	0,1759		0,00989	0,06913	0,13372	0,34902
2190			0,08679	0,12761	0,1727		0,01579	0,06475	0,1805	0,34301
2250			0,08574	0,12442	0,17355		0,00676		0,17299	0,32883
2310			0,08141	0,12594	0,16916		0,01085		0,17053	0,33018
2370			0,07393	0,12179	0,17265		0,00847		0,1607	0,32693
2430				0,12166	0,16966				0,16467	0,31852
2490				0,11791	0,16785				0,15659	0,30536
2550				0,11915	0,16699				0,15178	0,30681
2610				0,1175	0,16905				0,14442	0,3026
2670				0,11679	0,15992				0,14183	0,29492
2730				0,1092	0,16346				0,14124	0,30431
2790				0,11204	0,16203				0,14402	0,29199
2850				0,1046	0,16432				0,1338	0,28819
2910				0,10942	0,15566				0,12815	0,28249
2970				0,10613	0,1572				0,12249	0,28156
3030				0,10159	0,15717				0,12122	0,27512
3090				0,10109	0,15383				0,12339	0,26834
3150				0,10312	0,15111				0,1161	0,26478
3210				0,09911	0,15746				0,07119	0,25926
3270				0,09995	0,15216				0,10788	0,25367
3330				0,09603	0,15056				0,09891	0,2492
3390				0,09216	0,15462					0,254
3450				0,09551	0,15038					0,24789
3510				0,09013	0,14855					0,24613
3570				0,09068	0,14361					0,23568
3630				0,08756	0,14363					0,23726
3690				0,08659	0,14125					0,23699
3750				0,08499	0,1393					0,23088
3810				0,08708	0,14407					0,22807
3870				0,08681	0,13637					0,2188
3930					0,13536					0,22159
3990					0,13641					0,21497
4050					0,13406					0,217
4110					0,13369					0,20609
4170					0,1335					0,21551
4230					0,13					0,20443
4290					0,13409					0,20317
4350					0,12802					0,19648
4410					0,1248					0,19782
4470					0,12764					0,18909
4530					0,12562					0,18397
4590					0,12418					0,18451
4650					0,12309					0,12234
4710					0,12257					0,18263
4770					0,12369					0,1813
4830					0,12041					0,11729
4890					0,12286					
4950					0,11852					
5010					0,1178					
5070					0,11825					
5130					0,11546					
5190					0,11869					
5250					0,11419					
5310					0,11576					
5370					0,11404					

Supplementary Table 6. Thermal helic inversions. Thermal helix inversion kinetics of motor **Zn2b** at different temperatures in the absence and presence of **V12**. Mole fractions of unstable-**Zn2b** as a function of time were determined by integration of the ^{1}H NMR signals corresponding to Me_{ax} (stable) and Me_{eq} (unstable).

**Motor**	**Zn2b**					**Zn2b + V12**				
**Time (s)**	**23.7 °C**	**18.7 °C**	**13.8 °C**	**8.8 °C**	**4.0 °C**	**23.7 °C**	**18.7 °C**	**13.8 °C**	**8.8 °C**	**4.0 °C**
30	0,25886	0,30694	0,33353	0,36162	0,36679	0,26577	0,35623	0,46181	0,57898	0,63108
90	0,2142	0,27064	0,31604	0,35198	0,3623	0,22284	0,32483	0,43894	0,55934	0,621
150	0,17243	0,24199	0,30169	0,33928	0,3555	0,18355	0,28617	0,41444	0,54422	0,61273
210	0,14642	0,22505	0,27359	0,32644	0,34794	0,14901	0,26148	0,39308	0,52829	0,60186
270	0,13012	0,20129	0,26481	0,31768	0,34585	0,12991	0,23527	0,37246	0,51358	0,59605
330	0,09853	0,17746	0,2511	0,30834	0,33637	0,10749	0,2109	0,35219	0,49495	0,58218
390	0,07912	0,15959	0,23607	0,2999	0,33404	0,08888	0,19369	0,33393	0,48284	0,57882
450	0,06469	0,14269	0,22132	0,28352	0,32628	0,07445	0,1743	0,31262	0,47421	0,56618
510	0,05019	0,12657	0,20877	0,27999	0,31963	0,06379	0,1575	0,28913	0,45931	0,56285

570	0,04503	0,11243	0,19737	0,27168	0,31789	0,04983	0,14328	0,27722	0,44882	0,5561
630	0,03303	0,1032	0,18498	0,26449	0,30903	0,0442	0,13108	0,26717	0,43414	0,54137
690	0,01395	0,08767	0,16865	0,25479	0,30243	0,03552	0,12115	0,24968	0,41538	0,53228
750	0,02225	0,0837	0,16379	0,24424	0,3004	0,02607	0,10539	0,23398	0,40111	0,5274
810	0,00849	0,07394	0,15179	0,24125	0,29267	0,02616	0,09422	0,22286	0,40141	0,51202
870	0,01142	0,06286	0,15012	0,23081	0,29075	0,02042	0,08422	0,2085	0,3859	0,50725
930	0,0065	0,05779	0,13769	0,22744	0,28234	0,01497	0,05304	0,19945	0,37364	0,49819
990		0,05118	0,13536	0,2175	0,27759	0,00951	0,07114	0,19285	0,36668	0,49547
1050		0,04453	0,11966	0,21259	0,27311	0,0085	0,06254	0,17912	0,3496	0,48417
1110		0,03567	0,11442	0,20313	0,26993	0,006	0,05823	0,16937	0,34262	0,47518
1170		0,03781	0,10705	0,19917	0,26709	0,00616	0,05284	0,16029	0,32958	0,4666
1230		0,03305	0,09436	0,19444	0,2608		0,02561	0,15188	0,32436	0,46138
1290		0,02665	0,09383	0,18819	0,25992		0,04069	0,14368	0,31513	0,45642
1350		0,02548	0,08489	0,17891	0,25426		0,03558	0,13993	0,29972	0,45197
1410		0,02488	0,08177	0,17358	0,24872		0,02089	0,12645	0,29649	0,44305
1470		0,01891	0,07313	0,16906	0,2454		0,01527	0,11784	0,28073	0,44059
1530			0,06956	0,16093	0,24163		0,02426	0,1115	0,28	0,43068
1590			0,0704	0,15742	0,23613		0,01007	0,10744	0,27247	0,42491
1650			0,06518	0,15411	0,23227		0,01016	0,10418	0,25708	0,41431
1710			0,05989	0,14813	0,2291		0,01662	0,09268	0,256	0,40398
1770			0,05735	0,14529	0,22635		0,00846	0,08593	0,25021	0,39866
1830			0,04955	0,13812	0,21993			0,08364	0,23682	0,39569
1890			0,05389	0,13462	0,21885			0,08263	0,22971	0,39316
1950			0,0472	0,13163	0,21362			0,07281	0,22368	0,3822
2010			0,03423	0,12274	0,20909			0,07018	0,21967	0,37862
2070			0,04106	0,12048	0,20348			0,07	0,20847	0,37081
2130			0,04109	0,11729	0,20078			0,0658	0,20963	0,36958
2190			0,0318	0,11375	0,19648			0,06025	0,19671	0,35617
2250			0,03082	0,1092	0,19572			0,05944	0,19552	0,36192
2310			0,03011	0,10653	0,18707			0,05225	0,18886	0,34821
2370			0,02865	0,10255	0,19065			0,05022	0,18143	0,34307
2430				0,1006	0,1853				0,17664	0,34056
2490				0,09606	0,18311				0,16685	0,33141
2550				0,0926	0,18342				0,16947	0,32977
2610				0,08773	0,1757				0,15717	0,32191
2670				0,08912	0,17258				0,15448	0,31894
2730				0,08137	0,16719				0,15077	0,3101
2790				0,08002	0,16973				0,14324	0,307
2850				0,07801	0,1673				0,13825	0,30524
2910				0,07519	0,16373				0,14176	0,30006
2970				0,074	0,15768				0,13352	0,29695
3030					0,15817				0,13474	0,28633
3090					0,15504				0,1238	0,28371
3150					0,14774				0,11877	0,28316
3210					0,14892				0,11357	0,28012
3270					0,14853				0,11626	0,2766
3330					0,14501				0,1075	0,26276
3390					0,14078				0,10235	0,26732
3450					0,13766				0,10312	0,26118
3510					0,1338				0,10077	0,25418
3570					0,13449				0,09669	0,24879
3630					0,12966					0,24611
3690					0,12971					0,23962
3750					0,12659					0,23785
3810					0,12039					0,23754
3870					0,12038					0,2272
3930					0,11991					0,23325
3990					0,11776					0,22073
4050					0,11236					0,22014
4110					0,11397					0,21706
4170					0,11275					0,22602
4230					0,11042					0,21732
4290					0,10793					0,20073
4350					0,10592					0,21065
4410					0,10308					0,20209
4470					0,10388					

UV-vis and fluorescence spectra

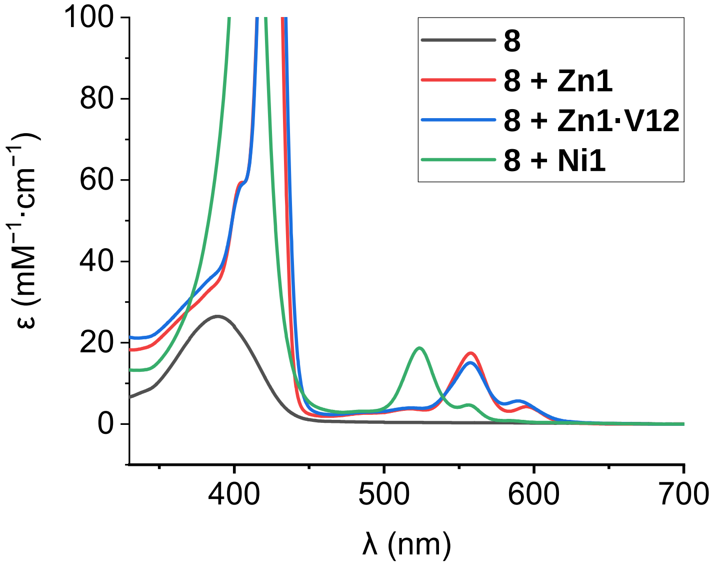

Supplementary Figure 11. UV-Vis absorption spectra. UV-vis absorption spectra of motor **8** (grey) and of motor **8** in the presence of porphyrin cage compounds **Zn1** (red, 1 equivalent), **Zn1** (1 equivalent) and **V12** (blue, 2 equivalents), and **Ni1** (green, 1 equivalent). Spectra were recorded at 298 K ($c_{motor} = 10^{-5}$ M in $CHCl_3/CH_3CN$, 1:1, v/v).

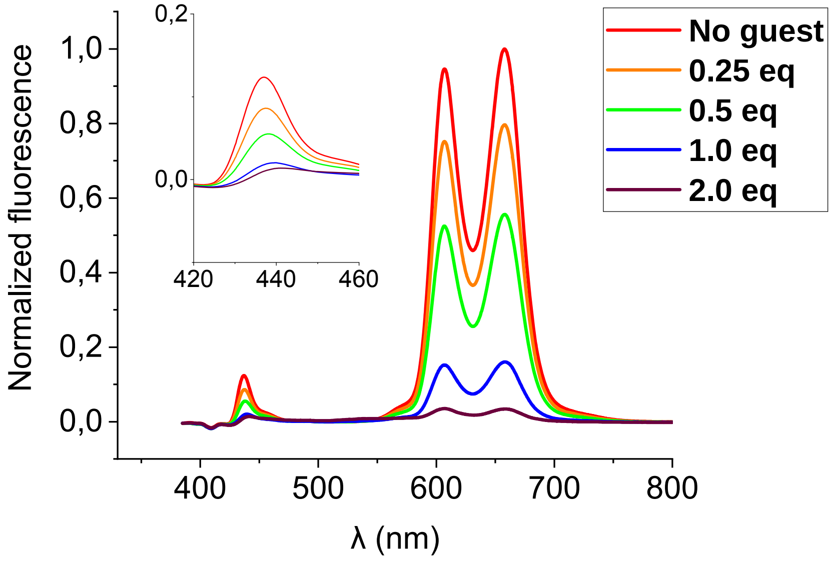

Supplementary Figure 26. Fluorescence spectra. Normalized fluorescence spectra of porphyrin cage compound **Zn1** in the absence and presence of viologen **V12**. Spectra were recorded at 298 K (λ_{ex} = 365 nm; c = 10^{-5} M in $CHCl_3/CH_3CN$, 1:1, v/v).

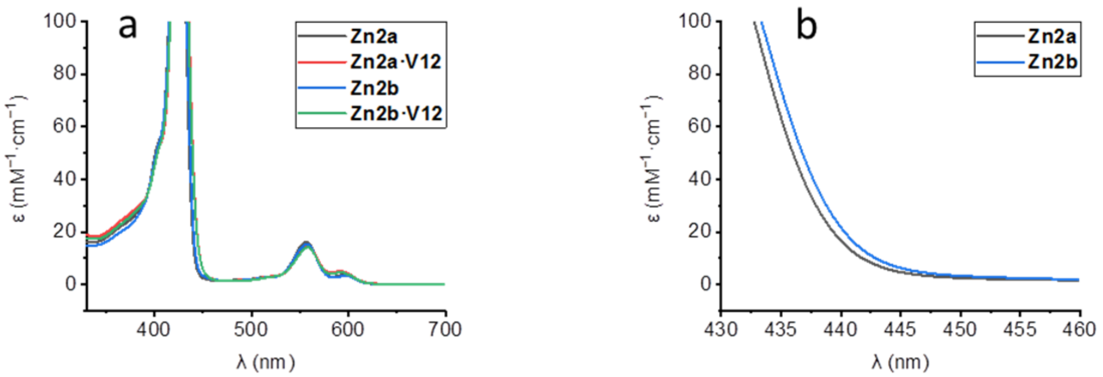

Supplementary Figure 27. UV-Vis absorption spectra. a UV-vis absorption spectra of **Zn2a** (grey), **Zn2a** in the presence of **V12** (red, 2 equivalents), **Zn2b** (blue), and **Zn2b** in the presence of **V12** (green, 2 equivalents). **b** Zoom of the UV-vis absorption spectra of **Zn2a** (grey) and **Zn2b** (blue) in the 430–460 nm region. Spectra were recorded at 298 K ($c_{cage} = 10^{-5}$ M in $CHCl_3/CH_3CN$, 1:1, v/v).

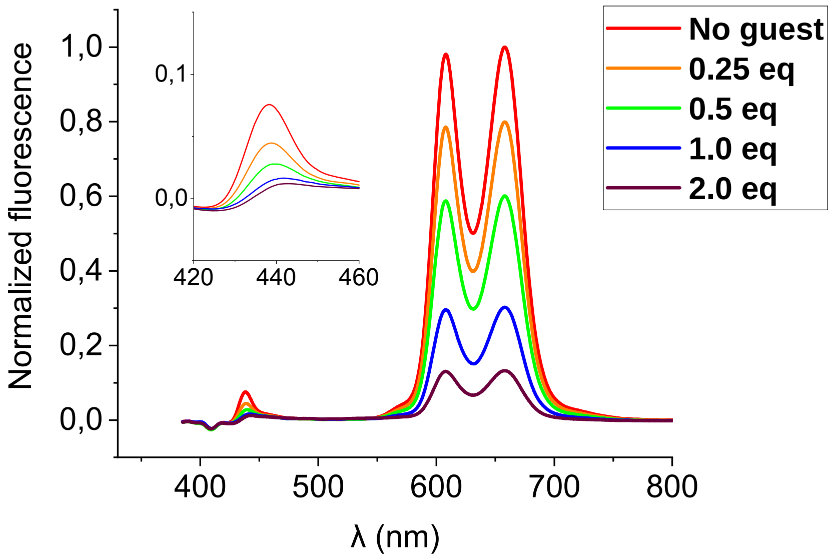

Supplementary Figure 28. Fluorescence spectra. Fig. S12. Normalized fluorescence spectra of porphyrin cage compound **Zn2a** in the absence and presence of viologen **V12**. Spectra were recorded at 298 K (λ_{ex} = 365 nm; c = 10^{-5} M in $CHCl_3/CH_3CN$, 1:1, v/v).

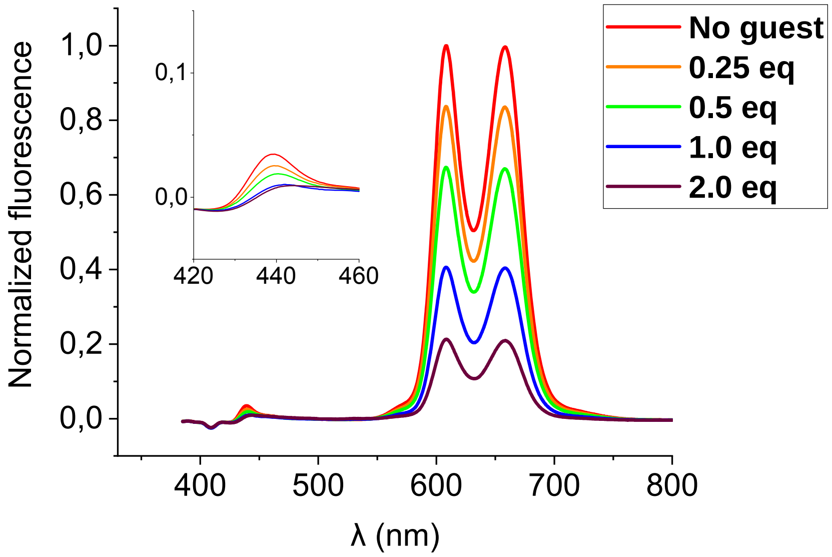

Supplementary Figure 29. Fluorescence spectra. Normalized fluorescence spectra of porphyrin cage compound **Zn2b** in the absence and presence of viologen **V12**. Spectra were recorded at 298 K (λ_{ex} = 365 nm; c = 10^{-5} M in $CHCl_3$/CH_3CN, 1:1, v/v).

Threading data

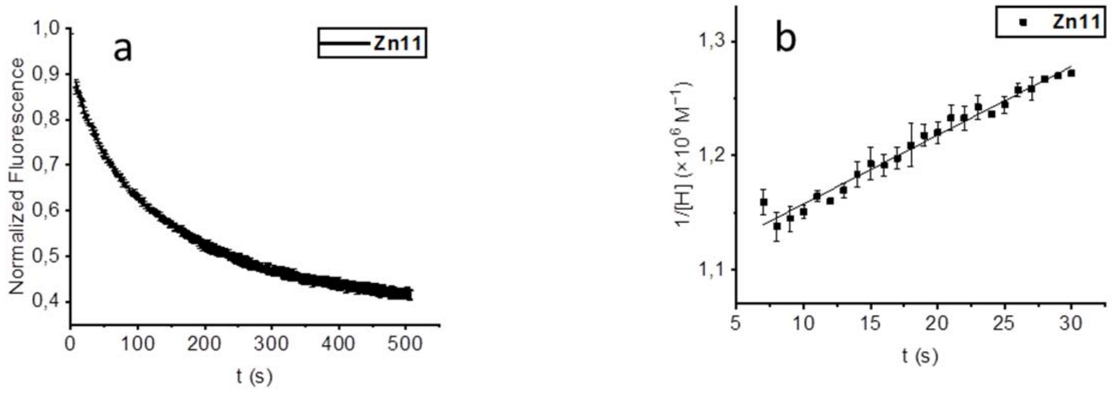

Supplementary Figure 30. Threading as followed by UV-Vis. a Observed changes in fluorescence intensity upon threading **V15** through **Zn11**. (left) Normalized fluorescence intensity of the host measured as a function of time, after the addition of 1.0 equivalent of **V15** ($[host]_0 = [\mathbf{V15}]_0 = 10^{-6}$ M in $CHCl_3/CH_3CN$, 1:1, v/v). **b** Second-order kinetics plots for the complexation of host **Zn11** with **V15** for the initial stages of the threading process. Error bars = standard deviation (n = 3).

Supplementary Table 7. Threading data. Kinetic data for the threading of **V15** through different hosts. Normalized fluorescence intensities of hosts as a function of time, after the addition of 1.0 equivalent of **V15** ($[host]_0 = [\mathbf{V15}]_0 = 10^{-6}$ M in $CHCl_3/CH_3CN$, 1:1, v/v). The average normalized fluorescence intensities and standard deviations are based on triplo experiments. The normalization factor was determined by taking the average fluorescence intensity of a solution of pure host ($[host] = 10^{-6}$ M in $CHCl_3/CH_3CN$, 1:1, v/v).

	Zn2a		Zn2a (PSS)		Zn2b		Zn2b (PSS)		Zn11	
**Time (s)**	**Average**	**Stdev**	**Average**	**Stdev**	**Average**	**Stdev**	**Average**	**Stdev**	**Average**	**Stdev**
0	1	0,01482	1	0,01389	1	0,01407	1	0,01585	1	0,01069
1										
2										
3										
4										
5										
6										
7									0,86283	0,00822
8	0,90919	0,00976	0,90108	0,01704	0,92494	0,01759	0,90577	0,00954	0,87882	0,00981
9	0,90578	0,00312	0,88762	0,01769	0,93119	0,01035	0,90103	0,0085	0,87354	0,00851
10	0,89334	0,00691	0,88894	0,0263	0,92637	0,01269	0,89703	0,01213	0,86914	0,00464
11	0,89741	0,00658	0,88794	0,02205	0,9211	0,00158	0,8888	0,00957	0,85918	0,00347
12	0,88863	7,58234E-4	0,88269	0,02082	0,91768	0,00759	0,88565	0,00596	0,86225	7,65155E-4
13	0,88195	0,00323	0,87096	0,02414	0,91334	0,0101	0,8862	0,00834	0,85522	0,00458
14	0,87763	0,00338	0,87157	0,01481	0,90717	0,0075	0,88255	0,00851	0,84519	0,00773
15	0,87051	0,00672	0,86368	0,02122	0,90157	0,00513	0,8776	0,0077	0,83852	0,00995
16	0,8645	0,00206	0,8569	0,02084	0,89389	0,0083	0,87406	0,00618	0,83944	0,00648
17	0,8576	0,00258	0,85874	0,01633	0,8935	0,01119	0,8684	0,01031	0,83504	0,00665
18	0,85105	0,00168	0,85187	0,01495	0,89031	0,01143	0,86349	0,01246	0,82715	0,01294
19	0,84721	0,00571	0,84808	0,01681	0,89208	0,00539	0,85853	0,01042	0,82127	0,00635
20	0,83987	0,00278	0,84507	0,01818	0,88504	0,00901	0,85506	0,01081	0,81973	0,00665
21	0,83801	0,0065	0,84048	0,01883	0,88084	0,01164	0,85301	0,00702	0,81116	0,00712
22	0,82965	0,0075	0,83624	0,02025	0,87899	0,01572	0,84338	0,00698	0,81119	0,00691
23	0,82396	0,00348	0,83497	0,01643	0,87441	0,01349	0,84295	0,00505	0,80497	0,00678
24	0,81871	0,00449	0,83116	0,01294	0,86972	0,01297	0,84168	0,00693	0,8089	0,00118
25	0,8185	5,14834E-4	0,83031	0,01268	0,86899	0,01002	0,83617	0,00552	0,80369	0,00483
26	0,81272	0,00153	0,82301	0,01416	0,86817	0,01621	0,83424	0,00702	0,79533	0,00391
27	0,80736	0,00229	0,81829	0,01373	0,86329	0,00881	0,83407	0,00662	0,79464	0,00661
28	0,79929	0,00562	0,81237	0,01564	0,86302	0,00579	0,83329	9,08502E-4	0,78915	0,00166
29	0,80317	7,79061E-4	0,80703	0,01776	0,85422	0,00583	0,82421	0,00996	0,7876	0,00129
30	0,79742	0,00427	0,80485	0,01404	0,85069	0,00397	0,82037	0,00678	0,78624	0,00129

31	0,7944	0,0046	0,80205	0,01185	0,84904	0,00635	0,81998	0,00447	0,78441	0,008
32	0,78816	0,00729	0,80211	0,01096	0,84057	0,0021	0,81491	0,0047	0,78197	0,00529
33	0,78729	0,00305	0,79539	0,01524	0,84295	0,00355	0,81237	0,00685	0,77667	0,00703
34	0,77915	0,00416	0,79352	0,01508	0,83704	0,00108	0,81604	0,00763	0,77253	0,00489
35	0,77956	0,00406	0,78827	0,01241	0,83211	0,00426	0,81116	0,00819	0,77338	0,00753
36	0,77174	0,00367	0,78662	0,01221	0,83014	0,00674	0,81147	0,004	0,77011	0,00599
37	0,77601	0,0029	0,78059	0,01522	0,82842	0,00305	0,80369	0,00489	0,76939	0,00764
38	0,76981	0,00378	0,782	0,01497	0,82574	0,0033	0,80292	0,00225	0,76184	0,00616
39	0,76234	0,00356	0,7794	0,02101	0,82411	8,64114E-4	0,8033	0,00833	0,75699	0,008
40	0,76194	0,00264	0,77552	0,01378	0,82302	0,0025	0,79821	0,00664	0,75394	0,00368
41	0,7591	0,00512	0,77037	0,01584	0,8221	0,00109	0,79629	9,45363E-4	0,75185	0,00564
42	0,75739	0,00544	0,7697	0,01774	0,81794	0,00172	0,79618	0,00845	0,74486	0,00383
43	0,75815	0,0046	0,76755	0,01728	0,81902	0,00201	0,7935	0,0069	0,74186	0,00469
44	0,75427	0,00314	0,76441	0,01657	0,81011	0,00427	0,79093	0,00431	0,74215	0,0062
45	0,75513	0,00361	0,76033	0,0172	0,80881	0,00146	0,78825	0,007	0,73419	0,00858
46	0,75172	0,00259	0,76092	0,0171	0,80763	3,60277E-4	0,78909	0,0035	0,7346	0,00793
47	0,74774	0,00278	0,75738	0,01368	0,80175	0,0081	0,78782	0,00297	0,73045	0,00451
48	0,74363	0,00211	0,75201	0,02177	0,80383	0,00296	0,78921	0,00114	0,72696	0,00689
49	0,7459	0,00543	0,75275	0,02094	0,7995	0,00559	0,78475	0,00541	0,72536	0,00856
50	0,74221	0,00583	0,75234	0,01909	0,79604	0,00468	0,78138	0,00488	0,72208	0,00616
51	0,73817	0,00407	0,74958	0,02026	0,79354	0,00691	0,78412	0,00533	0,72245	0,00884
52	0,73649	0,00329	0,74869	0,01705	0,79167	0,00646	0,78349	6,93587E-4	0,71885	0,00718
53	0,73753	1,06077E-4	0,74655	0,02092	0,79388	0,00562	0,77952	0,0036	0,71955	0,00394
54	0,72964	0,00215	0,7423	0,01933	0,78998	0,00376	0,77477	0,00255	0,71029	0,00656
55	0,73416	0,00189	0,73841	0,02363	0,78795	0,00556	0,77825	0,00496	0,7092	0,00467
56	0,73089	0,00373	0,73672	0,0219	0,79038	0,00514	0,76946	0,00786	0,70739	4,43095E-4
57	0,73189	0,00429	0,73572	0,02317	0,78914	0,00636	0,77348	0,00312	0,70647	0,00293
58	0,72916	0,00487	0,72939	0,02424	0,79099	0,00234	0,76696	0,00473	0,70713	0,00648
59	0,72605	0,00594	0,73019	0,02325	0,79031	0,00198	0,77097	0,00589	0,7051	0,00325
60	0,72822	0,00684	0,7263	0,02077	0,79585	0,00295	0,76871	0,00723	0,70162	0,00231
61	0,72575	0,00275	0,72612	0,02257	0,78331	0,00818	0,76744	0,00146	0,70111	0,00173
62	0,72626	0,00449	0,7246	0,02391	0,78667	0,00428	0,76223	0,00787	0,69498	0,00121
63	0,71977	0,00453	0,7251	0,01947	0,78546	0,00247	0,76884	0,0056	0,69494	0,00164
64	0,72265	0,00771	0,72085	0,02268	0,7863	0,00112	0,76236	0,00445	0,69174	0,00466
65	0,72175	0,00479	0,72063	0,02227	0,78046	0,00351	0,76487	0,00242	0,68823	0,00147
66	0,721	0,00488	0,72169	0,0238	0,7827	0,00574	0,76184	0,00154	0,68489	0,00151
67	0,71732	0,00761	0,71811	0,0193	0,78056	0,00329	0,7621	0,00259	0,68572	0,00564
68	0,71716	0,01333	0,71985	0,01999	0,77788	0,00884	0,75894	0,00119	0,68191	0,00379
69	0,71411	0,00485	0,71596	0,02037	0,78136	0,00892	0,75919	0,00575	0,68152	0,00478
70	0,71196	0,00743	0,71502	0,01856	0,77478	0,00576	0,75695	0,00558	0,67882	0,00674
71	0,70812	0,01015	0,71569	0,02214	0,77805	0,00629	0,75993	0,00373	0,67989	0,00191
72	0,71343	0,0106	0,71166	0,01882	0,76968	0,00615	0,75845	0,00326	0,675	0,00128
73	0,70564	0,00969	0,71313	0,02287	0,77638	0,0064	0,75404	0,00707	0,67236	0,00615
74	0,7032	0,01027	0,71121	0,01658	0,76936	0,00546	0,75146	0,00227	0,67152	0,00551
75	0,69971	0,01379	0,71075	0,01959	0,76993	0,00937	0,75287	0,0064	0,67421	0,00667
76	0,70051	0,01008	0,70502	0,02093	0,76975	0,0056	0,75148	0,00354	0,66987	0,00188
77	0,69758	0,00939	0,71044	0,0205	0,76967	0,00672	0,75218	0,00154	0,67305	0,00103
78	0,69721	0,00832	0,70703	0,02016	0,769	0,00515	0,75211	0,00139	0,65798	0,00556
79	0,69724	0,01037	0,70509	0,01731	0,76464	0,00313	0,75068	0,00257	0,65709	0,00454
80	0,69547	0,00812	0,70146	0,0172	0,76006	0,00469	0,7477	0,00803	0,66236	0,00443
81	0,69397	0,00895	0,70101	0,02084	0,75775	0,00282	0,75023	0,00348	0,66111	0,00232
82	0,69008	0,01229	0,70022	0,02144	0,76268	0,00731	0,74126	0,00561	0,65689	0,00541
83	0,69228	0,00944	0,70392	0,01793	0,76011	0,00447	0,74216	0,002	0,65685	0,00463
84	0,69217	0,00626	0,70141	0,02145	0,75743	0,00318	0,74725	0,0038	0,65453	8,46598E-4
85	0,69276	0,00713	0,69989	0,01992	0,75418	0,00345	0,7461	0,00448	0,65245	0,00215
86	0,69067	0,00648	0,69808	0,01562	0,75411	0,00189	0,74405	0,00385	0,64664	0,00135
87	0,68693	0,0065	0,69856	0,01671	0,7545	0,00669	0,7444	0,00453	0,64723	0,00248
88	0,68793	0,00715	0,6944	0,02112	0,7572	0,00663	0,73613	0,00508	0,64461	0,0055
89	0,686	0,00519	0,69403	0,01511	0,75241	0,0035	0,74102	0,0085	0,64584	0,00343
90	0,67957	0,00251	0,6967	0,02071	0,75138	0,00723	0,74054	0,0017	0,64051	0,00122
91	0,68269	0,00444	0,68903	0,0224	0,75419	0,00622	0,73727	0,00176	0,63876	0,00357
92	0,68286	0,00765	0,69121	0,01886	0,7526	0,0022	0,7364	0,00339	0,63894	0,00507
93	0,68187	0,00657	0,6896	0,01987	0,75014	0,00564	0,73644	0,00265	0,63672	0,00519
94	0,67791	0,00429	0,68957	0,01544	0,74814	0,00987	0,73556	6,69698E-4	0,63564	0,0018
95	0,68053	0,00393	0,687	0,02015	0,74865	0,00752	0,73969	0,00338	0,64127	0,00466
96	0,68062	0,00435	0,68855	0,02049	0,75169	0,00859	0,73642	0,00513	0,6365	0,00364
97	0,68086	0,00422	0,68571	0,0204	0,74927	0,0098	0,73945	0,00275	0,63302	0,0048
98	0,67815	0,00679	0,68452	0,0196	0,74874	0,00875	0,73351	0,0025	0,63297	0,00502
99	0,68014	0,00622	0,68717	0,02149	0,74647	0,01019	0,73436	0,00394	0,63108	0,0056
100	0,67551	0,00448	0,68607	0,02091	0,74853	0,01163	0,73203	0,00248	0,62965	0,00245
101	0,67942	0,00786	0,68494	0,02008	0,74319	0,00776	0,73209	0,00531	0,62764	0,00557
102	0,67742	0,0047	0,68242	0,01747	0,74186	0,00541	0,73552	0,00283	0,62352	0,00127
103	0,67538	0,01142	0,68579	0,02075	0,74094	0,00882	0,7337	0,00859	0,62895	0,00425
104	0,6694	0,00976	0,68047	0,01545	0,74056	0,00602	0,72987	0,0061	0,62501	0,00364

105	0,66992	0,00684	0,68166	0,01718	0,73879	0,00784	0,73351	0,00616	0,62278	0,00631
106	0,66748	0,0082	0,68255	0,01687	0,73806	0,00646	0,72947	0,00386	0,62136	0,00265
107	0,671	0,01176	0,68004	0,01573	0,73642	0,00498	0,73091	0,00362	0,61762	0,00849
108	0,66831	0,00889	0,68121	0,0198	0,73762	0,00662	0,72758	0,00488	0,62204	0,00697
109	0,6676	0,00981	0,6801	0,01811	0,73575	0,00819	0,7264	0,00378	0,61714	0,00387
110	0,66753	0,01154	0,68375	0,02323	0,73605	0,00591	0,72637	0,00537	0,61712	0,00343
111	0,66441	0,00844	0,68225	0,01977	0,73457	0,00537	0,72798	0,00555	0,61257	0,00236
112	0,66186	0,00736	0,68201	0,01783	0,73494	0,00703	0,72469	0,00747	0,61002	0,00529
113	0,66761	0,00692	0,67901	0,01566	0,73442	0,00646	0,72381	0,00306	0,60833	0,00513
114	0,66025	0,00566	0,67779	0,01961	0,73404	0,00872	0,72328	0,00699	0,61062	9,41974E-4
115	0,66279	0,00958	0,67842	0,01834	0,73197	0,00954	0,72476	0,00636	0,60473	0,00691
116	0,66277	0,01188	0,67448	0,01781	0,72948	0,00423	0,72537	0,00589	0,60737	0,00739
117	0,66705	0,00415	0,67624	0,01671	0,731	0,00821	0,72214	0,00606	0,60618	0,00582
118	0,66222	0,00817	0,67598	0,01959	0,73022	0,0064	0,72404	0,00519	0,60454	0,00294
119	0,66342	0,00786	0,67162	0,01991	0,72944	0,00625	0,72145	0,00487	0,60257	0,00172
120	0,6654	0,00521	0,67183	0,01928	0,73308	0,00612	0,72049	0,00973	0,59823	0,00547
121	0,66283	0,00983	0,67145	0,01823	0,72982	0,00611	0,72211	0,00538	0,6011	0,00388
122	0,66344	0,01104	0,67047	0,01998	0,73283	0,00373	0,7177	0,00657	0,60229	0,00444
123	0,66489	0,0056	0,66794	0,02238	0,72943	0,00336	0,71711	0,00295	0,59958	0,00116
124	0,66259	0,0053	0,67101	0,02049	0,73015	0,00473	0,72272	0,00427	0,60034	0,00356
125	0,66171	0,00866	0,66996	0,01846	0,73112	0,0039	0,72243	0,0037	0,60098	0,00325
126	0,66114	0,0066	0,66725	0,02174	0,73054	0,0065	0,71972	0,00335	0,59594	0,00396
127	0,6606	0,00763	0,66341	0,02275	0,73221	0,00704	0,72078	0,00352	0,5961	0,00566
128	0,66186	0,00885	0,66453	0,02075	0,73117	0,00819	0,71608	0,0052	0,59194	0,00407
129	0,66087	0,00846	0,66354	0,02133	0,72565	0,00757	0,71884	0,00302	0,58949	0,00309
130	0,66024	0,0093	0,66637	0,02081	0,7281	0,00628	0,71698	0,00332	0,59312	0,00514
131	0,65956	0,0127	0,66481	0,01973	0,72856	0,00564	0,72103	0,00536	0,59145	0,00582
132	0,65536	0,01622	0,66763	0,01978	0,72935	0,00655	0,71702	0,00345	0,59066	0,00562
133	0,65884	0,01103	0,66479	0,01886	0,72936	0,00745	0,7167	0,00556	0,59	0,00529
134	0,65304	0,01195	0,66633	0,02245	0,72821	0,00446	0,7176	0,00354	0,58226	0,0042
135	0,65549	0,0119	0,66495	0,02374	0,72826	0,00965	0,71965	0,00207	0,58375	0,0044
136	0,65848	0,00968	0,66251	0,0227	0,72678	0,00337	0,71399	0,00315	0,58714	0,00358
137	0,65409	0,0078	0,66598	0,02112	0,72478	0,00573	0,71287	0,00383	0,58175	0,00549
138	0,65598	0,00941	0,66234	0,02703	0,72755	0,00627	0,71315	0,00376	0,58359	0,00466
139	0,65609	0,00897	0,66527	0,02514	0,72259	0,00618	0,71623	0,00595	0,58257	0,004
140	0,65481	0,00978	0,66571	0,02286	0,72663	0,00827	0,71509	0,00332	0,58012	0,00356
141	0,65827	0,00988	0,6622	0,02408	0,72445	0,00811	0,71425	0,00561	0,57776	0,00543
142	0,65339	0,01075	0,66603	0,0253	0,72532	0,007	0,71443	0,00186	0,57611	0,00739
143	0,6526	0,01421	0,66032	0,02383	0,72436	0,00291	0,71424	0,00597	0,57859	0,003
144	0,65125	0,01158	0,66707	0,02365	0,72416	0,00809	0,71168	0,0095	0,57542	0,00582
145	0,65195	0,01245	0,66719	0,02106	0,72815	0,00357	0,71461	0,00351	0,57962	0,00422
146	0,65256	0,01064	0,67017	0,01929	0,72693	0,00301	0,71498	0,00689	0,57434	0,00555
147	0,65592	0,01184	0,67183	0,01997	0,72511	0,01008	0,70942	0,00289	0,57104	0,00875
148	0,6527	0,01093	0,67315	0,01954	0,72183	0,00557	0,71549	0,00506	0,57098	0,00619
149	0,64851	0,01024	0,6726	0,01799	0,72324	0,00748	0,71096	0,00416	0,56769	0,00605
150	0,65141	0,00985	0,67524	0,01863	0,72625	0,00316	0,71443	0,00557	0,57158	0,00509
151	0,65117	0,00832	0,67492	0,01584	0,72161	0,00579	0,71069	0,00512	0,5681	0,00681
152	0,65215	0,01295	0,67843	0,02312	0,725	0,00884	0,71181	0,00659	0,56799	0,0031
153	0,65362	0,01121	0,6732	0,02388	0,72155	0,00508	0,71174	0,00532	0,56341	0,00177
154	0,65119	0,01593	0,67428	0,02169	0,72485	0,00619	0,71445	0,0067	0,56121	0,0035
155	0,65184	0,01538	0,66976	0,02273	0,72279	0,00614	0,71172	0,00751	0,56777	0,00525
156	0,64983	0,01448	0,66831	0,01778	0,72138	0,00622	0,71059	0,00533	0,562	0,00779
157	0,65428	0,01367	0,66569	0,0182	0,7232	0,00641	0,71444	0,0027	0,5621	0,00487
158	0,65118	0,01514	0,66595	0,01666	0,72203	0,00707	0,71184	0,00374	0,56116	0,00276
159	0,65356	0,01065	0,66828	0,01944	0,72423	0,00412	0,70881	0,00671	0,5645	0,00291
160	0,65343	0,01293	0,67149	0,01514	0,7274	0,00596	0,7085	0,00452	0,56035	0,00368
161	0,65298	0,00824	0,6663	0,01797	0,72474	0,0111	0,71056	0,00729	0,55971	0,00676
162	0,65302	0,01112	0,67029	0,01573	0,72535	0,00336	0,70691	0,00504	0,55613	0,00619
163	0,65357	0,0107	0,66654	0,01833	0,72542	0,00602	0,71035	0,00731	0,55602	0,00219
164	0,65337	0,01201	0,66937	0,01629	0,72254	0,00471	0,71271	0,00339	0,55722	0,00136
165	0,6541	0,01359	0,66428	0,01301	0,72285	0,00339	0,70955	0,00784	0,55443	0,00978
166	0,65016	0,00833	0,66402	0,0192	0,71921	0,00586	0,71129	0,00672	0,5546	0,01143
167	0,65071	0,01056	0,66136	0,02079	0,72106	0,00403	0,71043	0,00664	0,55581	0,00682
168	0,65305	0,01024	0,66555	0,01755	0,7243	0,00776	0,71126	0,00706	0,55412	0,00726
169	0,65098	0,00974	0,66421	0,0184	0,72269	0,00804	0,70835	0,0057	0,55278	0,00527
170	0,65192	0,01346	0,66569	0,02148	0,7203	0,00197	0,70971	0,00545	0,54909	0,00734
171	0,6506	0,0101	0,66403	0,01693	0,72007	0,0034	0,71032	0,00317	0,5495	0,00617
172	0,64779	0,00731	0,66575	0,0169	0,72109	0,00304	0,71069	0,00658	0,54828	0,00488
173	0,65145	0,01228	0,66438	0,01689	0,71344	0,00416	0,71042	0,00684	0,54743	0,00976
174	0,65256	0,00836	0,66543	0,01944	0,72233	0,00561	0,70614	0,00577	0,54741	0,00463
175	0,65009	0,00943	0,66241	0,01758	0,71805	0,0041	0,70719	0,00385	0,54645	0,00692
176	0,64987	0,00958	0,66499	0,01737	0,71675	0,00414	0,70858	0,00469	0,54433	0,00449
177	0,64604	0,00977	0,66275	0,01811	0,71639	0,00529	0,7055	0,00764	0,54293	0,00483
178	0,6484	0,00914	0,66374	0,01902	0,71826	0,00365	0,70694	0,00449	0,54459	0,00542
179	0,64652	0,00836	0,66028	0,01837	0,71569	0,00769	0,70744	0,00415	0,54336	0,00705
180	0,64644	0,00852	0,66085	0,01797	0,71808	0,00466	0,7059	0,0087	0,53888	0,00979
181	0,65033	0,01064	0,66016	0,02032	0,71349	0,00584	0,70686	0,00593	0,54018	0,00723
182	0,64819	0,00791	0,66294	0,02021	0,71744	0,00758	0,70737	0,00302	0,53937	0,00819
183	0,64582	0,01203	0,66155	0,01665	0,71628	0,00873	0,71017	0,00342	0,53839	0,0093
184	0,64746	0,01138	0,65935	0,01915	0,71319	0,00559	0,70632	0,00764	0,53837	0,00602

185	0,64595	0,01491	0,65907	0,01862	0,71447	0,00985	0,70843	0,00683	0,53658	0,00908
186	0,64557	0,01031	0,65784	0,02387	0,71171	0,00453	0,70422	0,00368	0,53656	0,00822
187	0,64469	0,01273	0,66023	0,01908	0,71648	0,00461	0,70236	0,00464	0,53365	0,00549
188	0,64371	0,00814	0,66212	0,01919	0,71342	0,00543	0,70752	0,00649	0,53631	0,00966
189	0,64608	0,01465	0,65623	0,02069	0,71724	0,00262	0,70833	0,00664	0,5349	0,00795
190	0,64368	0,01335	0,65802	0,01799	0,7163	0,00759	0,70566	0,00755	0,53531	0,00406
191	0,64368	0,01268	0,65785	0,02552	0,71079	0,00551	0,70735	0,0019	0,53165	0,01042
192	0,64538	0,01327	0,65756	0,02086	0,71439	0,00586	0,70785	0,00698	0,52902	0,01103
193	0,64438	0,00864	0,65566	0,01869	0,7157	0,00567	0,70683	0,00709	0,5265	0,01007
194	0,64436	0,00995	0,65693	0,01781	0,71428	0,00977	0,70418	0,00432	0,52739	0,00952
195	0,64578	0,00967	0,65303	0,02088	0,7087	0,00635	0,70399	0,00714	0,52631	0,01062
196	0,64297	0,01166	0,65575	0,02029	0,71491	0,00757	0,70857	0,00791	0,52433	0,01164
197	0,64629	0,01174	0,65671	0,01825	0,71082	0,00659	0,70475	0,00338	0,52566	0,00959
198	0,645	0,01463	0,65208	0,02137	0,71103	0,00331	0,70662	0,00568	0,5278	0,00865
199	0,6392	0,01138	0,65386	0,02124	0,71084	0,00877	0,70819	0,00267	0,52462	0,00822
200	0,64551	0,01075	0,65382	0,01637	0,71191	0,00781	0,70703	0,0052	0,52451	0,00838
201	0,64495	0,01052	0,65831	0,01784	0,70801	0,0032	0,70554	0,00846	0,52231	0,00921
202	0,6436	0,01137	0,65648	0,01917	0,70717	0,00485	0,70287	0,0065	0,52316	0,00692
203	0,64603	0,01261	0,65553	0,02213	0,71039	0,00489	0,70757	0,00484	0,52321	0,00887
204	0,64027	0,00861	0,6561	0,01994	0,7095	0,00614	0,70545	0,00676	0,52147	0,0123
205	0,64366	0,01021	0,65657	0,01694	0,7102	0,00468	0,7029	0,00673	0,52139	0,0089
206	0,6416	0,00945	0,65905	0,01778	0,70913	0,00652	0,70533	0,00575	0,52073	0,01336
207	0,64028	0,00855	0,65951	0,0167	0,70758	0,00965	0,70451	0,00656	0,51992	0,00733
208	0,64619	0,01206	0,65384	0,01454	0,70753	0,00697	0,70557	0,00862	0,51785	0,01104
209	0,64342	0,00986	0,65597	0,01571	0,70983	0,00451	0,70507	0,00748	0,51979	0,01132
210	0,6409	0,01239	0,65399	0,01997	0,70668	0,00939	0,70429	0,00235	0,51618	0,01314
211	0,64155	0,01497	0,65364	0,02098	0,70921	0,00731	0,70312	0,00705	0,51821	0,00812
212	0,64095	0,00977	0,65029	0,02155	0,70508	0,00728	0,70792	0,00469	0,51813	0,0104
213	0,64198	0,00919	0,65343	0,01628	0,70713	0,00777	0,70379	0,00512	0,51422	0,00986
214	0,64181	0,00928	0,6542	0,02274	0,70863	0,00808	0,70992	0,00418	0,51678	0,01105
215	0,64087	0,01248	0,65317	0,01846	0,70735	0,01357	0,70196	0,00521	0,51506	0,00879
216	0,64091	0,01483	0,6509	0,02049	0,70694	0,00736	0,70494	0,00222	0,51284	0,0088
217	0,64048	0,01202	0,65592	0,01827	0,70683	0,00964	0,70579	0,00441	0,512	0,01
218	0,64401	0,01046	0,65171	0,01845	0,7063	0,00947	0,70301	0,0055	0,51196	0,00959
219	0,64239	0,01079	0,65331	0,01755	0,7035	0,0086	0,70361	0,00303	0,51234	0,00751
220	0,63976	0,00969	0,65097	0,02308	0,70763	0,00929	0,7049	0,00309	0,51284	0,00819
221	0,63738	0,00918	0,65389	0,01617	0,7078	0,00457	0,7026	0,00606	0,51008	0,01056
222	0,63395	0,01034	0,65361	0,01657	0,7061	0,00822	0,70602	0,00773	0,50781	0,00947
223	0,63824	0,00708	0,65066	0,02237	0,70328	0,01123	0,70358	0,0057	0,50964	0,00662
224	0,63839	0,0116	0,6517	0,01917	0,70317	0,01168	0,70495	0,00812	0,50676	0,00513
225	0,63921	0,01296	0,65268	0,01813	0,70386	0,0052	0,70318	0,00436	0,51091	0,00741
226	0,63764	0,01118	0,65028	0,01958	0,70127	0,00935	0,70399	0,00534	0,50781	0,00778
227	0,64025	0,01396	0,64889	0,0194	0,70397	0,00748	0,70278	0,00515	0,50986	0,00748
228	0,64172	0,01086	0,65032	0,01909	0,70333	0,00693	0,70178	0,00948	0,50975	0,00485
229	0,64178	0,01032	0,64761	0,02175	0,70418	0,00432	0,69743	0,00284	0,50417	0,00496
230	0,63847	0,01149	0,65471	0,02279	0,69983	0,00636	0,70083	0,00474	0,50663	0,00912
231	0,64154	0,01235	0,65423	0,01995	0,70052	0,00833	0,70354	0,00683	0,50619	0,00652
232	0,64065	0,00761	0,65056	0,01936	0,70377	0,0053	0,70367	0,00636	0,50381	0,00374
233	0,63647	0,01253	0,65139	0,0184	0,70311	0,00361	0,70314	0,00548	0,49972	0,00519
234	0,63928	0,00932	0,65153	0,02177	0,69799	0,00813	0,70051	0,00284	0,50236	0,00668
235	0,63691	0,00982	0,6473	0,01678	0,69805	0,00447	0,70035	0,00442	0,50207	0,00447
236	0,6387	0,01171	0,64795	0,01891	0,70072	0,00778	0,69997	0,00311	0,50587	0,00904
237	0,6392	0,00974	0,65211	0,02148	0,70009	0,00548	0,70257	0,00338	0,50043	0,00846
238	0,63755	0,01161	0,64795	0,01831	0,70058	0,00761	0,70276	0,00327	0,49816	0,00754
239	0,63824	0,0101	0,64681	0,01771	0,70284	0,00522	0,70253	0,00435	0,49963	0,00675
240	0,6364	0,00923	0,64444	0,01643	0,69992	0,00614	0,70243	0,00355	0,49957	0,00944
241	0,63436	0,01017	0,64452	0,01728	0,70306	0,00301	0,70339	0,00187	0,49843	0,00686
242	0,63736	0,01024	0,64795	0,01871	0,70215	0,00873	0,70148	0,00628	0,49869	0,00996
243	0,63863	0,00901	0,64184	0,01708	0,69935	0,00368	0,70119	0,00673	0,49854	0,00605
244	0,63553	0,00843	0,64604	0,01922	0,69612	0,0051	0,69937	0,00543	0,49777	0,00956
245	0,63946	0,00807	0,64529	0,02018	0,69908	0,00483	0,70337	0,00409	0,49373	0,00929
246	0,63531	0,01141	0,64259	0,02062	0,69718	0,00697	0,69561	0,00374	0,49483	0,0087
247	0,63844	0,01042	0,64301	0,01777	0,69503	0,00399	0,7011	0,00357	0,49473	0,01228
248	0,63386	0,00875	0,64216	0,01902	0,70167	0,00456	0,69942	0,00525	0,49462	0,00957
249	0,63541	0,00906	0,64068	0,02126	0,69589	0,00916	0,70091	0,00709	0,49544	0,00996
250	0,63521	0,00992	0,6437	0,02111	0,69474	0,00843	0,69569	0,00757	0,49459	0,00986
251	0,63744	0,00724	0,64004	0,02198	0,69852	0,00199	0,70367	0,00422	0,49358	0,0076
252	0,63588	0,01011	0,64024	0,02082	0,69787	0,0057	0,69944	0,00583	0,49355	0,0126
253	0,63683	0,00739	0,64164	0,02181	0,70203	0,00838	0,70234	0,00672	0,49284	0,0106
254	0,63868	0,00886	0,6392	0,02406	0,69904	0,00831	0,70014	0,00731	0,48821	0,00811
255	0,63492	0,00794	0,63892	0,02126	0,69965	0,00644	0,70046	0,00804	0,4874	0,00922
256	0,63626	0,00589	0,64072	0,02058	0,69543	0,00729	0,69335	0,00561	0,49107	0,00926
257	0,63817	0,00944	0,63977	0,0207	0,69982	0,00808	0,69722	0,00262	0,48956	0,00823
258	0,63615	0,00578	0,64105	0,02433	0,69846	0,00523	0,69864	0,00526	0,49027	0,00982
259	0,64117	0,00652	0,64155	0,02325	0,70148	0,00521	0,6975	0,00575	0,48699	0,01168
260	0,63661	0,00797	0,64097	0,02568	0,70082	0,00942	0,69556	0,00412	0,48467	0,0098
261	0,63465	0,00964	0,63838	0,02349	0,69921	0,00844	0,69976	0,00462	0,48983	0,01051
262	0,63583	0,00916	0,64029	0,01783	0,69947	0,00641	0,69979	0,00417	0,48917	0,00952
263	0,63478	0,00903	0,64044	0,02156	0,70588	0,00712	0,69712	0,00228	0,4853	0,00948
264	0,63769	0,01097	0,64085	0,02034	0,70106	0,00934	0,69562	0,00361	0,48628	0,01279
265	0,63526	0,00971	0,63938	0,02148	0,69592	0,00465	0,69513	0,00157	0,48381	0,00984

266	0,63367	0,00758	0,63939	0,02173	0,7009	0,00945	0,69947	0,00261	0,48389	0,00792
267	0,63371	0,00981	0,64161	0,02188	0,7001	0,00907	0,69922	0,00766	0,48174	0,00983
268	0,63451	0,00898	0,64355	0,02061	0,69846	0,00898	0,69486	0,00332	0,4839	0,01106
269	0,63731	0,00717	0,64085	0,02164	0,69914	0,01	0,69912	0,0053	0,47752	0,00875
270	0,63542	0,00521	0,64133	0,01716	0,6996	0,00963	0,70181	0,0077	0,48028	0,00472
271	0,62991	0,00969	0,64341	0,02227	0,69968	0,00836	0,69694	0,00238	0,4821	0,00475
272	0,63692	0,01026	0,64194	0,01927	0,69703	0,00896	0,69656	0,00297	0,48223	0,00601
273	0,63308	0,00925	0,64487	0,01881	0,70093	0,00553	0,70025	0,00343	0,4779	0,00944
274	0,63565	0,01009	0,63961	0,01907	0,69944	0,00941	0,69678	0,00303	0,48307	0,00732
275	0,63374	0,00902	0,64331	0,02099	0,70281	0,00549	0,69975	0,00567	0,48086	0,00331
276	0,63622	0,00988	0,6388	0,01829	0,69939	0,00819	0,70007	0,00834	0,48027	0,00948
277	0,63359	0,01298	0,6401	0,02219	0,69856	0,00556	0,70089	0,00153	0,47805	0,01007
278	0,63651	0,011	0,63778	0,02191	0,69927	0,00693	0,70058	0,00362	0,48133	0,00615
279	0,63479	0,01012	0,64143	0,02086	0,69982	0,00301	0,6989	0,0052	0,47616	0,00544
280	0,6317	0,01025	0,64195	0,01965	0,69533	0,00799	0,69914	0,00421	0,47689	0,0095
281	0,63464	0,00946	0,64087	0,02284	0,69638	0,00584	0,69891	0,00712	0,47568	0,00943
282	0,63355	0,01248	0,64578	0,02091	0,69483	0,00767	0,69827	0,00636	0,47327	0,00608
283	0,63484	0,00956	0,64382	0,01877	0,69547	0,00669	0,69796	0,00792	0,47623	0,00184
284	0,63372	0,01291	0,64386	0,01779	0,69774	0,00823	0,70017	0,00726	0,47631	0,00928
285	0,63295	0,01071	0,64249	0,01941	0,69532	0,00587	0,69921	0,00467	0,47806	0,00784
286	0,63625	0,01334	0,64027	0,02009	0,69809	0,00409	0,69862	0,00568	0,47531	0,01055
287	0,63716	0,00877	0,64448	0,01723	0,69692	0,00559	0,70005	0,00539	0,47263	0,00733
288	0,63342	0,01039	0,64472	0,02273	0,69751	0,00608	0,69828	0,00649	0,47422	0,00611
289	0,63078	0,01288	0,64197	0,02083	0,69774	0,00515	0,69972	0,00371	0,47622	0,00975
290	0,63471	0,00987	0,6428	0,01809	0,69764	0,00669	0,70053	0,00622	0,47245	0,00655
291	0,6326	0,01406	0,64407	0,02058	0,698	4,76733E-4	0,69829	0,00665	0,47434	0,01151
292	0,63436	0,00917	0,64245	0,01986	0,69859	0,00544	0,70151	0,00554	0,47135	0,00713
293	0,63481	0,01279	0,64261	0,017	0,69651	0,00676	0,69705	0,00204	0,47306	0,00721
294	0,63397	0,00949	0,64381	0,01773	0,69387	0,00713	0,69603	0,00365	0,47039	0,0081
295	0,63482	0,01256	0,64476	0,01899	0,69973	0,00507	0,70102	0,00926	0,47175	0,01365
296	0,63714	0,01191	0,64368	0,02076	0,6993	0,00752	0,69801	0,0055	0,47244	0,00867
297	0,63521	0,01248	0,64215	0,02239	0,7009	0,01047	0,70092	0,00399	0,46986	0,01089
298	0,63571	0,00851	0,64235	0,02096	0,69928	0,00642	0,6973	0,0065	0,46745	0,00806
299	0,6328	0,01197	0,64046	0,02084	0,70003	0,00775	0,69654	0,00284	0,46613	0,00816
300	0,63695	0,00818	0,64283	0,01974	0,69806	0,00783	0,69842	0,00291	0,46816	0,00508
301	0,63311	0,01022	0,64267	0,02045	0,69787	0,004	0,69347	0,00425	0,46939	0,01086
302	0,63409	0,0086	0,64186	0,01931	0,69799	0,00886	0,70174	0,00409	0,46999	0,00897
303	0,63578	0,00952	0,63895	0,01935	0,69552	0,00561	0,69891	0,00346	0,46379	0,00688
304	0,63121	0,00945	0,64361	0,01772	0,69938	0,00955	0,69386	0,00499	0,46947	0,00653
305	0,63248	0,01099	0,64314	0,02606	0,69869	0,00516	0,69806	0,00562	0,46513	0,0056
306	0,63365	0,0104	0,63859	0,02078	0,69893	0,00641	0,69532	0,00477	0,46617	0,00994
307	0,63334	0,0074	0,63957	0,02018	0,69644	0,00409	0,69469	0,0031	0,46628	0,00644
308	0,63511	0,011	0,6402	0,01965	0,69896	0,0046	0,69695	0,00561	0,46688	0,0081
309	0,63195	0,0102	0,64191	0,02342	0,7001	0,00668	0,69583	0,00679	0,46686	0,00466
310	0,6358	0,0107	0,64287	0,02028	0,69796	0,00711	0,69559	0,00625	0,46813	0,00852
311	0,62959	0,0085	0,63859	0,02056	0,69799	0,0053	0,69919	0,00533	0,46628	0,01009
312	0,63291	0,0058	0,63758	0,01675	0,69676	0,00662	0,69887	0,00235	0,46537	0,00957
313	0,6331	0,00916	0,63919	0,01903	0,70042	0,00973	0,70063	0,00457	0,46418	0,00766
314	0,63004	0,0113	0,64059	0,01922	0,69871	0,00502	0,69957	0,00965	0,46213	0,00735
315	0,62971	0,00889	0,64235	0,01803	0,70037	0,00306	0,70261	0,00254	0,46522	0,00878
316	0,6346	0,00661	0,64357	0,02029	0,69418	0,00587	0,69646	0,00518	0,46407	0,01221
317	0,63318	0,01015	0,63654	0,02136	0,7001	0,00814	0,69825	0,0037	0,46189	0,00747
318	0,63301	0,00883	0,64309	0,02141	0,7004	0,0063	0,70021	0,00359	0,45913	0,0118
319	0,63259	0,01032	0,63979	0,02072	0,69524	0,00897	0,69887	0,00424	0,46165	0,007
320	0,63283	0,00741	0,64089	0,0192	0,69956	0,00685	0,69925	0,00464	0,46	0,01151
321	0,63245	0,00934	0,64141	0,02136	0,69845	0,00482	0,69954	0,00391	0,45968	0,00798
322	0,63067	0,00951	0,64004	0,01579	0,69906	0,00688	0,69504	0,00554	0,46262	0,01134
323	0,63147	0,00911	0,64096	0,02029	0,70179	0,00497	0,69703	0,00128	0,45929	0,01061
324	0,62953	0,01001	0,64074	0,02213	0,69416	0,00809	0,70045	0,00378	0,45975	0,01163
325	0,63156	0,01325	0,64012	0,01852	0,69622	0,00511	0,6979	0,00324	0,46017	0,00805
326	0,63157	0,00876	0,63819	0,02125	0,69741	0,00842	0,69663	0,00629	0,46236	0,00996
327	0,63185	0,012	0,63731	0,02002	0,7012	0,00904	0,69898	0,0051	0,46176	0,00787
328	0,6326	0,00613	0,63661	0,02239	0,69681	0,00615	0,69836	0,00532	0,45667	0,01127
329	0,63242	0,0113	0,63892	0,01772	0,69927	0,0062	0,69832	0,0052	0,45851	0,01094
330	0,62981	0,01375	0,63808	0,02143	0,69832	0,00726	0,69453	0,00924	0,45677	0,00942
331	0,635	0,01478	0,63893	0,02306	0,69747	0,00483	0,69518	0,0092	0,45869	0,00872
332	0,63403	0,01003	0,64091	0,01826	0,69896	0,00891	0,69659	0,00502	0,45504	0,0107
333	0,63114	0,00794	0,63811	0,02246	0,6936	0,00774	0,69715	0,00576	0,45907	0,01171
334	0,63185	0,00899	0,63954	0,02176	0,69779	9,42689E-4	0,69406	0,00697	0,45582	0,0115
335	0,63368	0,00744	0,64082	0,02096	0,69586	0,00448	0,6982	0,00553	0,45479	0,00918
336	0,6283	0,00853	0,63832	0,02255	0,69789	0,00874	0,69616	0,00579	0,45835	0,00744
337	0,63251	0,00955	0,63901	0,0215	0,69577	0,00483	0,70138	0,00668	0,4566	0,00729
338	0,63099	0,0092	0,63761	0,02027	0,69616	0,01113	0,7006	0,00401	0,45675	0,00696
339	0,63574	0,01054	0,6379	0,02233	0,69728	0,01027	0,69787	0,00643	0,4558	0,00938
340	0,63343	0,01337	0,63703	0,02032	0,69683	0,00766	0,69729	0,0032	0,4527	0,00615
341	0,63376	0,01249	0,63772	0,02242	0,69479	0,00484	0,69646	0,00597	0,45348	0,00693
342	0,63397	0,01077	0,63789	0,02318	0,69791	0,01078	0,69723	0,00321	0,45284	0,00624
343	0,62937	0,01026	0,63962	0,02242	0,69956	0,00154	0,69604	0,00544	0,45108	0,00934
344	0,63418	0,00887	0,63894	0,01866	0,69691	0,0083	0,69902	0,00314	0,45274	0,00803

345	0,63178	0,01053	0,63492	0,02166	0,69674	0,01007	0,69621	0,00547	0,44798	0,00789
346	0,632	0,0138	0,63978	0,02095	0,69328	0,00753	0,69815	0,00491	0,45215	0,0082
347	0,63392	0,00997	0,63715	0,02126	0,69469	0,00889	0,69606	0,00945	0,44999	0,01151
348	0,63186	0,00912	0,63678	0,02472	0,69611	0,00748	0,69744	0,00777	0,45324	0,00734
349	0,63024	0,00817	0,63843	0,02184	0,69309	0,00543	0,69847	0,00347	0,45062	0,00691
350	0,63346	0,01303	0,63547	0,02121	0,69793	0,00439	0,69874	0,00502	0,45266	0,00817
351	0,63089	0,01164	0,63863	0,01895	0,69529	0,0062	0,69873	0,00606	0,45003	0,00709
352	0,63285	0,01012	0,64072	0,02064	0,69564	0,00588	0,69632	0,00317	0,4489	0,00833
353	0,63145	0,00992	0,63835	0,02244	0,69606	0,00885	0,69563	0,00309	0,4511	0,01053
354	0,63009	0,01075	0,6398	0,01973	0,6972	0,00715	0,69622	0,00414	0,44805	0,00976
355	0,63014	0,01025	0,63931	0,0225	0,69689	0,00874	0,69482	0,00716	0,44648	0,0071
356	0,63531	0,00867	0,64117	0,02036	0,69705	0,00634	0,69938	0,00757	0,44714	0,00951
357	0,62857	0,00965	0,63988	0,01934	0,69427	0,00497	0,69501	0,00653	0,44817	0,0086
358	0,63046	0,00532	0,6388	0,02651	0,69553	0,00442	0,69872	0,00601	0,44764	0,00866
359	0,63001	0,01031	0,63879	0,02239	0,69708	0,00295	0,6982	0,0052	0,44749	0,01046
360	0,63089	0,01548	0,63502	0,01818	0,69659	0,00824	0,69615	0,00311	0,44266	0,00937
361	0,6319	0,00649	0,63746	0,01861	0,69698	0,00598	0,70139	0,00255	0,4445	0,00641
362	0,63448	0,01128	0,64099	0,02087	0,69574	0,00814	0,69954	0,00401	0,44851	0,00923
363	0,63475	0,00828	0,63886	0,02251	0,6936	0,00269	0,70027	0,0086	0,44545	0,00992
364	0,62987	0,01074	0,64132	0,02431	0,69333	0,01	0,69495	0,00715	0,44406	0,00856
365	0,62817	0,01107	0,63825	0,01911	0,69658	0,00787	0,6993	0,00579	0,44283	0,00771
366	0,63213	0,00976	0,64318	0,02225	0,70037	0,0077	0,69893	0,00476	0,44569	0,00902
367	0,6291	0,01107	0,63974	0,02084	0,69532	0,00669	0,69908	0,00709	0,4463	0,0099
368	0,6319	0,008	0,64083	0,02098	0,69661	0,00604	0,69922	0,00123	0,443	0,00956
369	0,62902	0,00995	0,64029	0,02289	0,70094	0,00712	0,70269	0,00585	0,44587	0,00958
370	0,63098	0,01143	0,64123	0,01941	0,69583	0,00379	0,70086	0,00558	0,44477	0,00664
371	0,63061	0,0078	0,63937	0,02148	0,69809	0,00724	0,69808	0,00864	0,44689	0,0099
372	0,62997	0,00824	0,64063	0,01948	0,6957	0,00474	0,69719	0,00561	0,44277	0,00875
373	0,62902	0,0091	0,63691	0,02104	0,69465	0,00681	0,69751	0,0063	0,44375	0,00836
374	0,6328	0,01166	0,6395	0,02087	0,69548	0,00903	0,70003	0,00548	0,4474	0,0076
375	0,63045	0,01239	0,64513	0,02014	0,69663	0,00751	0,70018	0,0064	0,44586	0,00933
376	0,62928	0,01117	0,63752	0,01873	0,69548	0,00555	0,69692	0,00451	0,44615	0,00989
377	0,63113	0,00871	0,6377	0,02042	0,69463	0,00727	0,69741	0,00813	0,44674	0,00809
378	0,63025	0,00809	0,64078	0,02332	0,69643	0,00602	0,70013	0,00436	0,44167	0,00831
379	0,63175	0,00932	0,63804	0,02001	0,6966	0,00755	0,70225	0,00491	0,44296	0,01195
380	0,63082	0,01224	0,63978	0,01854	0,69789	0,00845	0,69676	0,00664	0,44297	0,00633
381	0,63282	0,00823	0,64009	0,0201	0,69393	0,00756	0,70008	0,00347	0,44396	0,01079
382	0,63464	0,00825	0,63799	0,01697	0,69459	0,00652	0,69734	0,00465	0,44352	0,012
383	0,63169	0,01097	0,64412	0,01529	0,69516	0,00573	0,69747	0,00498	0,44301	0,01071
384	0,62912	0,01283	0,64046	0,02094	0,69539	0,00332	0,69813	0,00715	0,44215	0,00928
385	0,63265	0,01243	0,63655	0,01765	0,69872	0,00704	0,70126	0,00646	0,44124	0,01009
386	0,6339	0,01175	0,64135	0,01941	0,69868	0,00532	0,69512	0,00531	0,44087	0,00985
387	0,63173	0,01273	0,63956	0,02082	0,69624	0,00574	0,69912	0,00542	0,43805	0,00963
388	0,62921	0,0095	0,6373	0,0192	0,69561	0,00838	0,69786	0,00504	0,44119	0,00849
389	0,6282	0,01174	0,63985	0,02029	0,69569	0,00926	0,70031	0,00678	0,44231	0,01168
390	0,63198	0,01143	0,63924	0,01908	0,6963	0,00914	0,69939	0,00603	0,43759	0,01102
391	0,63033	0,01105	0,63978	0,01818	0,69472	0,00661	0,6951	0,00538	0,43952	0,0134
392	0,63068	0,01048	0,63953	0,0197	0,70021	0,00745	0,69584	0,00567	0,43925	0,01044
393	0,63341	0,01373	0,6393	0,01957	0,69767	0,00494	0,69828	0,00647	0,4435	0,00679
394	0,63236	0,00394	0,63833	0,01978	0,69543	0,00857	0,69925	0,00716	0,43971	0,01041
395	0,6299	0,01027	0,64068	0,017	0,69673	0,00442	0,70227	0,00604	0,44361	0,00896
396	0,62769	0,01043	0,64175	0,02143	0,69286	0,00821	0,69741	0,00855	0,43887	0,0102
397	0,63352	0,01392	0,63588	0,02107	0,69986	0,00832	0,69778	0,0054	0,44063	0,00891
398	0,63435	0,01209	0,63839	0,01698	0,69702	0,00204	0,69663	0,00709	0,43566	0,00911
399	0,63022	0,01169	0,63889	0,01647	0,70414	0,00453	0,69745	0,00354	0,43523	0,01056
400	0,63007	0,00884	0,63906	0,02038	0,69442	0,00809	0,6971	1,48406E-4	0,44088	0,01122
401	0,62965	0,0103	0,63822	0,01728	0,69659	0,00168	0,69511	0,00477	0,43559	0,00933
402	0,63279	0,01037	0,64165	0,01923	0,69415	0,00726	0,69715	0,00334	0,4364	0,00761
403	0,62985	0,00881	0,63938	0,02079	0,69899	0,00368	0,69735	0,00678	0,43595	0,00711
404	0,6322	0,00684	0,63912	0,02065	0,69575	0,00928	0,69968	0,00367	0,43599	0,01072
405	0,63245	0,00926	0,6385	0,02025	0,69895	0,00685	0,70043	0,00503	0,4373	0,00928
406	0,63173	0,00764	0,64156	0,0219	0,69812	0,0084	0,69984	0,00608	0,43482	0,00614
407	0,63317	0,0126	0,63942	0,01886	0,69646	0,00252	0,69716	0,0042	0,43393	0,00809
408	0,63034	0,01187	0,64115	0,02144	0,69595	0,00681	0,69714	0,0071	0,43876	0,00725
409	0,62876	0,01145	0,6423	0,01629	0,69496	0,00546	0,702	0,00459	0,43465	0,0074
410	0,6335	0,00765	0,63916	0,02229	0,69518	0,00804	0,70023	0,00441	0,43557	0,00905
411	0,6334	0,01336	0,63763	0,01978	0,69401	0,00649	0,69652	0,00578	0,43322	0,01399
412	0,63105	0,01427	0,64138	0,0198	0,69674	0,00574	0,69819	7,82965E-4	0,43447	0,01185
413	0,62904	0,01116	0,63888	0,02226	0,69109	0,00737	0,69923	0,00386	0,4345	0,0061
414	0,63608	0,00714	0,63896	0,02079	0,69996	0,00941	0,69802	0,00552	0,43681	0,00759
415	0,63133	0,01028	0,6415	0,01993	0,69951	0,00767	0,69806	0,00438	0,43733	0,00728
416	0,6297	0,01355	0,64102	0,01917	0,69844	0,00467	0,69753	0,00339	0,43389	0,00682
417	0,62767	0,00951	0,63491	0,02342	0,69409	0,00954	0,70045	0,00556	0,43538	0,00807
418	0,63019	0,01002	0,63637	0,02054	0,6998	0,00832	0,69845	0,00376	0,4322	0,00899
419	0,62974	0,01124	0,63906	0,02008	0,69665	0,00662	0,69697	0,00574	0,43356	0,00531
420	0,63153	0,0104	0,64084	0,02305	0,6991	0,00504	0,69736	0,00173	0,4324	0,00979
421	0,62904	0,00947	0,63959	0,01707	0,69791	0,00552	0,69626	0,00665	0,43289	0,00839
422	0,63234	0,01114	0,6386	0,0177	0,69404	0,00489	0,69805	0,0078	0,43323	0,0111
423	0,62752	0,0036	0,63745	0,02371	0,69456	0,00747	0,70155	0,00854	0,43237	0,00908

424	0,63119	0,00843	0,63746	0,02082	0,69835	0,00396	0,70013	0,00471	0,4317	0,00832
425	0,63069	0,00849	0,63752	0,0173	0,69626	0,00399	0,69605	0,00522	0,43039	0,0081
426	0,62909	0,01133	0,63552	0,02064	0,69744	0,00795	0,69781	0,00701	0,43019	0,01315
427	0,62928	0,00866	0,64112	0,0204	0,6983	0,00745	0,69939	0,00536	0,43064	0,01315
428	0,6337	0,01	0,63941	0,02309	0,69712	0,00524	0,69659	0,00734	0,42924	0,01223
429	0,62725	0,00879	0,6381	0,02384	0,69573	0,01144	0,69918	0,00425	0,43096	0,00923
430	0,63257	0,00907	0,64107	0,01956	0,69379	0,00249	0,69883	0,00577	0,43041	0,0076
431	0,62963	0,00734	0,64267	0,02352	0,69825	0,00466	0,69732	0,00689	0,43126	0,00908
432	0,62739	0,01144	0,63725	0,02287	0,69538	0,00288	0,69765	0,00319	0,42893	0,01026
433	0,6331	0,00848	0,63654	0,02282	0,69569	0,00507	0,70106	0,00343	0,42938	0,01087
434	0,63204	0,01243	0,63845	0,02064	0,69306	0,00367	0,70021	0,00818	0,42956	0,00918
435	0,63327	0,00829	0,64052	0,02098	0,69673	0,0069	0,70167	0,0037	0,42696	0,00787
436	0,63184	0,01249	0,6384	0,02013	0,69354	0,00664	0,69826	0,00544	0,43136	0,01195
437	0,6323	0,01072	0,63969	0,01793	0,69784	0,00378	0,69446	0,0058	0,43093	0,01065
438	0,63181	0,00903	0,6412	0,02197	0,693	0,00603	0,69462	0,00498	0,4302	0,00925
439	0,63388	0,01167	0,63747	0,02284	0,70084	0,00639	0,6984	0,00538	0,4272	0,01104
440	0,62991	0,00876	0,63743	0,01951	0,69807	0,00865	0,69605	0,00319	0,42812	0,01186
441	0,63065	0,0109	0,64152	0,02073	0,69856	0,00678	0,70193	0,00482	0,42842	0,01036
442	0,63045	0,01169	0,64159	0,0253	0,69596	0,00837	0,69588	0,00403	0,42896	0,00988
443	0,62886	0,01048	0,63728	0,02111	0,69618	0,00681	0,69823	0,00559	0,43229	0,01031
444	0,63171	0,01142	0,63915	0,01727	0,69617	0,00762	0,70195	0,00499	0,42734	0,01089
445	0,63281	0,01042	0,63954	0,02023	0,69498	0,00644	0,69813	0,0037	0,42822	0,0102
446	0,63147	0,01026	0,63804	0,02054	0,69891	0,00713	0,69741	0,00485	0,42922	0,00906
447	0,63094	0,01125	0,6357	0,02193	0,69491	0,00511	0,69547	0,00582	0,42841	0,01272
448	0,63481	0,01323	0,63828	0,02226	0,69496	0,00474	0,69991	0,00151	0,42835	0,01123
449	0,63119	0,01193	0,63866	0,02346	0,6968	0,00462	0,69757	0,00228	0,42634	0,00831
450	0,63051	0,01223	0,63692	0,01727	0,69547	0,00474	0,69741	0,00279	0,42647	0,0118
451	0,62907	0,01195	0,63354	0,02163	0,69559	0,00482	0,69702	0,00411	0,42504	0,01061
452	0,63454	0,00971	0,63869	0,01928	0,69359	0,0056	0,70147	0,00867	0,42564	0,01132
453	0,6305	0,01303	0,63786	0,01917	0,69444	0,00563	0,69846	0,00398	0,42423	0,00775
454	0,63401	0,01458	0,63853	0,02052	0,69289	0,01035	0,70128	0,00766	0,42578	0,00957
455	0,62949	0,01159	0,63564	0,02126	0,69278	0,00516	0,70169	0,00587	0,42947	0,0114
456	0,63176	0,00901	0,63702	0,01782	0,69749	0,00736	0,69756	0,00827	0,42697	0,01384
457	0,63205	0,01193	0,63978	0,01863	0,69745	0,00199	0,69856	9,41837E-4	0,42616	0,01105
458	0,63208	0,01255	0,63836	0,01966	0,69553	0,0069	0,69817	0,00475	0,42621	0,00898
459	0,63345	0,01075	0,63541	0,01519	0,6909	0,00268	0,69985	0,00708	0,42368	0,01206
460	0,6298	0,01267	0,63818	0,02248	0,69755	0,00905	0,69958	0,00414	0,42265	0,01115
461	0,6314	0,01467	0,6368	0,02092	0,69677	0,00404	0,69951	0,00454	0,42237	0,01217
462	0,62953	0,01067	0,63778	0,01784	0,69559	0,0058	0,69759	0,00253	0,42168	0,01371
463	0,62825	0,00918	0,63603	0,02142	0,69458	0,00538	0,69835	0,00382	0,42603	0,00843
464	0,63219	0,01063	0,64113	0,01866	0,69288	0,00526	0,70248	0,00723	0,42336	0,01307
465	0,63211	0,01241	0,63841	0,01719	0,69548	0,00901	0,6971	0,0057	0,42114	0,00906
466	0,63044	0,01272	0,63813	0,0187	0,69557	0,00479	0,70185	0,00509	0,42527	0,01191
467	0,63345	0,01274	0,63951	0,02233	0,69762	0,00449	0,69894	0,00736	0,42408	0,01008
468	0,63065	0,01083	0,63688	0,02139	0,69274	0,00244	0,69884	0,0058	0,42348	0,00982
469	0,63212	0,01133	0,63865	0,02322	0,69748	0,00337	0,69831	0,00368	0,42291	0,01039
470	0,63452	0,00866	0,63609	0,01948	0,69611	0,00503	0,69474	0,00654	0,42073	0,00941
471	0,62979	0,01299	0,6371	0,02131	0,69792	0,00661	0,69999	0,00589	0,42067	0,00763
472	0,63124	0,00887	0,63835	0,01973	0,69647	0,00515	0,70017	0,00506	0,42222	0,01107
473	0,62889	0,01322	0,63733	0,0189	0,69085	0,00507	0,69851	0,00645	0,42139	0,00968
474	0,63136	0,01107	0,63895	0,0183	0,69903	0,00426	0,70108	0,01	0,42084	0,01114
475	0,63179	0,01257	0,64041	0,01923	0,69462	0,00994	0,69798	0,00602	0,42162	0,01007
476	0,63477	0,01427	0,63746	0,01889	0,69442	0,00505	0,69702	0,00635	0,42003	0,01311
477	0,6329	0,0113	0,63427	0,02178	0,69485	0,00897	0,69676	0,00459	0,42061	0,00995
478	0,63165	0,01212	0,63896	0,02388	0,69651	0,00994	0,70119	0,00426	0,42104	0,01246
479	0,63068	0,01242	0,63439	0,0171	0,69572	0,00681	0,69758	0,00896	0,42296	0,00928
480	0,63121	0,01194	0,63684	0,01998	0,6943	0,008	0,6968	0,00524	0,42264	0,00862
481	0,63053	0,01061	0,64055	0,01913	0,6965	0,00584	0,69947	0,00203	0,41609	0,01136
482	0,6321	0,01352	0,63756	0,02019	0,69307	0,0064	0,7002	0,0053	0,42078	0,00852
483	0,62855	0,00958	0,6371	0,02019	0,69728	0,0037	0,69442	0,00262	0,41947	0,01153
484	0,63071	0,00999	0,63538	0,02069	0,70034	0,00234	0,69964	0,00257	0,41916	0,00962
485	0,63245	0,01106	0,63698	0,02184	0,69421	0,00688	0,70125	0,00768	0,4209	0,0062
486	0,6327	0,01004	0,63835	0,02243	0,69661	0,00738	0,69807	0,00814	0,41886	0,00968
487	0,63344	0,01076	0,64194	0,02026	0,69415	0,00486	0,69752	0,00446	0,41755	0,00987
488	0,63048	0,01071	0,63672	0,0203	0,69522	0,00525	0,69856	0,00697	0,41865	0,00778
489	0,63375	0,0104	0,64118	0,02046	0,69798	0,00456	0,69614	0,00638	0,41743	0,01151
490	0,63147	0,01037	0,63979	0,01828	0,69821	0,0069	0,69919	0,00596	0,4168	0,00999
491	0,63031	0,00876	0,63893	0,02089	0,6975	0,00741	0,69925	0,00737	0,41777	0,01254
492	0,63212	0,01357	0,63851	0,02171	0,69168	0,00352	0,69865	0,00706	0,41948	0,00985
493	0,63113	0,00979	0,63933	0,0228	0,69917	0,00688	0,69506	0,00455	0,41818	0,01005
494	0,63282	0,01018	0,63643	0,01906	0,69423	0,00881	0,6982	0,00664	0,41729	0,01168
495	0,62658	0,01179	0,63745	0,02214	0,69806	0,00762	0,70277	0,00527	0,41986	0,01019
496	0,63428	0,00753	0,64192	0,01958	0,69667	0,00574	0,69349	0,00368	0,41566	0,00697
497	0,63158	0,00908	0,63725	0,01991	0,6938	0,00707	0,6959	0,00429	0,41626	0,00826
498	0,63212	0,01234	0,63885	0,01811	0,69576	0,00459	0,69521	0,00747	0,41625	0,01065
499	0,63143	0,01228	0,63813	0,02467	0,70031	0,00691	0,69792	0,00375	0,41666	0,00916
500	0,63346	0,01236	0,63614	0,02083	0,69805	0,00721	0,69994	0,00433	0,41888	0,00974
501	0,63552	0,01013	0,6368	0,01932	0,69352	0,00673	0,6997	0,00612	0,4165	0,01019
502	0,63038	0,00855	0,63866	0,01994	0,69666	0,00381	0,69719	0,00539	0,41849	0,01343
503	0,63227	0,0107	0,63708	0,02371	0,69758	0,00704	0,69745	0,00418	0,42225	0,00711

504	0,63214	0,01061	0,63619	0,01866	0,69693	0,00748	0,69605	0,00658	0,41622	0,01169
505	0,62882	0,01245	0,63789	0,02176	0,69316	0,00596	0,69943	0,00617	0,41762	0,00999
506	0,63262	0,01249	0,63931	0,02222	0,69553	0,00354	0,69832	0,0061	0,41429	0,01251
507	0,63663	0,01198	0,63731	0,02142	0,69233	0,00837	0,69699	0,00709	0,41527	0,0101

Copies of ^{1}H and ^{13}C NMR spectra of new compounds

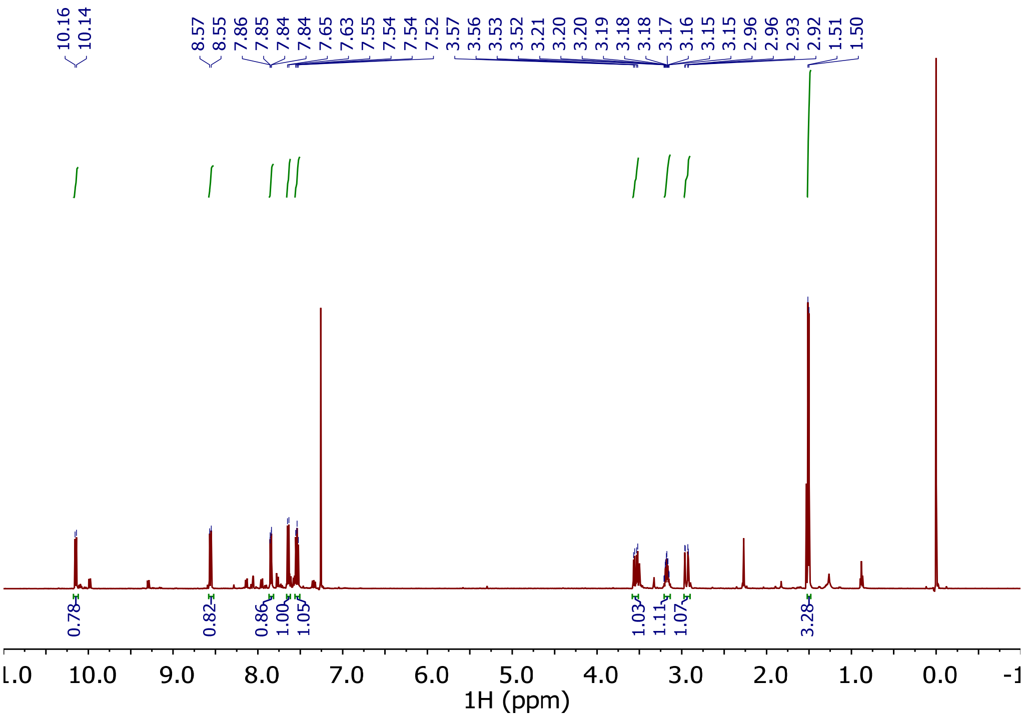

Supplementary Figure 31. NMR spectrum. ^{1}H NMR spectrum of impure thioketone **4** (500 MHz, $CDCl_3$, 298K).

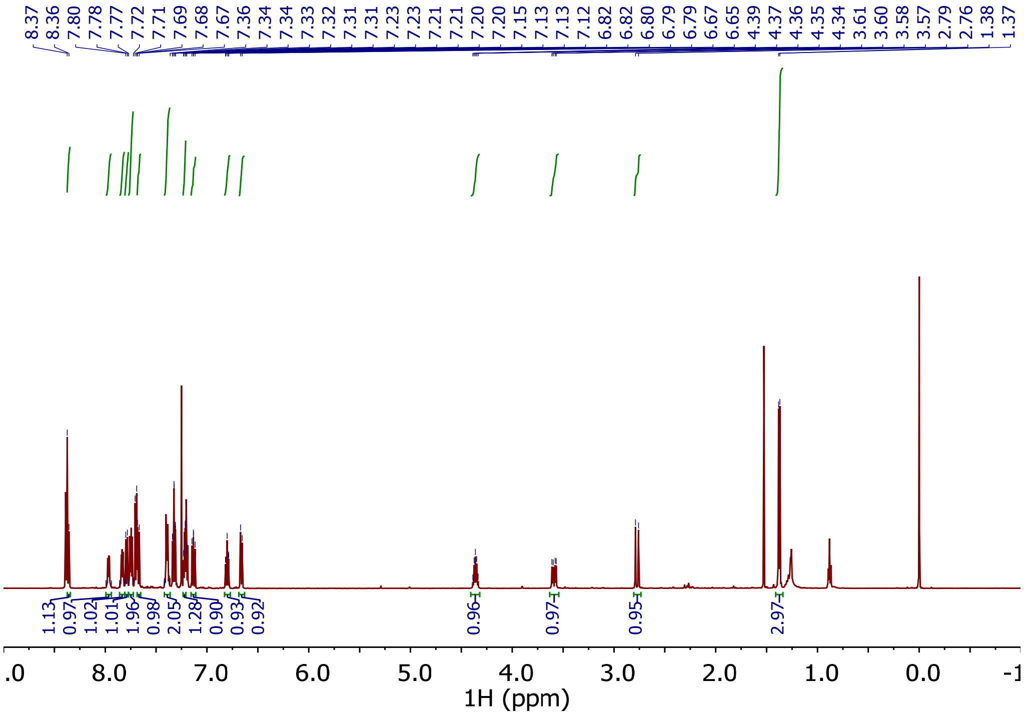

Supplementary Figure 32. NMR spectrum. ^{1}H NMR spectrum of impure bromomotor **5** (500 MHz, $CDCl_3$, 298K).

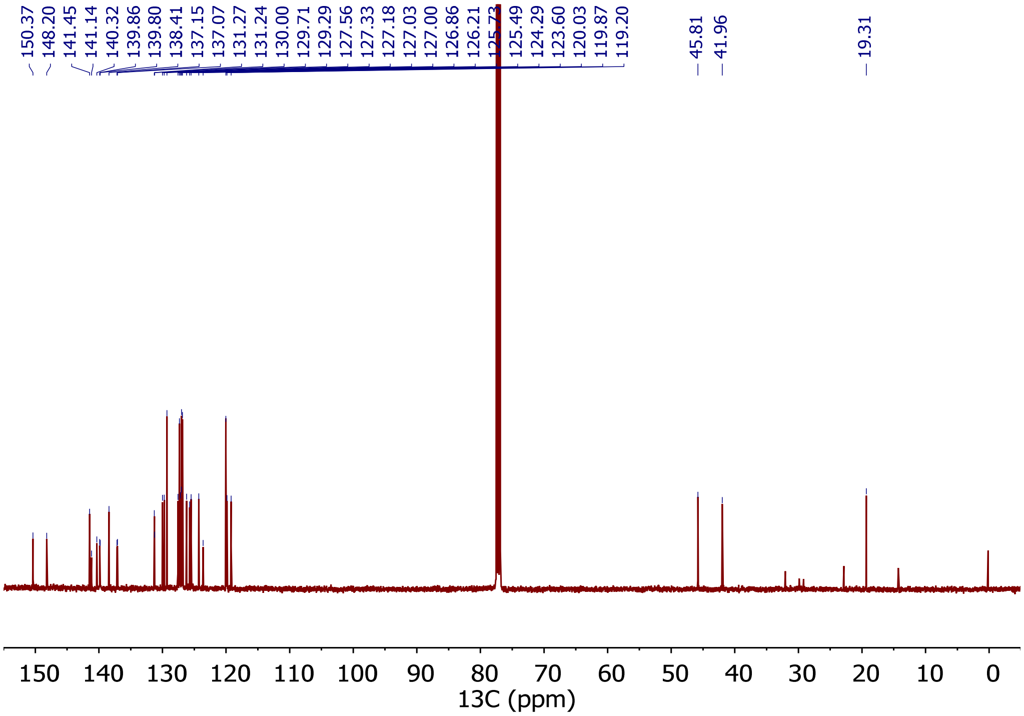

Supplementary Figure 33. NMR spectrum. ^{13}C NMR spectrum of impure bromomotor **5** (126 MHz, $CDCl_3$, 298K).

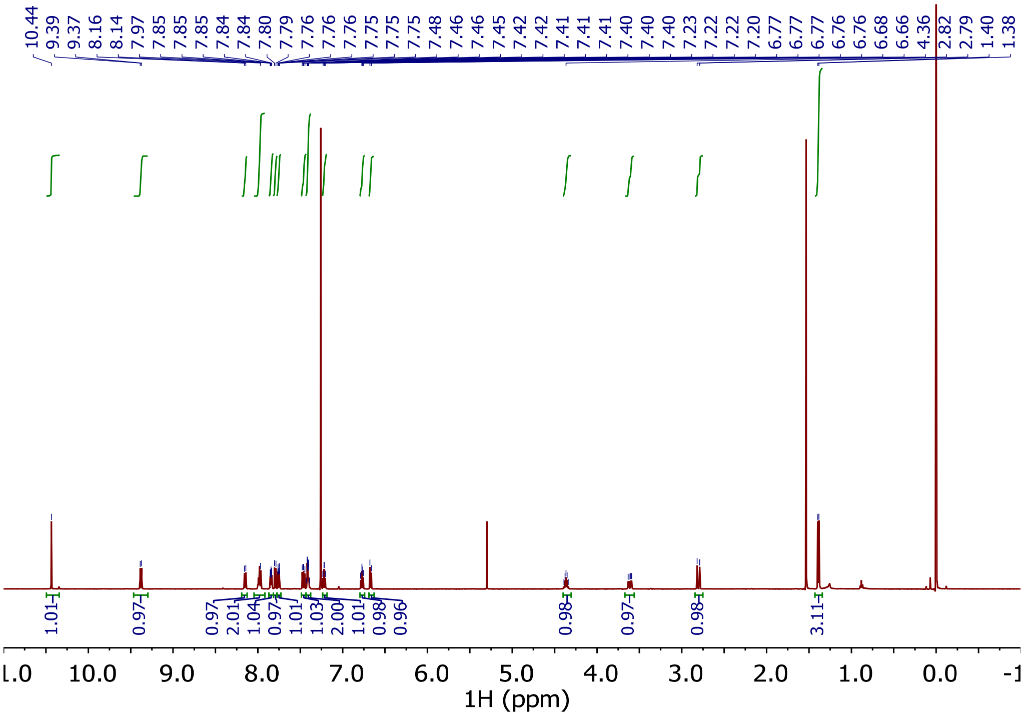

Supplementary Figure 34. NMR spectrum. ^{1}H NMR spectrum of aldehyde motor **6** (500 MHz, $CDCl_3$, 298K).

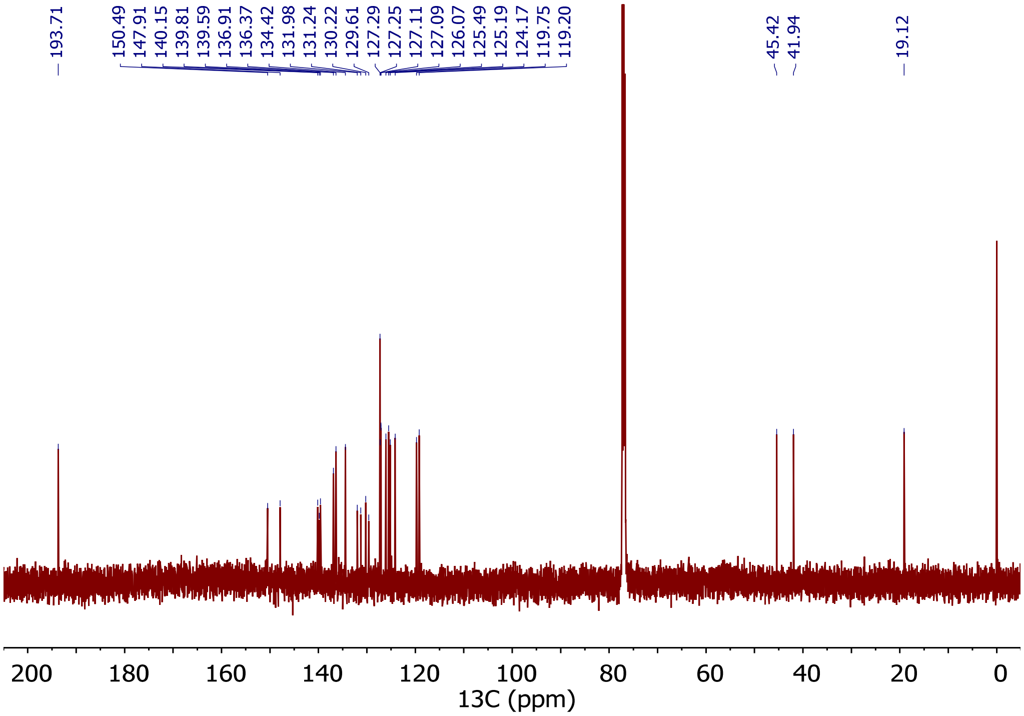

Supplementary Figure 35. NMR spectrum. ^{13}C NMR spectrum of aldehyde motor **6** (126 MHz, $CDCl_3$, 298K).

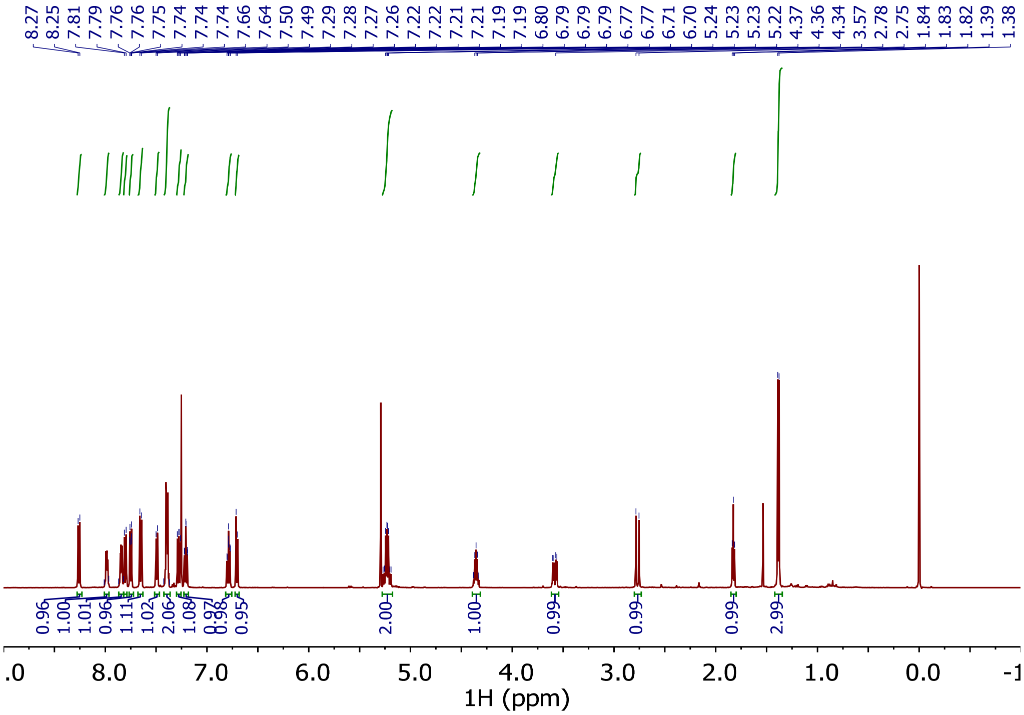

Supplementary Figure 36. NMR spectrum. ^{1}H NMR spectrum of alcohol motor **7** (500 MHz, $CDCl_3$, 298K).

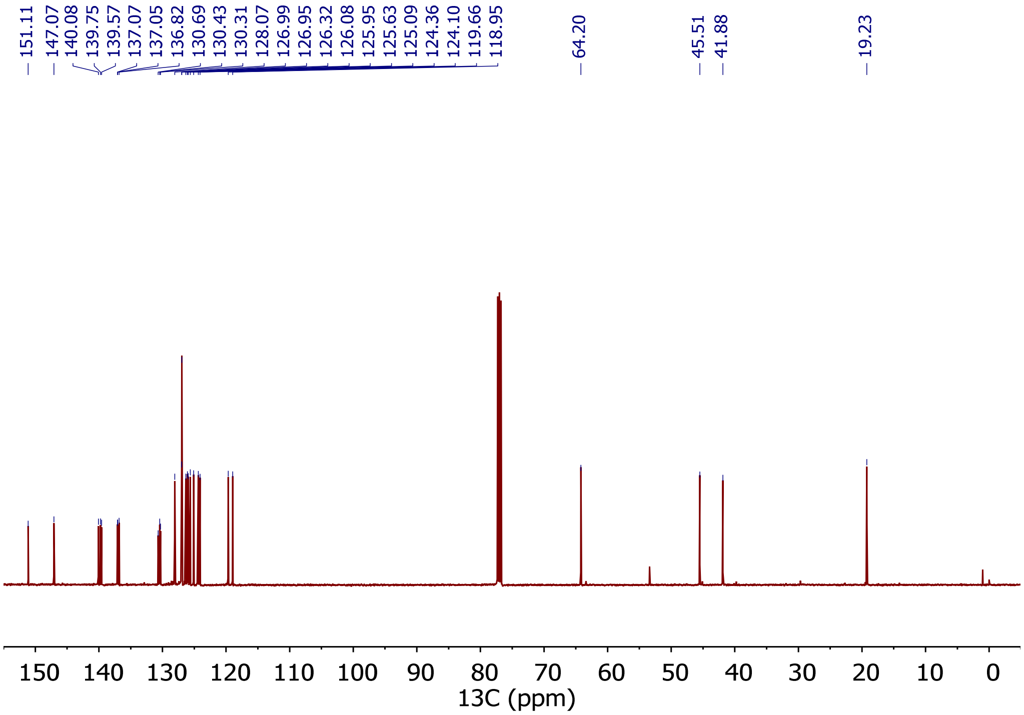

Supplementary Figure 37. NMR spectrum. ^{13}C NMR spectrum of alcohol motor **7** (126 MHz, $CDCl_3$, 298K).

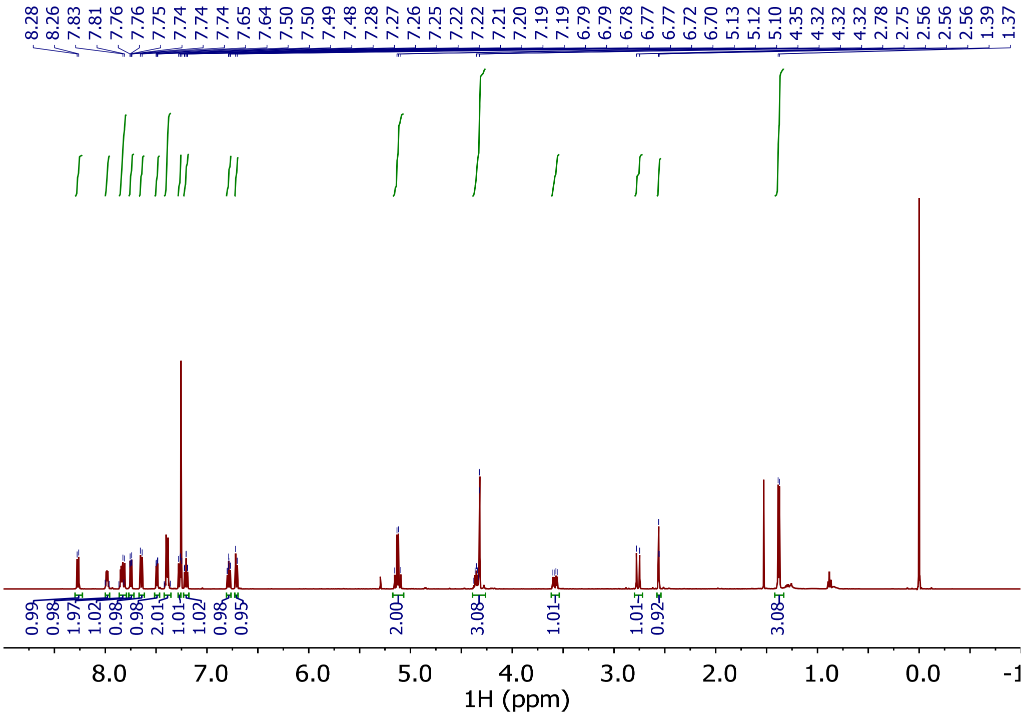

Supplementary Figure 38. NMR spectrum. ^{1}H NMR spectrum of alkyne motor **8** (500 MHz, $CDCl_3$, 298K).

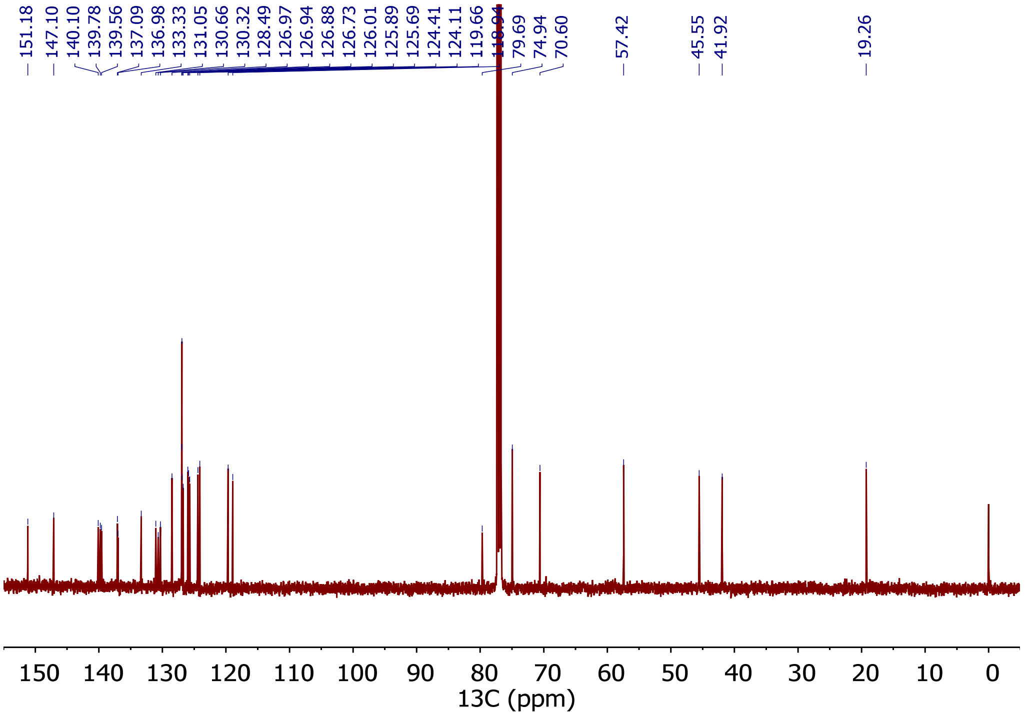

Supplementary Figure 39. NMR spectrum. ^{13}C NMR spectrum of alkyne motor **8** (126 MHz, $CDCl_3$, 298K).

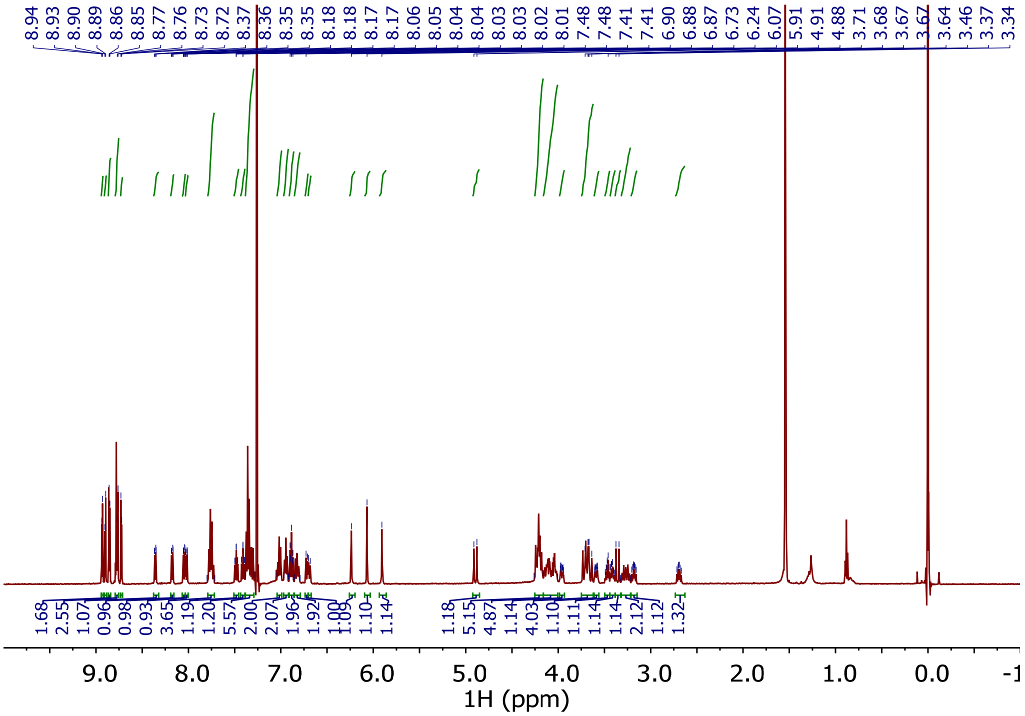

Supplementary Figure 40. NMR spectrum. ^{1}H NMR spectrum of zinc(II) azide cage compound **Zn11** (500 MHz, $CDCl_3$, 298K).

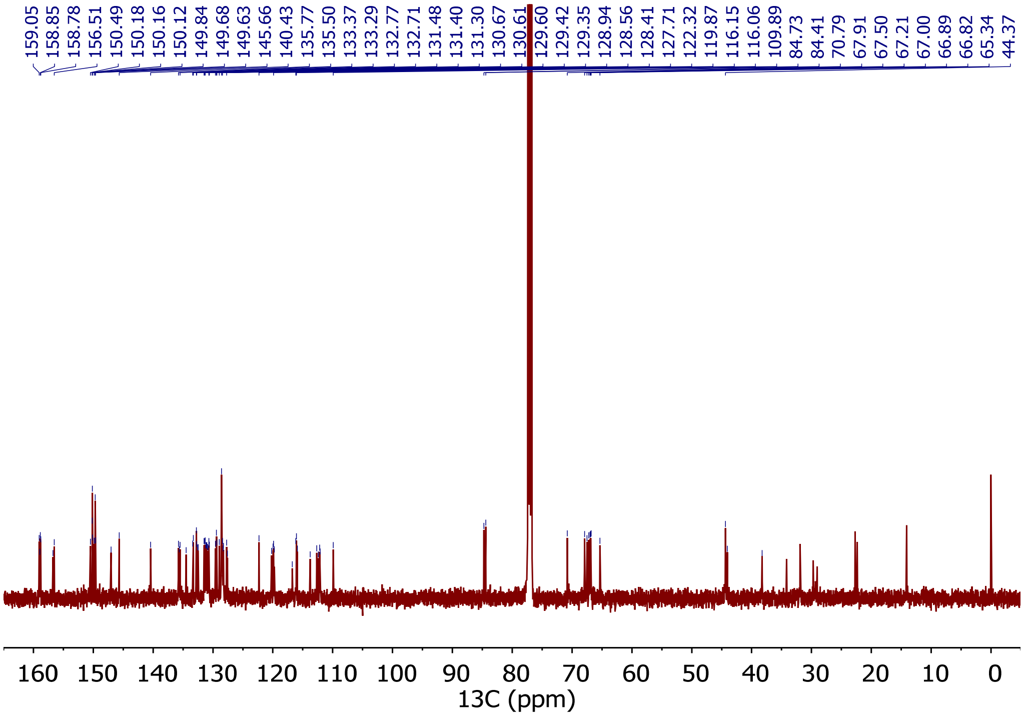

Supplementary Figure 41. NMR spectrum. ^{13}C NMR spectrum of zinc(II) azide cage compound **Zn11** (126 MHz, $CDCl_3$, 298K).

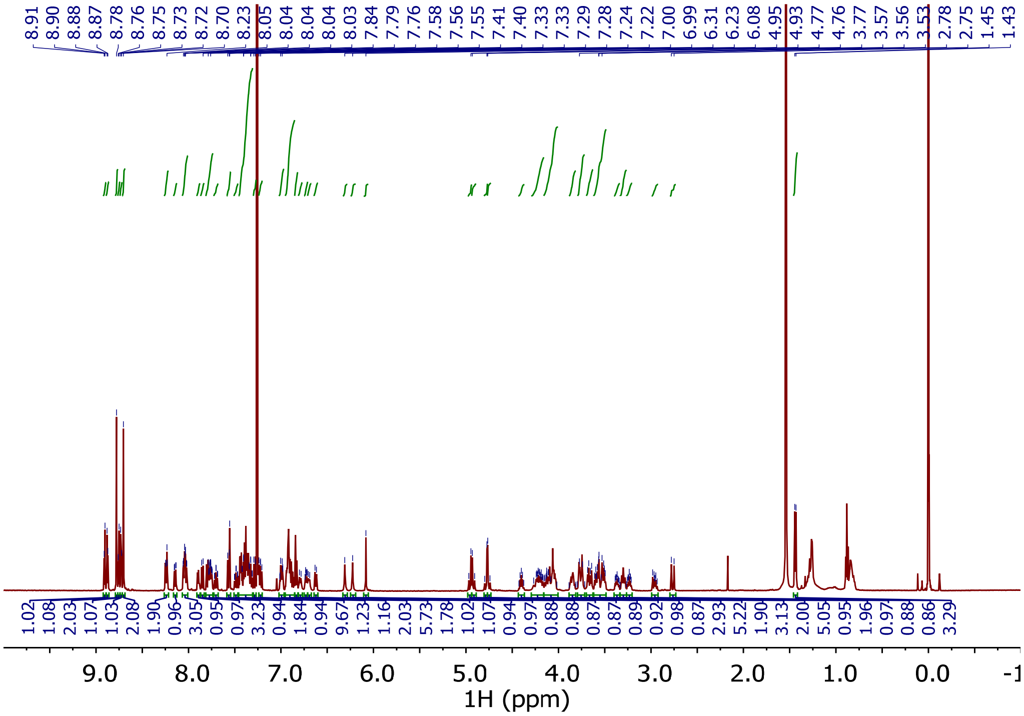

Supplementary Figure 42. NMR spectrum. ^{1}H NMR spectrum of motor–cage **Zn2a** (500 MHz, $CDCl_3$, 298K, c = 10^{-3} M).

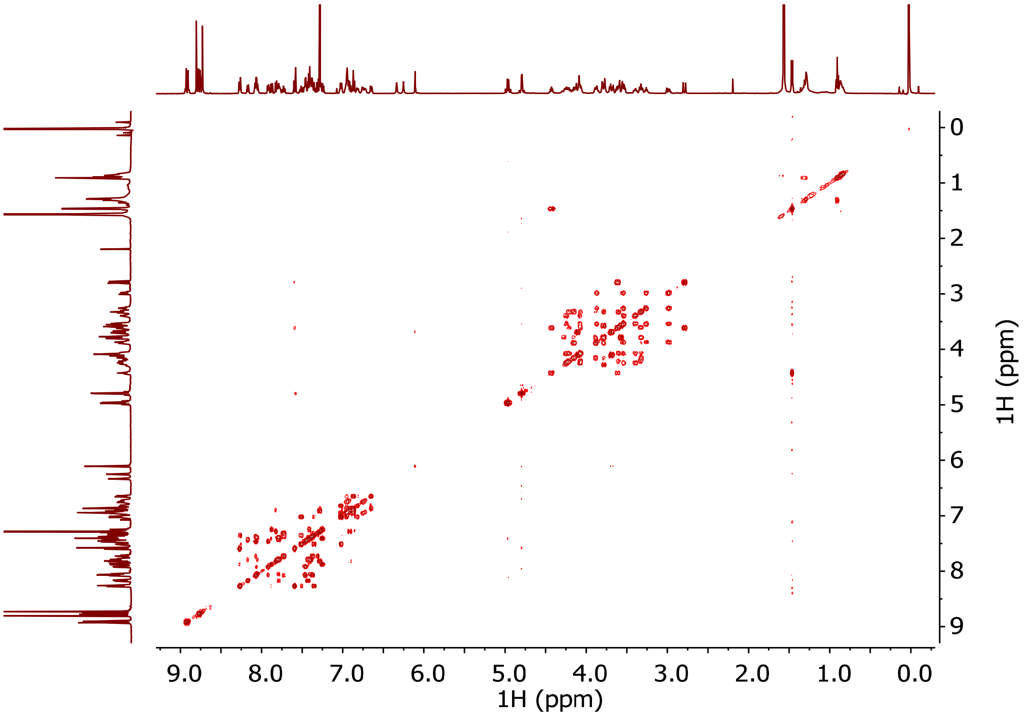

Supplementary Figure 43. 2D-NMR spectrum. ^{1}H-^{1}H COSY NMR spectrum of motor–cage **Zn2a** (500 MHz, $CDCl_3$, 298K, c = 10^{-3} M).

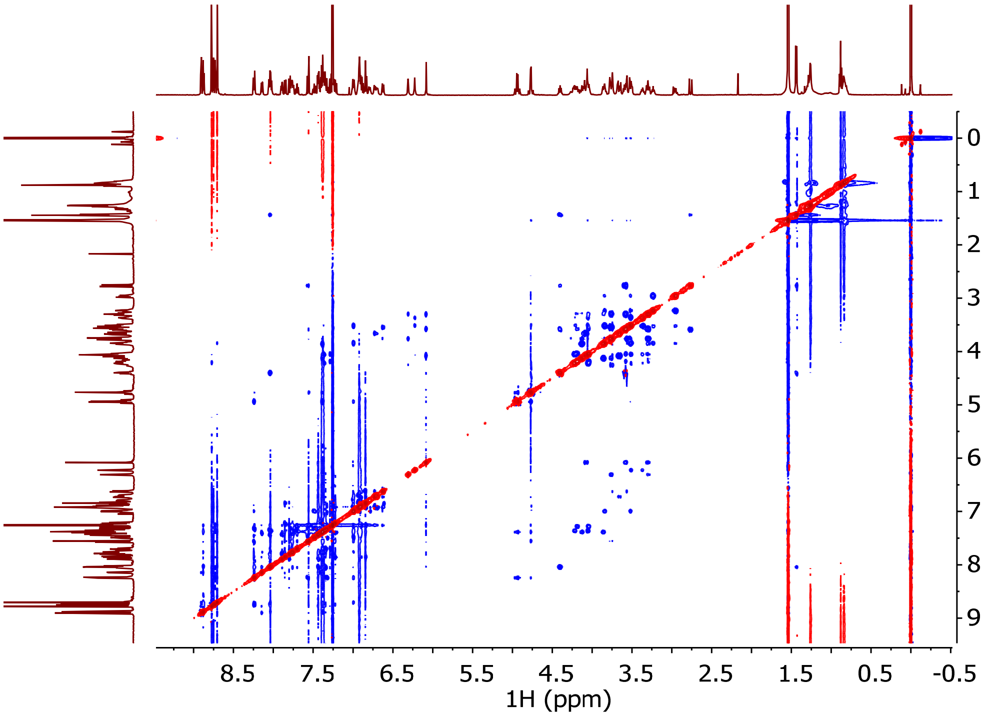

Supplementary Figure 44. 2D-NMR spectrum. ^{1}H-^{1}H ROESY NMR spectrum of motor–cage **Zn2a** (500 MHz, $CDCl_3$, 298K, c = 10^{-3} M).

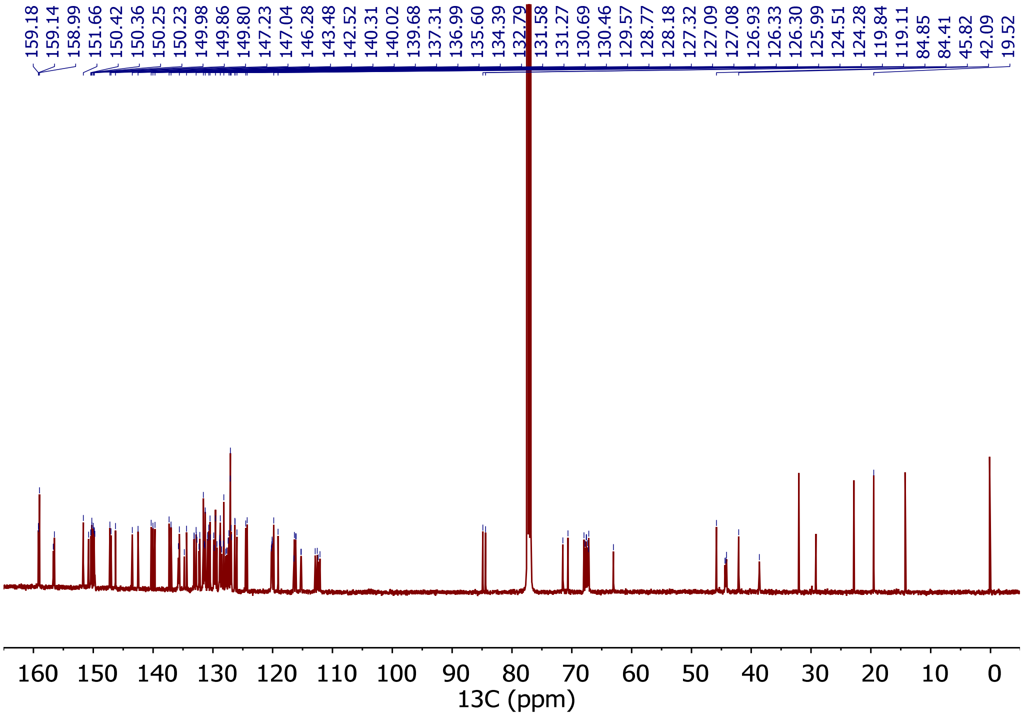

Supplementary Figure 45. NMR spectrum. ^{13}C NMR spectrum of motor–cage **Zn2a** (126 MHz, $CDCl_3$, 298K, c = 10^{-2} M).

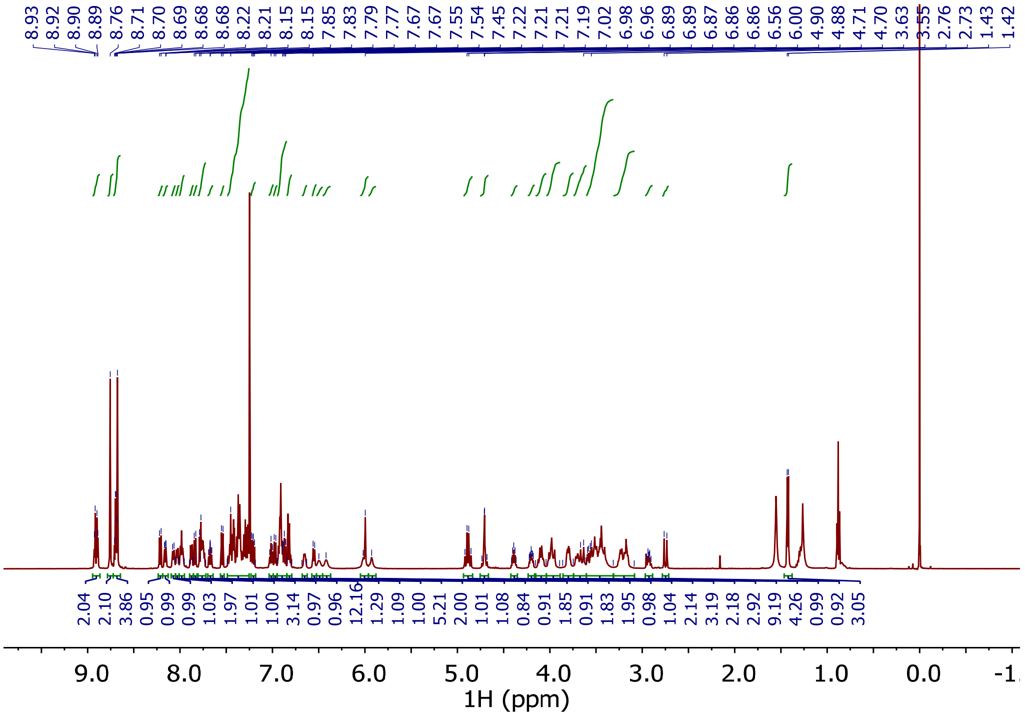

Supplementary Figure 46. NMR spectrum. ^{1}H NMR spectrum of motor–cage **Zn2a** (500 MHz, $CDCl_3$, 298K, c = 10^{-2} M).

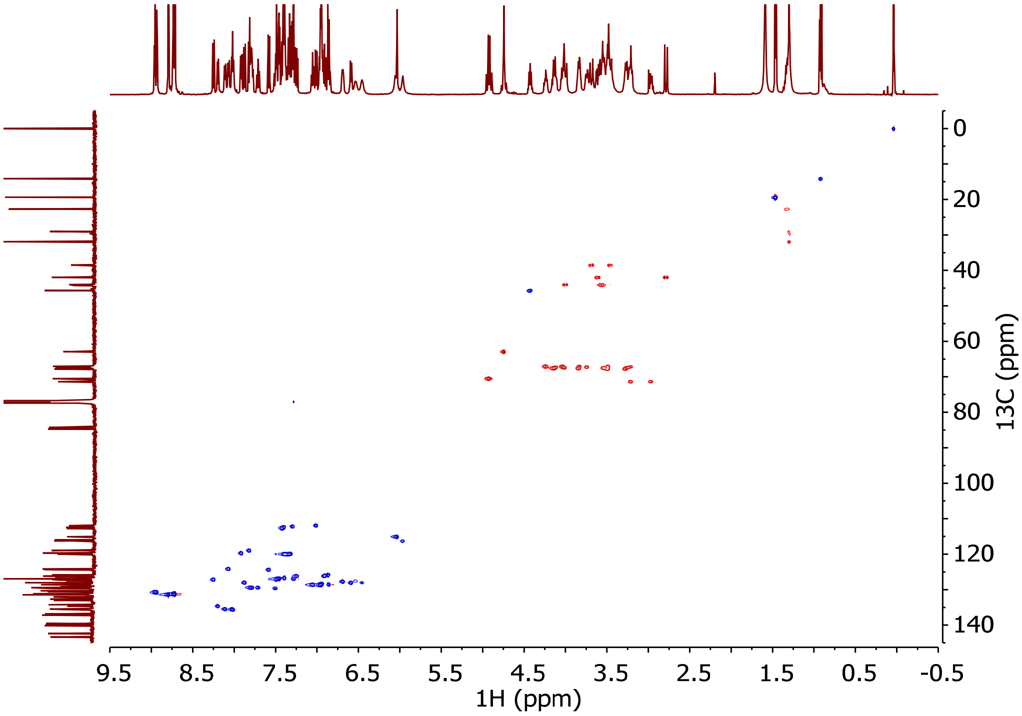

Supplementary Figure 47. 2D-NMR spectrum. ^{1}H-^{13}C HSQC NMR spectrum of motor–cage **Zn2a** (500 MHz, $CDCl_3$, 298K, c = 10^{-2} M). CH_2-groups are indicated in red, and CH/CH_3-groups are indicated in blue.

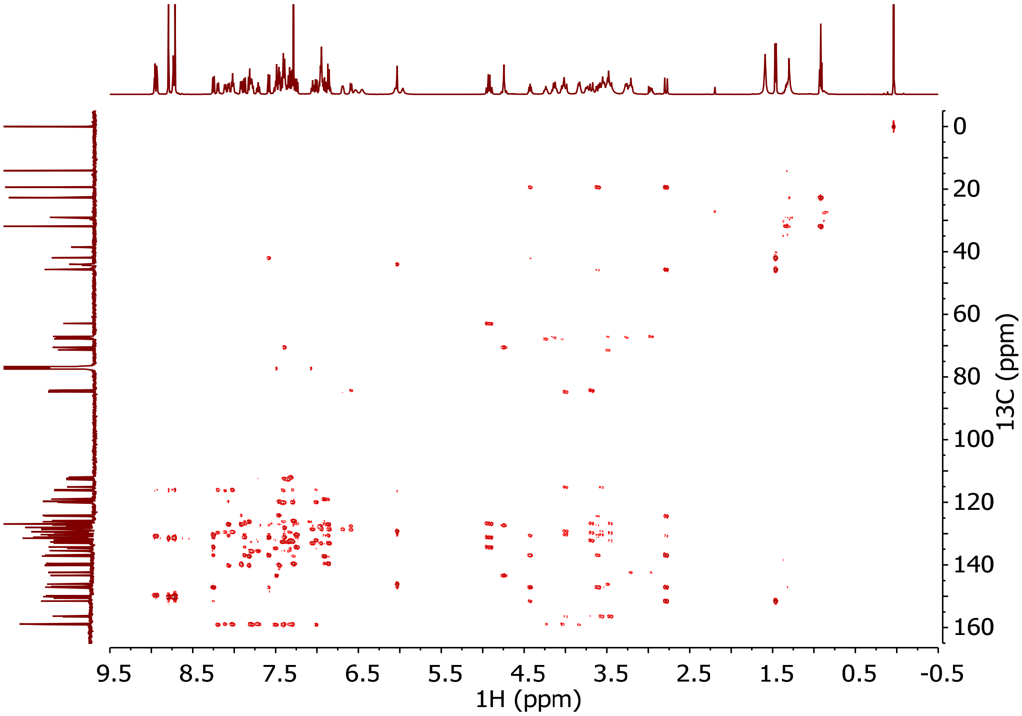

Supplementary Figure 48. 2D-NMR spectrum. ^{1}H-^{13}C HMBC NMR spectrum of motor–cage **Zn2a** (500 MHz, $CDCl_3$, 298K, c = 10^{-2} M).

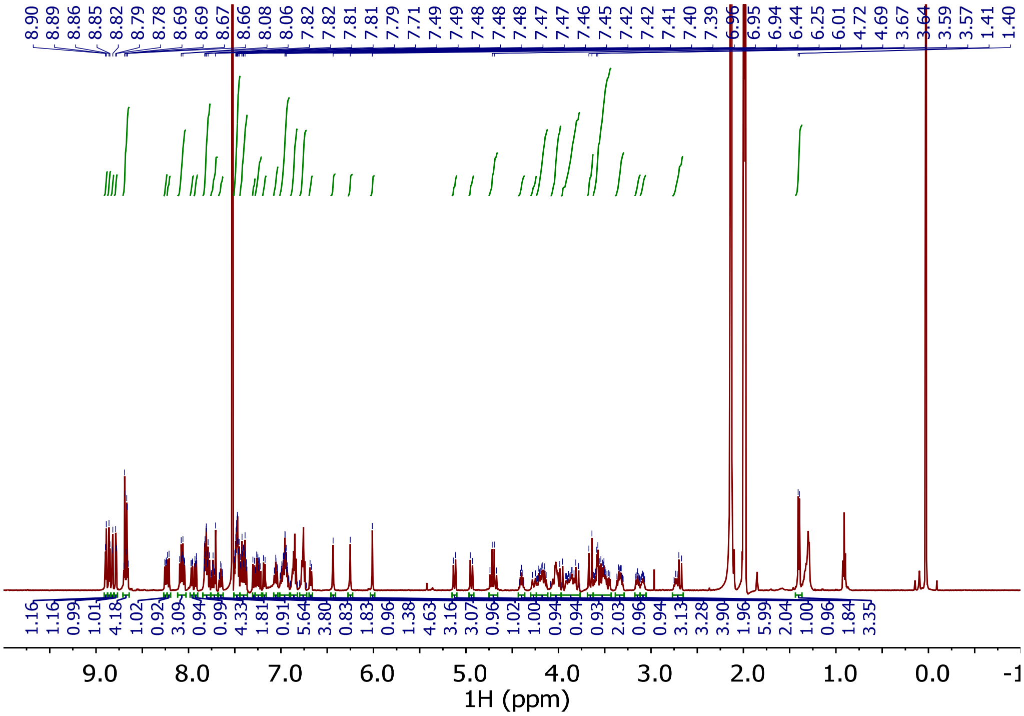

Supplementary Figure 49. NMR spectrum. ^{1}H NMR spectrum of motor–cage **Zn2a** (500 MHz, $CDCl_3/CD_3CN$, 1:1, v/v, 298K, c = 10^{-3} M).

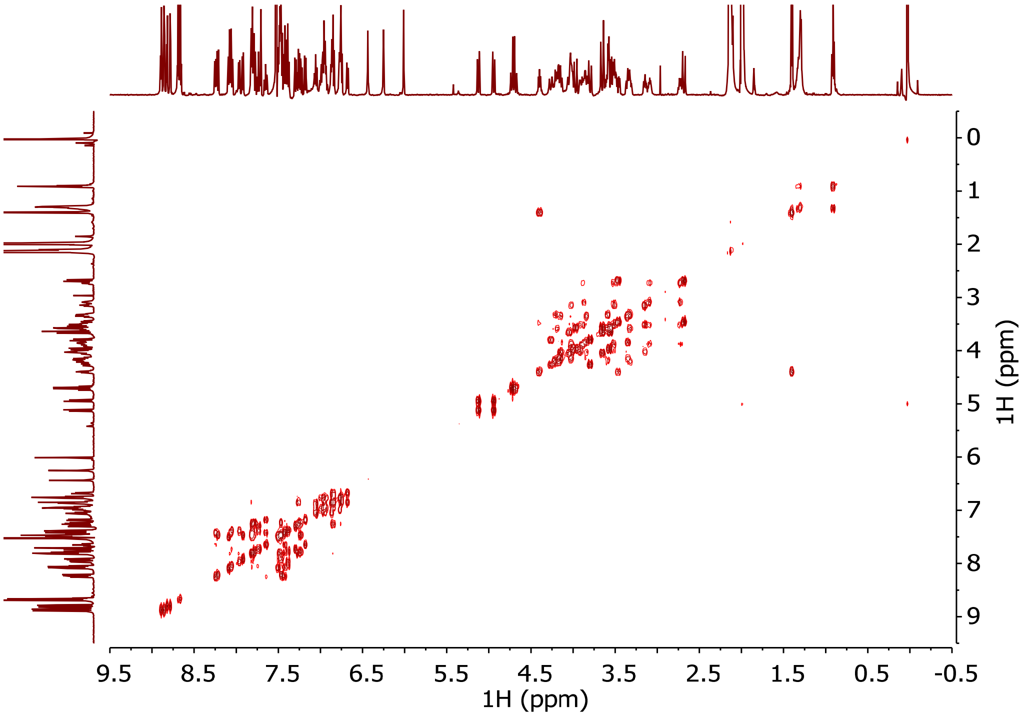

Supplementary Figure 50. 2D-NMR spectrum. ^{1}H-^{1}H COSY NMR spectrum of motor–cage **Zn2a** (500 MHz, $CDCl_3/CD_3CN$, 1:1, v/v, 298K, c = 10^{-3} M).

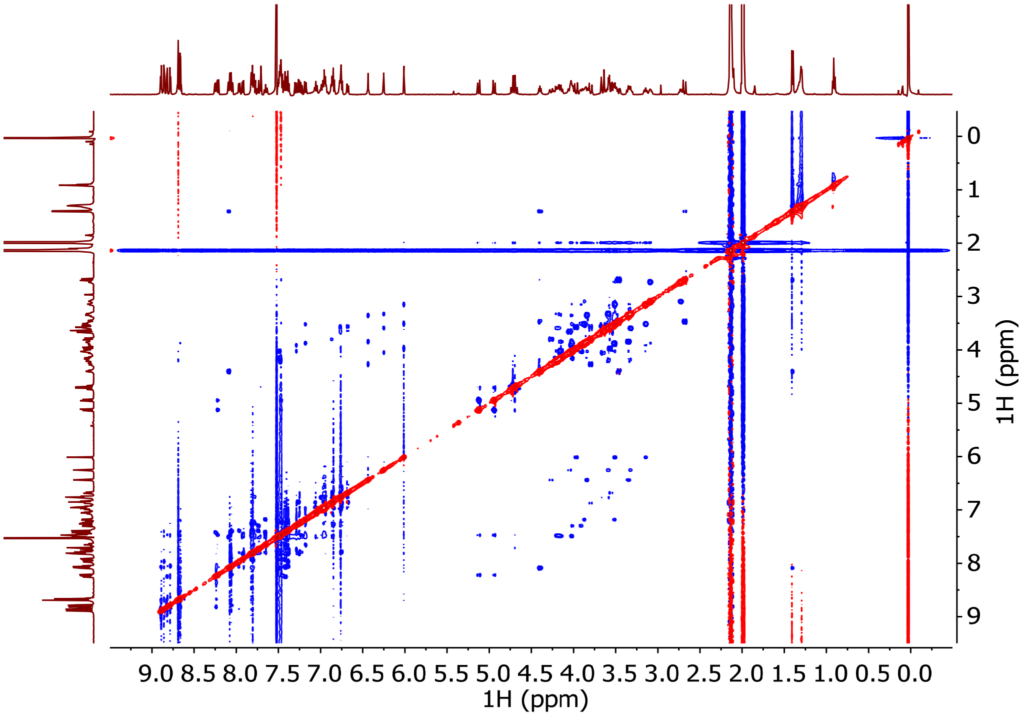

Supplementary Figure 51. 2D-NMR spectrum. ^{1}H-^{1}H ROESY NMR spectrum of motor–cage **Zn2a** (500 MHz, $CDCl_3/CD_3CN$, 1:1, v/v, 298K, c = 10^{-3} M).

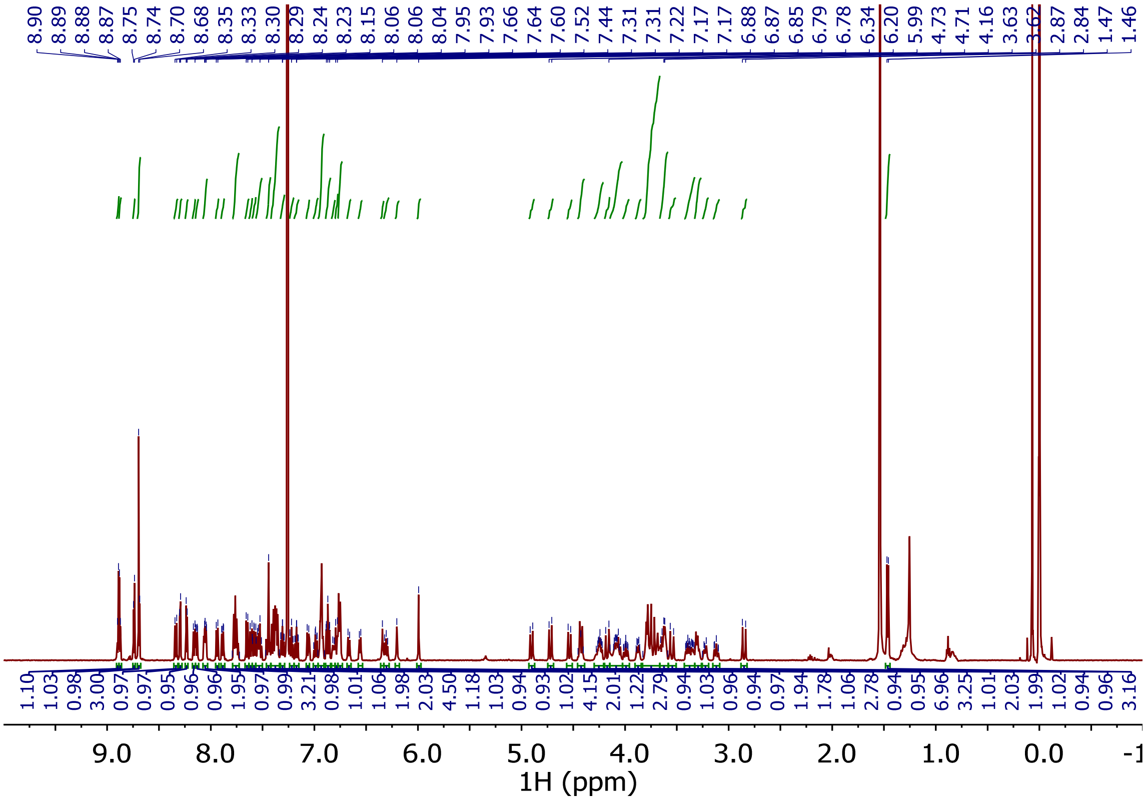

Supplementary Figure 52. NMR spectrum. ^{1}H NMR spectrum of motor–cage **Zn2b** (500 MHz, $CDCl_3$, 298K, c = 10^{-3} M).

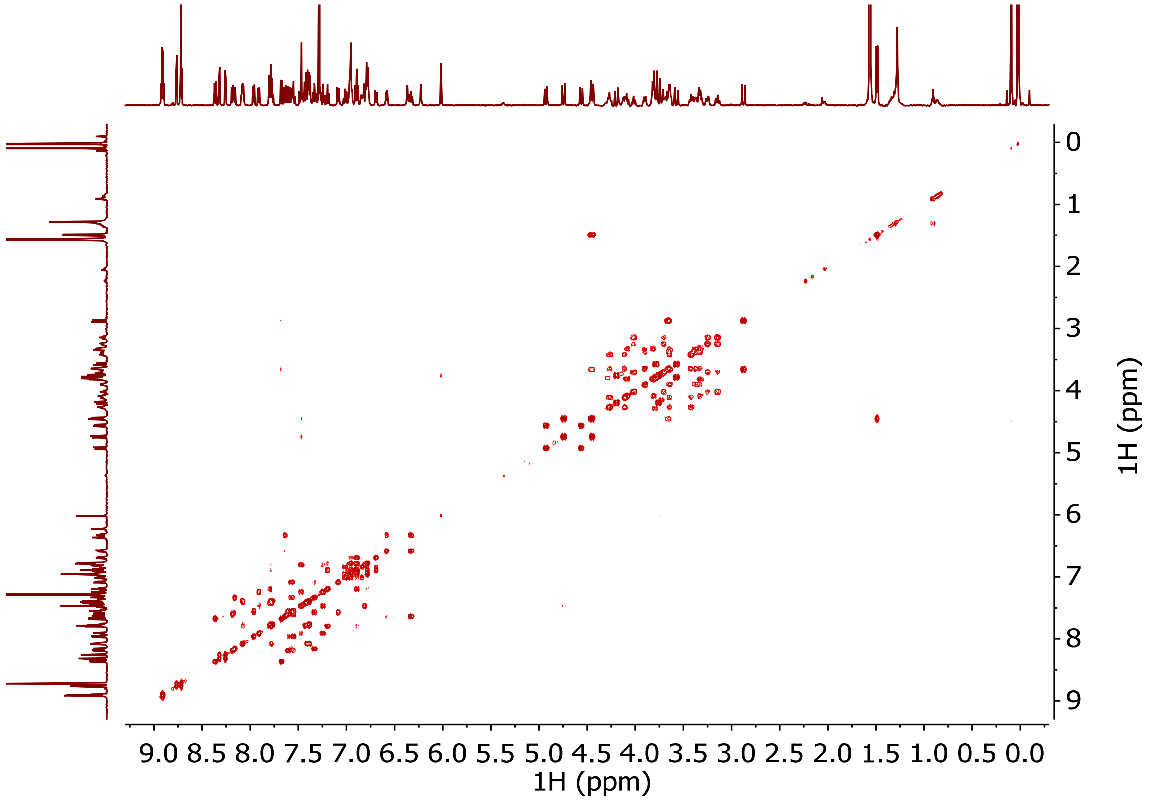

Supplementary Figure 53. 2D-NMR spectrum. ^{1}H-^{1}H COSY NMR spectrum of motor–cage **Zn2b** (500 MHz, $CDCl_3$, 298K, c = 10^{-3} M).

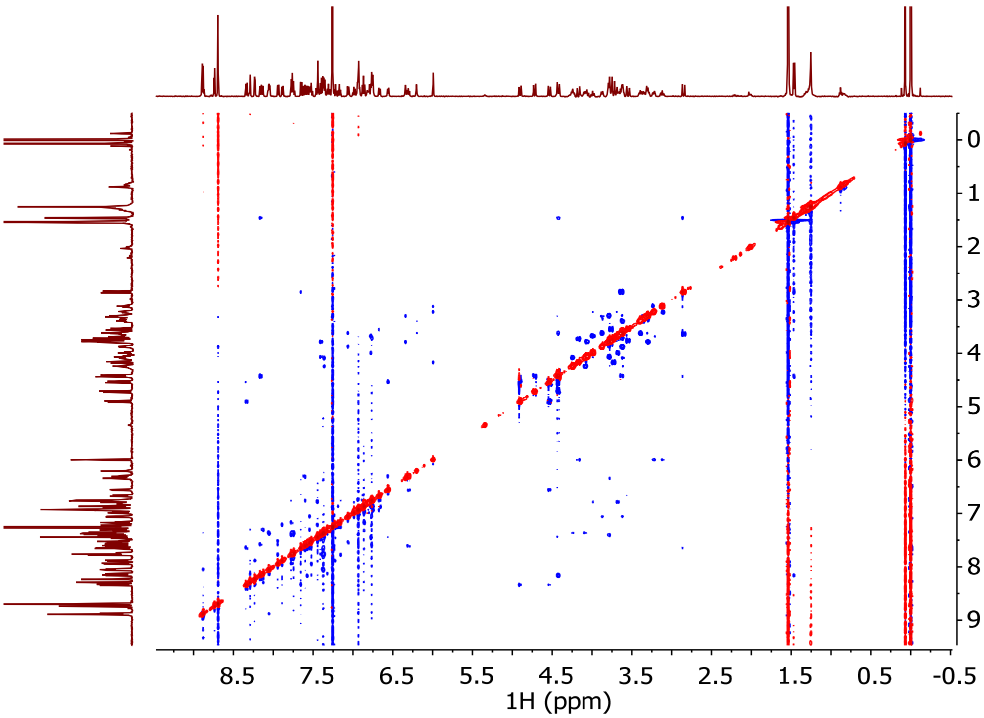

Supplementary Figure 54. 2D-NMR spectrum. ^{1}H-^{1}H ROESY NMR spectrum of motor–cage **Zn2b** (500 MHz, $CDCl_3$, 298K, c = 10^{-3} M).

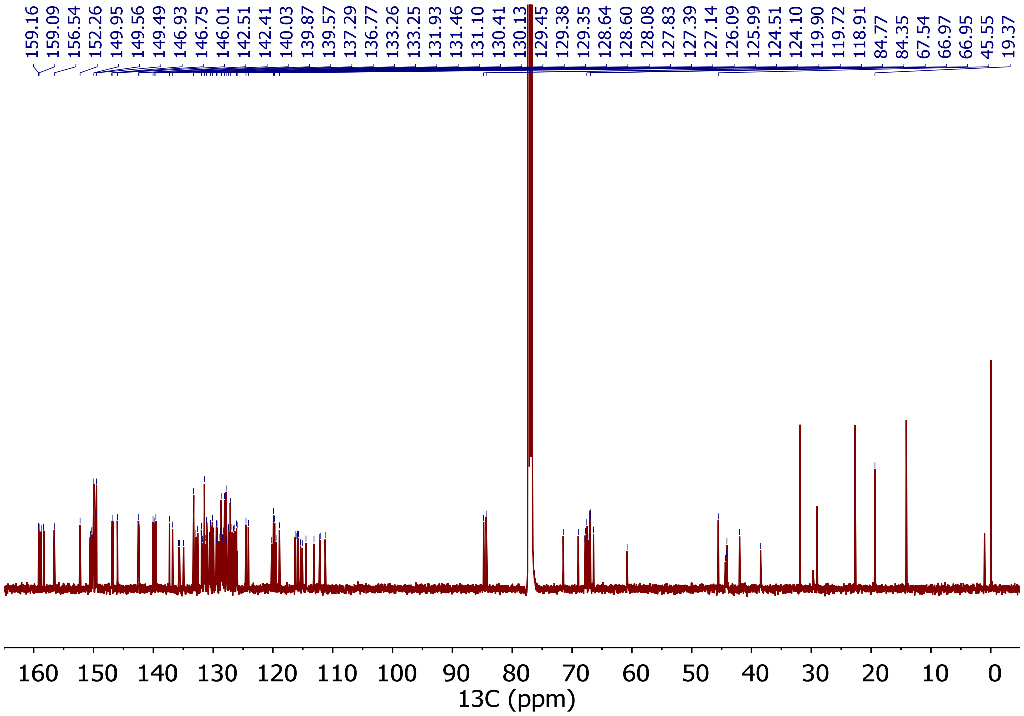

Supplementary Figure 55. NMR spectrum. ^{13}C NMR spectrum of motor–cage **Zn2b** (126 MHz, $CDCl_3$, 298K, c = 10^{-2} M).

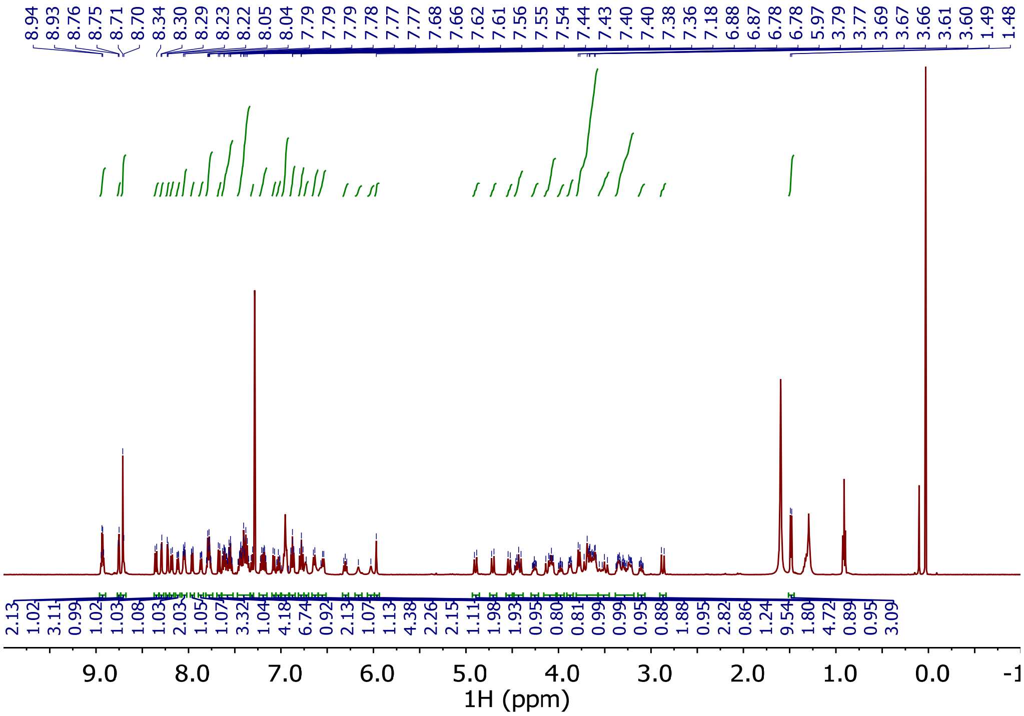

Supplementary Figure 56. NMR spectrum. ^{1}H NMR spectrum of motor–cage **Zn2b** (500 MHz, $CDCl_3$, 298K, c = 10^{-2} M).

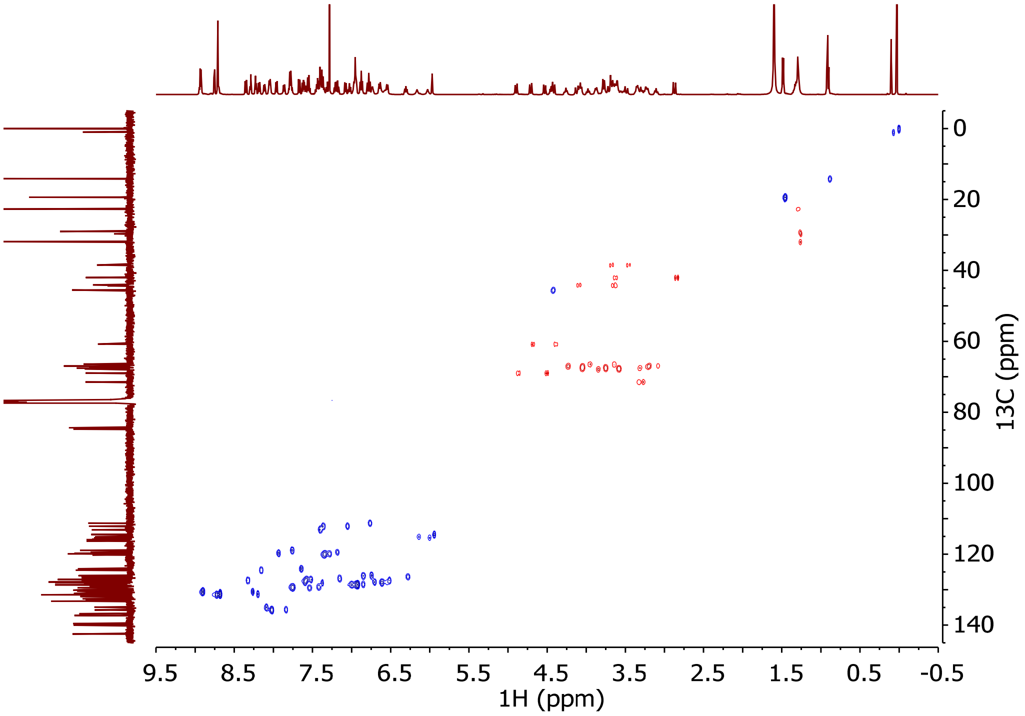

Supplementary Figure 57. 2D-NMR spectrum. ^{1}H-^{13}C HSQC NMR spectrum of motor–cage **Zn2b** (500 MHz, $CDCl_3$, 298K, c = 10^{-2} M). CH_2-groups are indicated in red, and CH/CH_3-groups are indicated in blue.

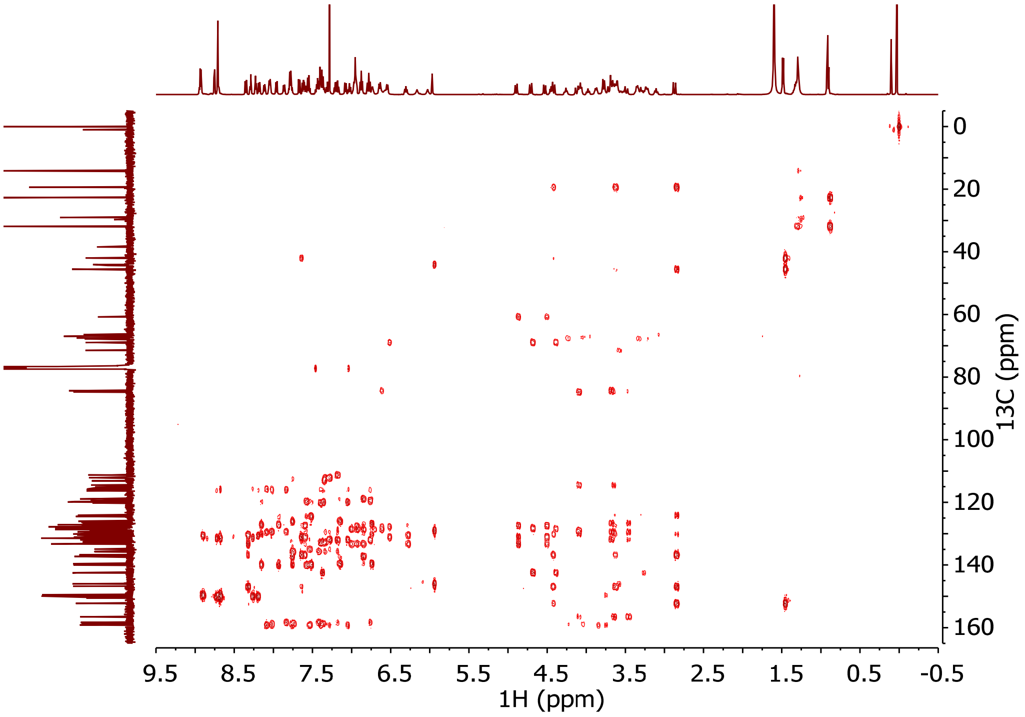

Supplementary Figure 58. 2D-NMR spectrum. ^{1}H-^{13}C HMBC NMR spectrum of motor–cage **Zn2b** (500 MHz, $CDCl_3$, 298K, c = 10^{-2} M).

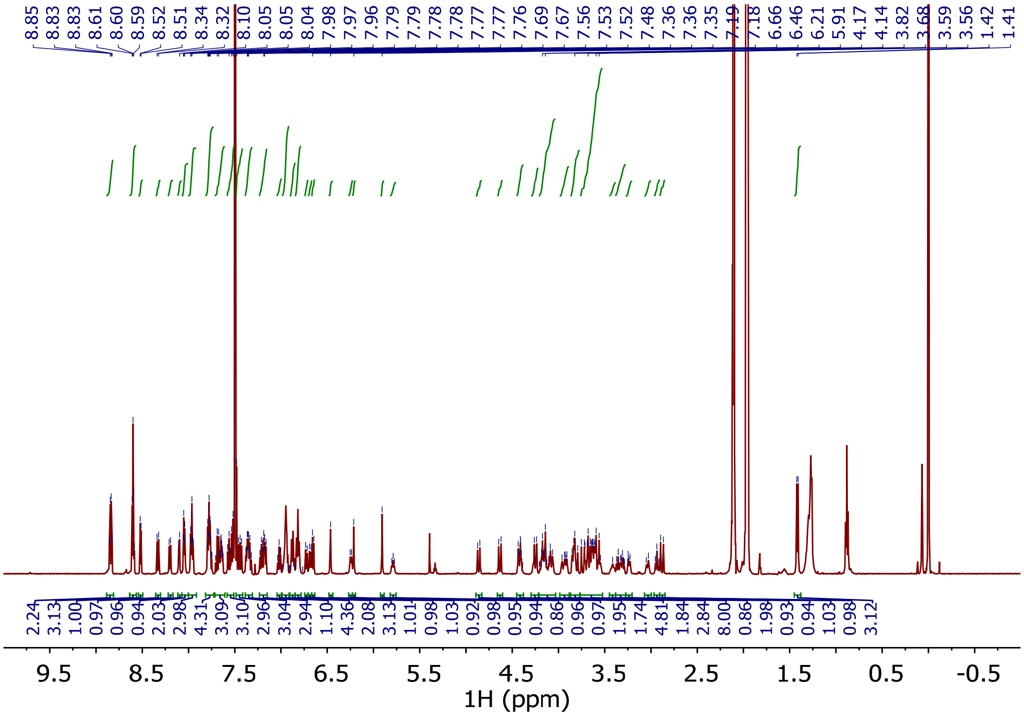

Supplementary Figure 59. NMR spectrum. ^{1}H NMR spectrum of motor–cage **Zn2b** (500 MHz, $CDCl_3/CD_3CN$, 1:1, v/v, 298K, c = 10^{-3} M).

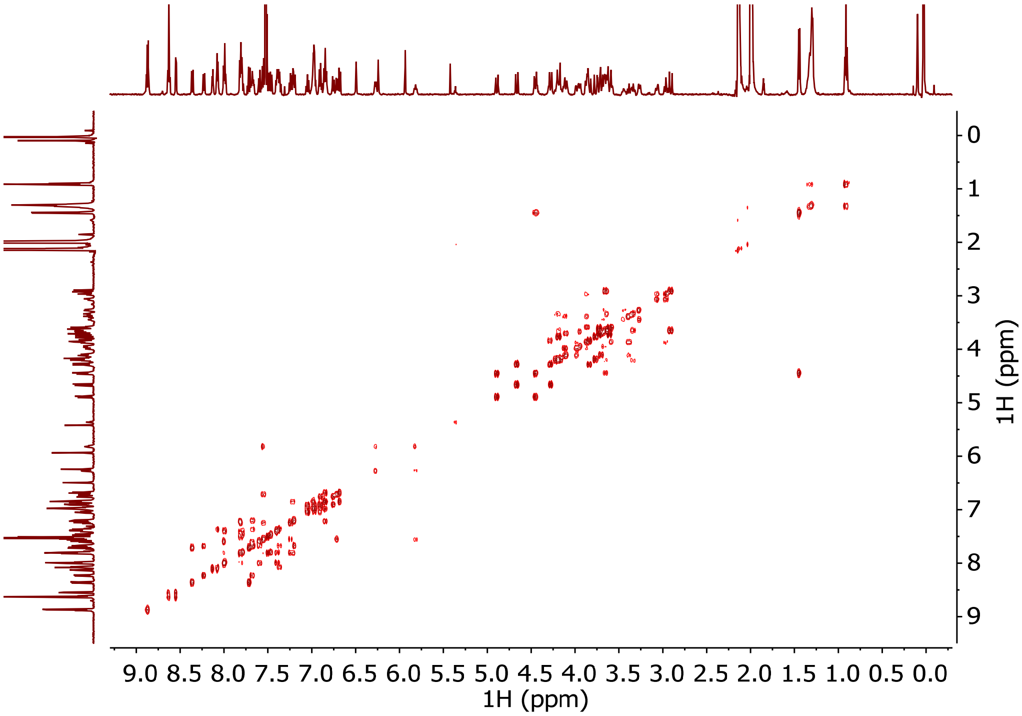

Supplementary Figure 60. 2D-NMR spectrum. ^{1}H-^{1}H COSY NMR spectrum of motor–cage **Zn2b** (500 MHz, $CDCl_3/CD_3CN$, 1:1, v/v, 298K, c = 10^{-3} M).

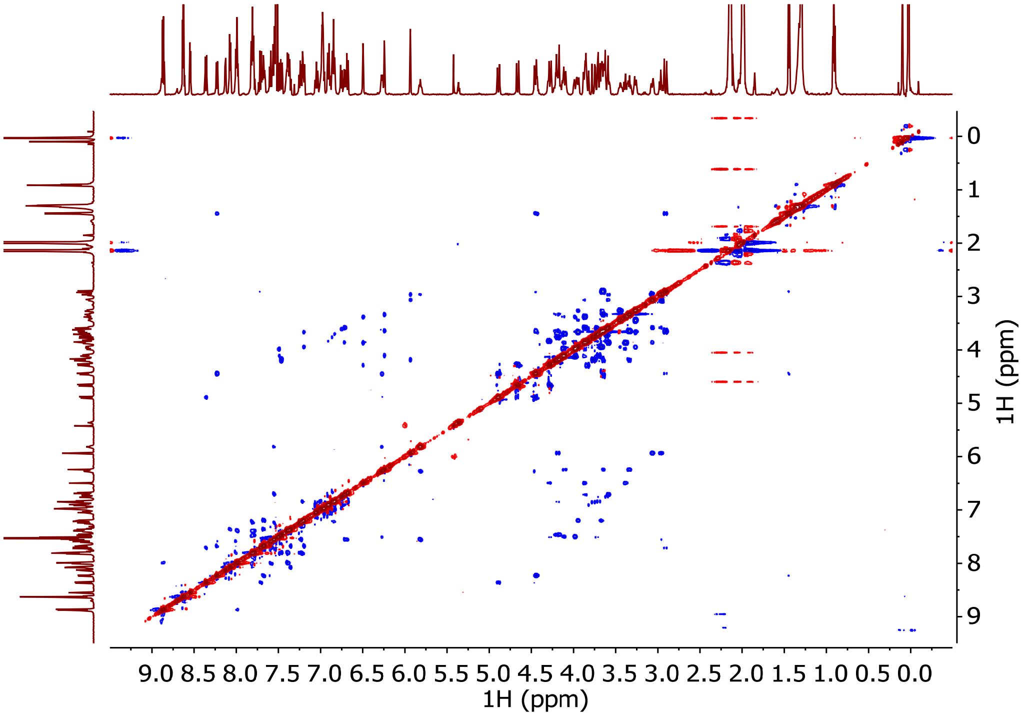

Supplementary Figure 61. 2D-NMR spectrum. ^{1}H-^{1}H ROESY NMR spectrum of motor–cage **Zn2b** (500 MHz, $CDCl_3/CD_3CN$, 1:1, v/v, 298K, c = 10^{-3} M).

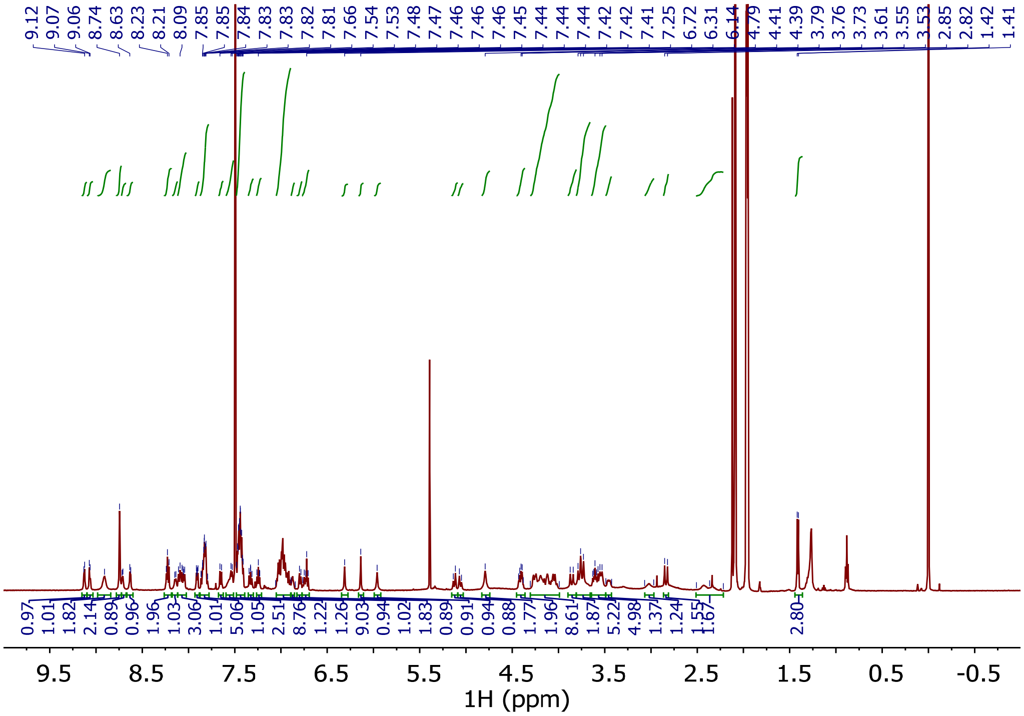

Supplementary Figure 62. NMR spectrum. ^{1}H NMR spectrum of host–guest complex **Zn2a·V12** (500 MHz, $CDCl_3/CD_3CN$, 1:1, v/v, 298K, c = 10^{-3} M).

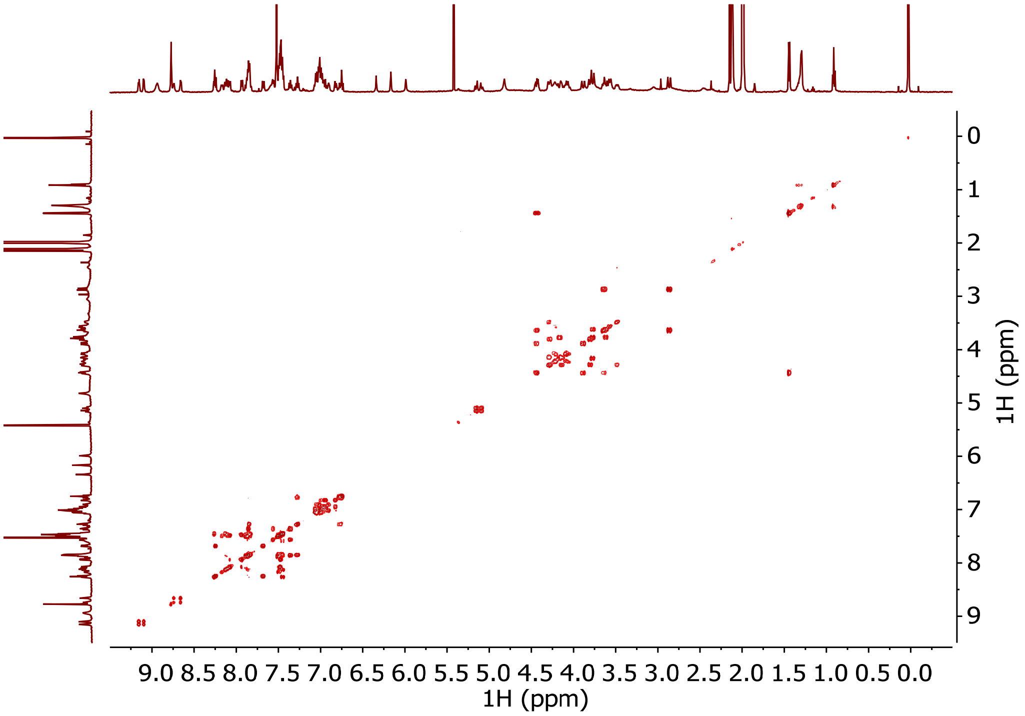

Supplementary Figure 63. 2D-NMR spectrum. ^1H-^1H COSY NMR spectrum of host–guest complex **Zn2a·V12** (500 MHz, $CDCl_3/CD_3CN$, 1:1, v/v, 298K, c = 10^{-3} M).

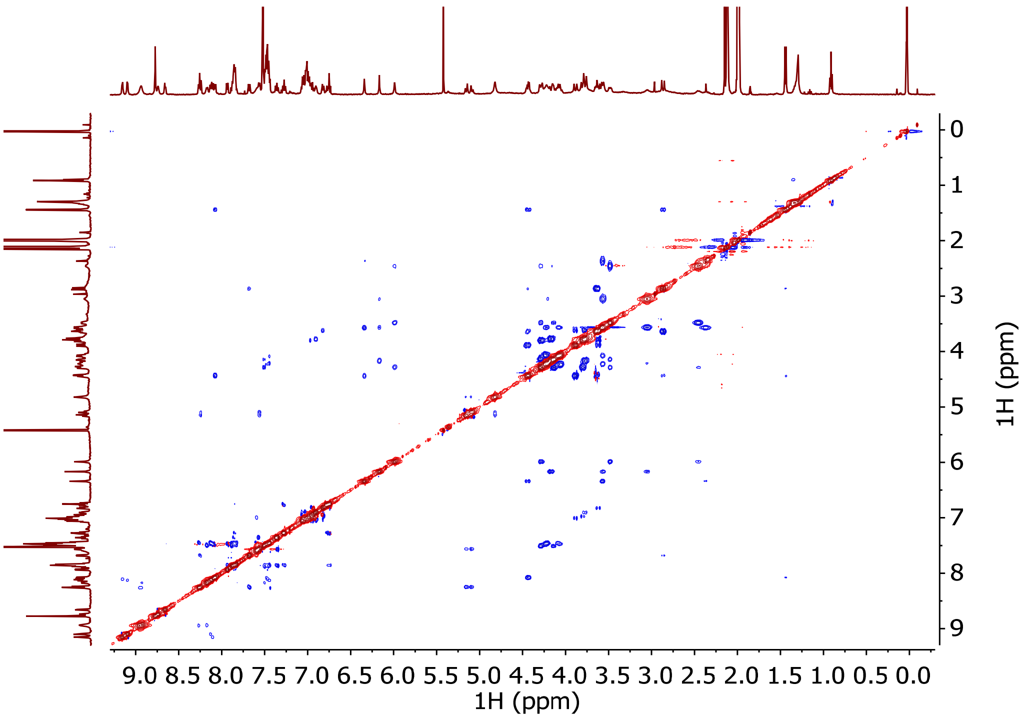

Supplementary Figure 64. 2D-NMR spectrum. ^1H-^1H ROESY NMR spectrum of host–guest complex **Zn2a·V12** (500 MHz, $CDCl_3/CD_3CN$, 1:1, v/v, 298K, c = 10^{-3} M).

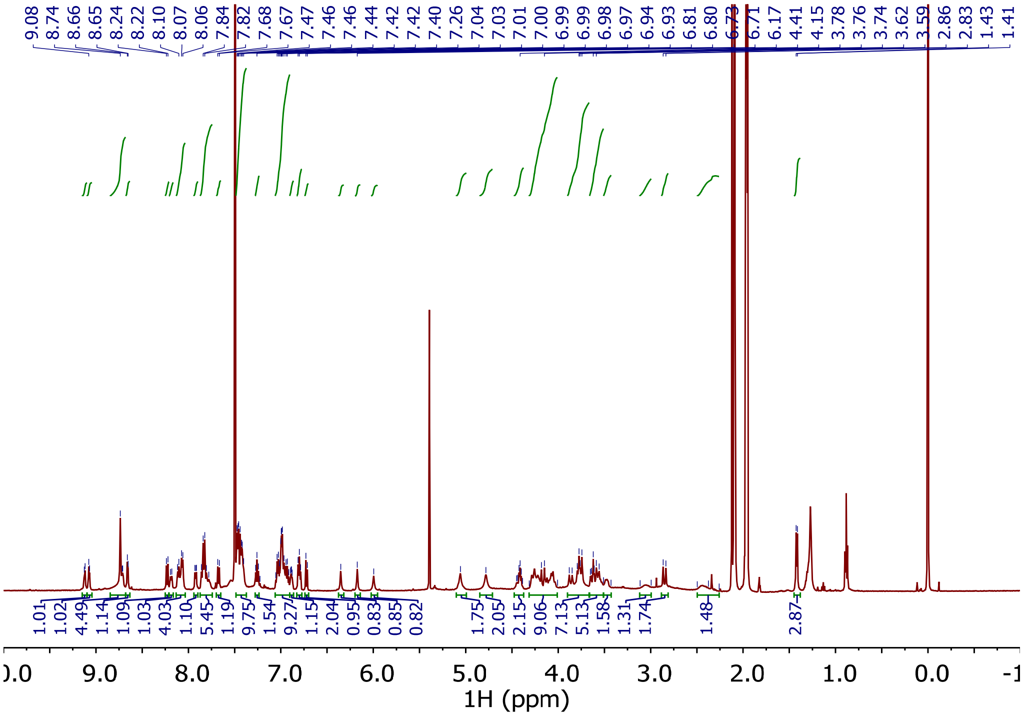

Supplementary Figure 65. NMR spectrum. ^{1}H NMR spectrum of host–guest complex **Zn2b·V12** (500 MHz, $CDCl_3/CD_3CN$, 1:1, v/v, 298K, c = 10^{-3} M).

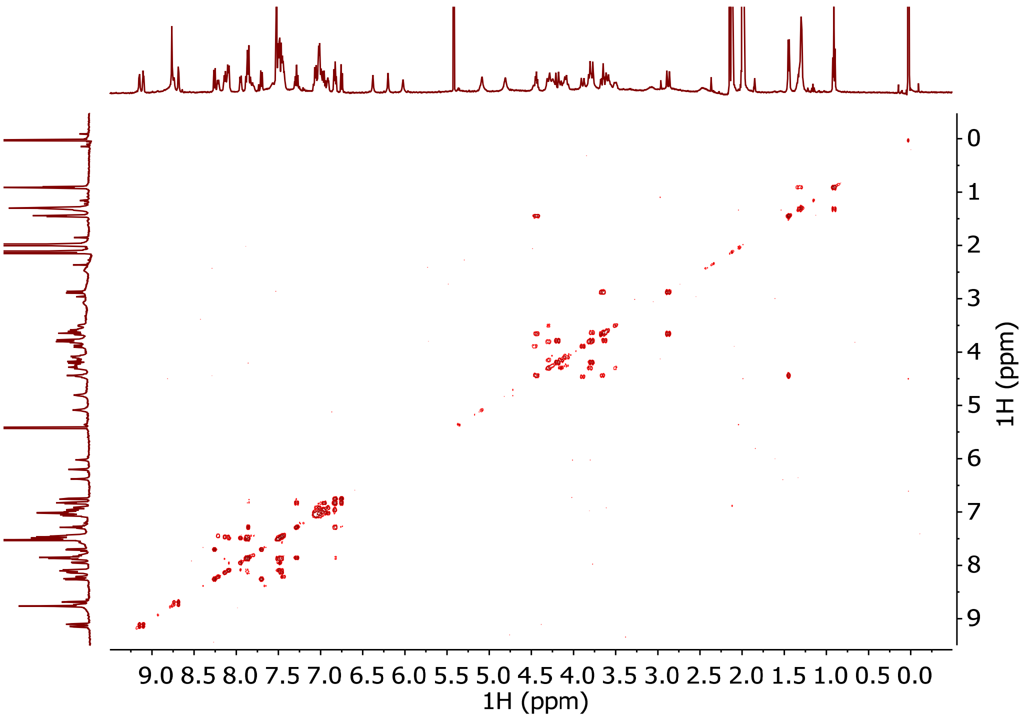

Supplementary Figure 66. 2D-NMR spectrum. ^{1}H-^{1}H COSY NMR spectrum of host–guest complex **Zn2b·V12** (500 MHz, $CDCl_3/CD_3CN$, 1:1, v/v, 298K, c = 10^{-3} M).

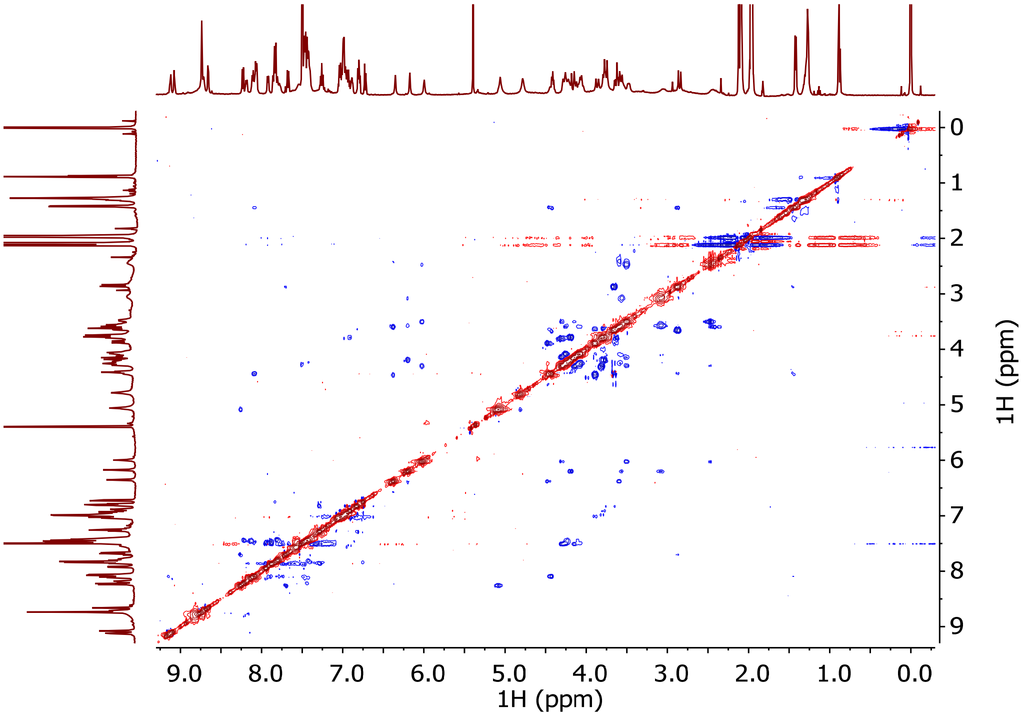

Supplementary Figure 67. 2D-NMR spectrum. ^{1}H-^{1}H ROESY NMR spectrum of host–guest complex **Zn2b·V12** (500 MHz, $CDCl_3/CD_3CN$, 1:1, v/v, 298K, c = 10^{-3} M).

Supplementary References

1. Gilissen, P. J., Swartjes, A., Spierenburg, B., Bruekers, J. P. J., Tinnemans, P., White, P. B., Rutjes, F. P. J. T., Nolte, R. J. M. & Elemans, J. A. A. W. Rapid and scalable synthesis of chiral porphyrin cage compounds. *Tetrahedron* **75,** 4640–4647 (2019).

2. Elemans, J. A. A. W., Claase, M. B., Aarts, P. P. M., Rowan, A. E., Schenning, A. P. H. J. & Nolte, R. J. M. Porphyrin clips derived from diphenylglycoluril. Synthesis, conformational analysis, and binding properties. *J. Org. Chem.* **64,** 7009–7016 (1999).

3. Thordarson, P., Bijsterveld, E. J. A., Rowan, A. E. & Nolte, R. J. M. Epoxidation of polybutadiene by a topologically linked catalyst. *Nature* **424,** 915–918 (2003).

4. Bauer, J., Hou, L., Kistemaker, J. C. M. & Feringa, B. L. Tuning the rotation rate of light-driven molecular motors. *J. Org. Chem.* **79,** 4446–4455 (2014).

5. Chen, K.-Y., Ivashenko, O., Carroll, G. T., Robertus, J., Kistemaker, J. C. M., London, G., Browne, W. R., Rudolf, P. & Feringa, B. L. Control of surface wettability using tripodal light-activated molecular motors. *J. Am. Chem. Soc.* **136,** 3219–3224 (2014).

6. Chen, J., Kistemaker, J. C. M., Robertus, J. & Feringa, B. L. Molecular stirrers in action. *J. Am. Chem. Soc.* **136,** 14924–14932 (2014).

7. Varghese, S., Spierenburg, B., Swartjes, A., White, P.B., Tinnemans, P., Elemans, J.A.A.W. & Nolte, R. J. M. Direct synthesis of chiral porphyrin macrocyclic receptors via regioselective nitration. *Org. Lett.* **20,** 3719–3722 (2018).

8. Yu, L., Sun, J., Wang, Q., Guan, Y., Zhou, L., Zhang, J., Zhang, L. & Yang, H. Reprogrammable assembly of molecular motor on solid surfaces via dynamic bonds. *Small* **13,** 1700480 (2017).

9. Online tools for supramolecular chemistry research and analysis. http://supramolecular.org (last accessed July 14, 2020).

10. Hibbert, D. B. & Thordarson, P. The death of the Job plot, transparency, open science and online tools, uncertainty estimation methods and other developments in supramolecular chemistry data analysis. *Chem. Commun.* **52,** 12792–12805 (2016).
